# A systematic review of the psychometric properties of self-report research utilization measures used in healthcare

**DOI:** 10.1186/1748-5908-6-83

**Published:** 2011-07-27

**Authors:** Janet E Squires, Carole A Estabrooks, Hannah M O'Rourke, Petter Gustavsson, Christine V Newburn-Cook, Lars Wallin

**Affiliations:** 1 Clinical Epidemiology Program, Ottawa Hospital Research Institute, Ottawa, Canada; 2 Faculty of Nursing, University of Alberta, Edmonton, Canada; 3 Department of Clinical Neuroscience (Division of Psychology), Karolinska Institutet, Stockholm, Sweden; 4 Department of Neurobiology, Care Sciences and Society (Division of Nursing), Karolinska Institutet, Stockholm, Sweden

## Abstract

**Background:**

In healthcare, a gap exists between what is known from research and what is practiced. Understanding this gap depends upon our ability to robustly measure research utilization.

**Objectives:**

The objectives of this systematic review were: to identify self-report measures of research utilization used in healthcare, and to assess the psychometric properties (acceptability, reliability, and validity) of these measures.

**Methods:**

We conducted a systematic review of literature reporting use or development of self-report research utilization measures. Our search included: multiple databases, ancestry searches, and a hand search. Acceptability was assessed by examining time to complete the measure and missing data rates. Our approach to reliability and validity assessment followed that outlined in the *Standards for Educational and Psychological Testing*.

**Results:**

Of 42,770 titles screened, 97 original studies (108 articles) were included in this review. The 97 studies reported on the use or development of 60 unique self-report research utilization measures. Seven of the measures were assessed in more than one study. Study samples consisted of healthcare providers (92 studies) and healthcare decision makers (5 studies). No studies reported data on acceptability of the measures. Reliability was reported in 32 (33%) of the studies, representing 13 of the 60 measures. Internal consistency (Cronbach's Alpha) reliability was reported in 31 studies; values exceeded 0.70 in 29 studies. Test-retest reliability was reported in 3 studies with Pearson's *r *coefficients > 0.80. No validity information was reported for 12 of the 60 measures. The remaining 48 measures were classified into a three-level validity hierarchy according to the number of validity sources reported in 50% or more of the studies using the measure. Level one measures (n = 6) reported evidence from any three (out of four possible) *Standards *validity sources (which, in the case of single item measures, was all applicable validity sources). Level two measures (n = 16) had evidence from any two validity sources, and level three measures (n = 26) from only one validity source.

**Conclusions:**

This review reveals significant underdevelopment in the measurement of research utilization. Substantial methodological advances with respect to construct clarity, use of research utilization and related theory, use of measurement theory, and psychometric assessment are required. Also needed are improved reporting practices and the adoption of a more contemporary view of validity (*i.e.*, the *Standards*) in future research utilization measurement studies.

## Background

Clinical and health services research produces vast amounts of new research every year. Despite increased access by healthcare providers and decision-makers to this knowledge, uptake into practice is slow [1,2] and has resulted in a 'research-practice gap.'

### Measuring research utilization

Recognition of, and a desire to narrow, the research-practice gap, has led to the accumulation of a considerable body of knowledge on research utilization and related terms, such as knowledge translation, knowledge utilization, innovation adoption, innovation diffusion, and research implementation. Despite gains in the understanding of research utilization theoretically [3,4], a large and rapidly expanding literature addressing the individual factors associated with research utilization [5,6], and the implementation of clinical practice guidelines in various health disciplines [7,8], little is known about how to robustly measure research utilization.

We located three theoretical papers explicitly addressing the measurement of knowledge utilization (of which research utilization is a component) [9-11], and one integrative review that examined the psychometric properties of self-report research utilization measures used in professions allied to medicine [12]. Within each of these papers, a need for conceptual clarity and pluralism in measurement was stressed. Weiss [11] also argued for specific foci (*i.e*., focus on specific studies, people, issues, or organizations) when measuring knowledge utilization. Shortly thereafter, Dunn [9], proposed a linear four-step process for measuring knowledge utilization: conceptualization (what is knowledge utilization and how it is defined and classified); methods (given a particular conceptualization, what methods are available to observe knowledge use); measures (what scales are available to measure knowledge use); and reliability and validity. Dunn specifically urged that greater emphasis be placed on step four (reliability and validity). A decade later, Rich [10] provided a comprehensive overview of issues influencing knowledge utilization across many disciplines. He emphasized the complexity of the measurement process, suggesting that knowledge utilization may not always be tied to a specific action, and that it may exist as more of an omnibus concept.

The only review of research utilization measures to date was conducted in 2003 by Estabrooks *et al. *[12]. The review was limited to self-report research utilization measures used in professions allied to medicine and to the specific data on validity that was extracted. That is, only data that was (by the original authors) explicitly interpreted as validity in the study reports was extracted as 'supporting validity evidence'. A total of 43 articles from three online databases (CINAHL, Medline, and Pubmed) comprised the final sample of articles included in the review. Two commonly used multi-item self-report measures (published in 16 papers) were identified--the Nurses Practice Questionnaire and the Research Utilization Questionnaire. An additional 16 published papers were identified that used single-item self-report questions to measure research utilization. Several problems with these research utilization measures were identified: lack of construct clarity of research utilization, lack of use of research utilization theories, lack of use of measurement theory, and finally, lack of standard psychometric assessment.

The four papers [9-12] discussed above point to a persistent and unresolved problem--an inability to robustly measure research utilization. This presents both an important and a practical challenge to researchers and decision-makers who rely on such measures to evaluate the uptake and effectiveness of research findings to improve patient and organizational outcomes. There are multiple reasons why we believe the measurement of research utilization is important. The most important reason relates to designing and evaluating the effectiveness of interventions to improve patient outcomes. Research utilization is commonly assumed to have a positive impact on patient outcomes by assisting with eliminating ineffective and potentially harmful practices, and implementing more effective (research-based) practices. However, we can only determine if patient outcomes are sensitive to varying levels of research utilization if we can first measure research utilization in a reliable and valid manner. If patient outcomes are sensitive to the use of research and we do not measure it, we, in essence, do the field more harm than good by ignoring a 'black box' of causal mechanisms that can influence research utilization. The causal mechanisms within this back box can, and should, be used to inform the design of interventions that aim to improve patient outcomes by increasing research utilization by care providers.

### Study purpose and objectives

The study reported in this paper is a systematic review of the psychometric properties of self-report measures of research utilization used in healthcare. Specific objectives of this study were to: identify self-report measures of research utilization used in healthcare (*i.e*., used to measure research utilization by healthcare providers, healthcare decision makers, and in healthcare organizations); and assess the psychometric properties of these measures.

## Methods

### Study selection (inclusion and exclusion) criteria

Studies were included that met the following inclusion criteria: reported on the development or use of a self-report measure of research utilization; and the study population comprised one or more of the following groups--healthcare providers, healthcare decision makers, or healthcare organizations. We defined research utilization as the use of research-based (empirically derived) information. This information could be reported in a primary research article, review/synthesis report, or a protocol. Where the study involved the use of a protocol, we required the research-basis for the protocol to be apparent in the article. We excluded articles that reported on adherence to clinical practice guidelines, the rationale being that clinical practice guidelines can be based on non-research evidence (*e.g*., expert opinion). We also excluded articles reporting on the use of one specific-research-based practice if the overall purpose of the study was not to examine research utilization.

### Search strategy for identification of studies

We searched 12 bibliographic databases; details of the search strategy are located in Additional File 1. We also hand searched the journal *Implementation Science *(a specialized journal in the research utilization field) and assessed the reference lists of all retrieved articles. The final set of included articles was restricted to those published in the English, Danish, Swedish, and Norwegian languages (the official languages of the research team). There were no restrictions based on when the study was undertaken or publication status.

### Selection of Studies

**Figure 1:**
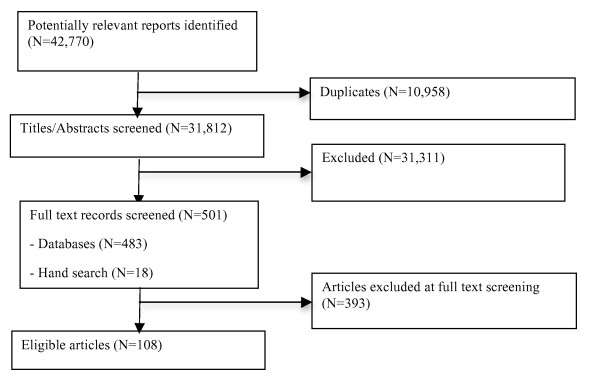
**Article screening and selection**.

Two team members (JES and HMO) independently screened all titles and abstracts (n = 42,770). Full text copies were retrieved for 501 titles, which represented all titles identified as having potential relevance to our objectives or where there was insufficient information to make a decision as to relevance. A total of 108 articles (representing 97 original studies) comprised the final sample. Disagreements were resolved by consensus. When consensus could not be reached, a third senior member of the review team (CAE, LW) acted as an arbitrator and made the final decision (n = 9 articles). Figure 1 summarizes the results of the screening/selection process. A list of retrieved articles that were excluded can be found in Additional File 2.

### Data Extraction

Two reviewers (JES and HMO) performed data extraction: one reviewer extracted the data, which was then checked for accuracy by a second reviewer. We extracted data on: year of publication, study design, setting, sampling, subject characteristics, methods, the measure of research utilization used, substantive theory, measurement theory, responsiveness (the extent to which the measure can assess change over time), reliability (information on variances and standard deviations of measurement errors, item response theory test information functions, and reliability coefficients where extracted where it existed), reported statements of traditional validity (content validity, criterion validity, construct validity), and study findings reflective of the four sources of validity evidence (content, response processes, internal structure, and relations to other variables) outlined in the *Standards for Educational and Psychological Testing *(the *Standards*) [13]. *Content evidence *refers to the extent to which the items in a self-report measure adequately represent the content domain of the concept or construct of interest. *Response processes evidence *refers to how respondents interpret, process, and elaborate upon item content and whether this behaviour is in accordance with the concept or construct being measured. *Internal structure evidence *examines the relationships between the items on a self-report measure to evaluate its dimensionality. *Relations to other variables evidence *provide the fourth source of validity evidence. External variables may include measures of criteria that the concept or construct of interest is expected to predict, as well as relationships to other scales hypothesized to measure the same concepts or constructs, and variables measuring related or different concepts or constructs [13]. In the *Standards*, validity is a unitary construct in which multiple evidence sources contribute to construct validity. A higher number of validity sources indicate stronger construct validity. An overview of the *Standards *approach to reliability and validity assessment is in Additional File 3. All disagreements in data extraction were resolved by consensus.

There are no universal criteria to grade the quality of self-report measures. Therefore, in line with other recent measurement reviews [14,15], we did not use restrictive criteria to rate the quality of each study. Instead, we focused on performing a comprehensive assessment of the psychometric properties of the scores obtained using the research utilization measures reported in each study. In performing this assessment, we adhered to the *Standards*, considered best practice in the field of psychometrics [16]. Accordingly, we extracted data on all study results that could be grouped according to the *Standards' *critical reliability information and four validity evidence sources. To assess relations to other variables, we *a priori *(based on commonly used research utilization theories and systematic reviews) identified established relationships between research utilization and other (external) variables (See Additional File 3). The external variables included: individual characteristics (*e.g*., attitude towards research use), contextual characteristics (*e.g*., role), organizational characteristics (*e.g*., hospital size), and interventions (*e.g*., use of reminders). All relationships between research use and external variables in the final set of included articles were then interpreted as supporting or refuting validity evidence. The relationship was coded as 'supporting validity evidence' if it was in the same direction and had the significance predicted, and as 'refuting validity evidence' if it was in the opposite direction or did not have the significance predicted.

### Data Synthesis

The findings from the review are presented in narrative form. To synthesize the large volume of data extracted on validity, we developed a three-level hierarchy of self-report research utilization measures based on the number of validity sources reported in 50% or more of the studies for each measure. In the *Standards*, no one source of validity evidence is considered always superior to the other sources. Therefore, in our hierarchy, level one, two, and three measures provided evidence from any three, two, and one validity sources respectively. In the case of single-item measures, only three validity sources are applicable; internal structure validity evidence is not applicable as it assesses relationships between items. Therefore, a single-item measure within level one has evidence from all applicable validity sources.

## Results

### Objective 1: Identification and characteristics of self-report research utilization measures used in healthcare

In total, 60 unique self-report research utilization measures were identified. We grouped them into 10 classes as follows:

1. Nurses Practice Questionnaire (n = 1 Measure)

2. Research Utilization Survey (n = 1 Measure)

3. Edmonton Research Orientation Survey (n = 1 Measure)

4. Knott and Wildavsky Standards (n = 1 Measure)

5. Other Specific Practices Indices (n = 4 Measures) (See Additional File 4)

6. Other General Research Utilization Indices (n = 10 Measures) (See Additional File 4)

7. Past, Present, Future Use (n = 1 Measure)

8. Parahoo's Measure (n = 1 Measure)

9. Estabrooks' Kinds of Research Utilization (n = 1 Measure)

10. Other Single-Item Measures (n = 39 Measures)

Table 1 provides a description of each class of measures. Classes one through six contain multiple-item measures, while classes seven through ten contain single-item measures; similar proportions of articles reported multi- and single-item measures (n = 51 and n = 59 respectively, two articles reported both multi- and single-item measures). Only seven measures were assessed in multiple studies: Nurses Practice Questionnaire; Research Utilization Survey; Edmonton Research Orientation Survey; a Specific Practice Index [17,18]; Past, Present, Future Use; Parahoo's Measure; and Estabrooks' Kinds of Research Utilization. All study reports claimed to measure research utilization; however, 13 of the 60 measures identified were proxy measures of research utilization. That is, they measure variables related to using research (*e.g*., reading research articles) but not research utilization directly. The 13 proxy measures are: Nurses Practice Questionnaire, Research Utilization Questionnaire, Edmonton Research Orientation Survey, and the ten Other General Research Utilization Indices.

**Table 1:** Description of research utilization measure classes

Class	Description	No of Articles [Citations]
Nurses Practice Questionnaire (NPQ)	Developed for nurses. The NPQ consists of brief descriptions of 14 specific practice innovations. Seven questions measuring an individual's stage of innovation adoption are posed for each innovation. The first six questions measure adoption of the practice according to Roger's [91] Innovation-Decision Process Theory while the seventh question measures perception of policy existence with respect to the innovation. All items are scored dichotomously (yes/no) except for one item (on 'use'), which is scored as never, sometimes, or always.	11 articles [30-35,59-63]

Research Utilization Questionnaire (RUQ)	Developed for nurses. The RUQ consists of 42 self-descriptive statements comprising four subscales of which research use is one. The research use subscale contains 10 items, which measure the degree to which an individual feels they incorporate research findings into their daily practice. Items are scored on a 5-point Likert scale from strongly disagree to strongly agree.	16 articles [55,71,72,75,79,81,82,85,89,118-124]

Edmonton Research Orientation Survey (EROS)	Developed in the context of rehabilitation specialties (*e.g*., physiotherapy,). The EROS has four subscales of which the 'Using Research/Evidence-Based Practice' is one subscale. This subscale is composed of 10 items. Items are scored on a 5-point Likert scale from strongly disagree to strongly agree.	8 articles [37,76-78,125-128]

Knott and Wildavsky Standards	Developed for leaders based on Knott and Wildavsky's [93]*Standards of Research Use*. Consists of seven items to measure each of the seven standards of research use: reception, cognition, reference, effort, adoption, implementation, and impact. Items scored on a 5-point frequency scale from never to very often.	1 article [20]

Other Specific Practices Indices	Asks respondents to report on their use of a range of specific research-based practices. The number and kind of practices vary by the study. The scales used to measure use of the practices vary by study with some studies measuring use on frequency scales and others dichotomously as use or nonuse. (See Additional File 4)	5 articles [17,18,21,50,129]

Other General Research Use Indices	Each of these indices combines several items on respondents' general use of research (*i.e*., not use of specific practices) to derive an index (or overall score) representing their use of research. (See Additional File 4)	10 articles [24,36,50,73,84,101-105]

Past/Present/Future Use	Developed for nurses. Asks respondents to indicate their participation in one or more research activities in the past (> 6 months ago), present (most recent six months), and intention to use research in the future (within the next year). Responses are scored in a dichotomous yes/no format. Each item is considered individually, that is, items are not combined to form an index score.	3 articles [65,90,130]

Parahoo Measure	Developed for nurses. Measures research use with three single items. The three items are: frequency of use of research in clinical practice (scored on a 5-point frequency scale from never to all the time), implementation of new research findings in one's own practice in the last two years (scored dichotomously as yes/no), and to list up to three research findings that they have implemented in the last two years (open ended). Each item is considered individually, that is, items are not combined to form an index score.	7 articles [53,131-136]

Estabrooks' Kinds of Research Use	Developed for nurses. Measures research use with single items that tap four kinds of research use: instrumental (or direct), conceptual (or indirect), persuasive, and overall. Each item is preceded by a definition of the kind of research use and examples of that kind of research use. For each kind of research use, respondents are asked to indicate, over the past year, how often they have used research in this way. The items are treated individually (*i.e*., they are not combined to form an index). Items are scored on a 7-point (from never to nearly every shift) or 4-point (from never to nearly every work day) scale.	10 articles [3,26,66-70,74,80,86] (8 studies)

Other Single Item Measures	Developed for different types of healthcare professionals (depending on the target population of the study). Measures research use with a single-item developed for the study, and not used by others in subsequent studies. A variety of scoring methods are used depending on the study using different frequency scales, Likert agreement scales, dichotomous yes/no scales, and/or open-ended responses.	39 articles [22,23,25,27,28,37-49,51,52,54,57,58,83,87,88,137-149] (39 studies)

The majority (n = 54) of measures were assessed with healthcare providers. Professional nurses comprised the sample in 56 studies (58%), followed by allied healthcare professionals (n = 25 studies, 26%), physicians (n = 7 studies, 7%), and multiple clinical staff groups (n = 5 studies, 5%). A small proportion of studies (n = 5 studies, 5%) measured research utilization by healthcare decision makers. The decision makers, in each study, were members of senior management with direct responsibility for making decisions for a healthcare organization and included: medical officers and program directors [19]; managers in ministries and regional health authorities [20]; senior administrators [21]; hospital managers [22]; and executive directors [23]. A different self-report measure was used in each of these six studies. The unit/organization was the unit of analysis in 6 of the 97 (6%) included studies [22-27]; a unit-level score for research utilization was calculated by aggregating the mean scores of individual care providers.

**Figure 2:**
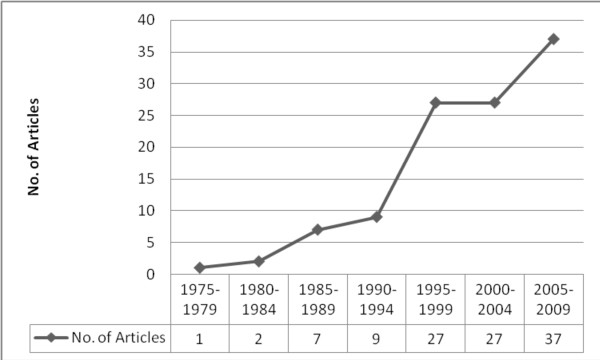
**Publication timeline**.

Most studies were conducted in North America (United States: n = 43, 44% and Canada: n = 22, 23%), followed by Europe (n = 22, 23%). Other geographic areas represented included: Australia (n = 5, 5%), Iran (n = 1, 1%), Africa (n = 2, 2%), and Taiwan (n = 2, 2%). With respect to date of publication, the first report included in this review was published in 1976 [28]. The majority of reports (n = 90, 83%) were published within the last 13 years (See Figure 2).

### Objective 2: Psychometric assessment of the self-report research utilization measures

Our psychometric assessment involved three components: acceptability, reliability, and validity.

### Acceptability

Acceptability in terms of time required to complete the research utilization measures and missing data (specific to the research utilization items) was not reported.

### Reliability

Reliability was reported in 32 (33%) of the studies (See Table 2 and Additional File 5). Internal consistency (Cronbach's Alpha) was the most commonly reported reliability statistic--it was reported for 13 of the 18 multi-item measures (n = 65, 67% of studies). Where reliability (Cronbach's Alpha) was reported, it almost always (n = 29 of 31 studies, 94%) exceeded the accepted standard (> 0.70) for scales intended to compare groups, as recommended by Nunnally and Bernstein [29]. The two exceptions were assessments of the Nurses Practice Questionnaire [30-32]. This tendency to only report reliability coefficients that exceed the accepted standard may potentially reflect a reporting bias.

**Table 2:** Summary of reported reliability of research utilization measures (N = 14 of 60 measures)

Class (No. of measures) [Citations]	Reliability		
	
	Internal consistency Range	Stability Range	Inter-rater
Nurses Practice Questionnaire (1) [30-35,59-63]	α = 0.63 to 0.95	r = 0.83 to 0.99	

Other Specific Practice Indices (1) [50]	α = 0.87		

Research Utilization Questionnaire (1) [55,71,79,81,82,85,118-120,123,124]	α = 0.79 to 0.94		

Edmonton Research Orientation Survey (1) [76-78,127]	α = 0.83 to 0.89		

Knott and Wildvasky Standards (1) [20]	α = 0.87		

Other General Research Utilization Indices (8) [24,36,50,73,84,101,103,105]	α = 0.73 to 0.94	r = 0.88	

Other Single Items (1) [37]	NA		r = 0.80 to 0.91

Stability, or test-retest, reliability was reported for only three (3%) of the studies: two studies assessing the Nurses Practice Questionnaire [33-35], and one study assessing Stiefel's Research Use Index [36]. All three studies reported Pearson *r *coefficients greater than 0.80 using one-week intervals (Table 2). One study also assessed inter-rater reliability. Pain *et al. *[37] had trained research staff and study respondents rate their (respondents) use of research on a 7-point scale. Inter-rater reliability among the interviewers was acceptable with pair wise correlations ranging from 0.80 to 0.91 (Table 2). No studies reported other critical reliability information consistent with the *Standards*, such as variances or standard deviations of measurement errors, item response theory test information functions, or parallel forms coefficients.

### Validity

No single research utilization measure had supporting validity evidence from all four evidence sources outlined in the *Standards*. For 12 measures [38-49], each in the 'other single-item' class, there were no reported findings that could be classified as validity evidence. The remaining 48 measures were classified as level one (n = 6), level two (n = 16), or level three (n = 26) measures, according to whether the average number of validity sources reported in 50% or more of the studies describing an assessment of the measure was three, two, or one, respectively. Level one measures displayed the highest number of validity sources and thus, the strongest construct validity. A summary of the hierarchy is presented in Tables 3, 4, and 5. More detailed validity data is located in Additional File 6.

**Table 3:** Level one research utilization measures (three validity sources), n = 6 measures

Class	Citation	Participants/Setting/Country	Validity			
			Content	Response processes	Internal structure	Relations
Other Specific Practices Indices*	[50]	Nurses/Hospitals/Canada	√	√		√

Other General Research Utilization Indices*	[24]	Nurses/Hospitals/USA	√	√		√
	
	[36]	Nurses/Hospitals/USA	√		√	√
	
	[50]	Nurses/Hospitals/Canada	√	√		√

Other Single Items*	[51]	Leaders/Community/Canada	√	√	NA	√
	
	[52]	Allied/Variety/Canada	√	√	NA	√

* = each study represents a unique measure

**Table 4:** Level two self-report research utilization measures (two validity sources), n = 16 measures

Class	Citation	Participants/Setting/Country	Validity			
			Content	Response processes	Internal structure	Relations
Nurses Practice Questionnaire	[33,34]	Nurses/Hospitals/USA	√			√
	
	[59]	Nurses/Education/USA	√			
	
	[32]	Nurses/Variety/Sweden	√			√
	
	[60]	Nurses/Hospitals/USA	√			√
	
	[61]	Nurses/Hospitals/USA	√			√
	
	[30,31]	Nurses/Hospitals/UK	√	√		√
	
	[62]	Nurses/Variety/USA	√			√
	
	[63]	Nurses/Hospitals/Canada	√			√
	
	[35]	Nurses/Hospitals/USA	√			

Knott and Wildvasky	[20]	Leaders/Variety/Canada	√			√

Other General Research Utilization Indices*	[101]	Nurses/Hospitals/USA			√	√
	
	[102]	Allied/Community/USA	√			√
	
	[105]	Allied/Variety/Sweden		√		√
	
	[104]	Allied/Not Reported/USA		√		√

Other Specific Practices Indices*	[21]	Leaders/Community/USA	√			√
	[17,18]	Multiple Staff/Hospitals/Africa		√		√

Estabrooks' Kinds of Research Use	[3,68]	Nurses/Variety/Canada	√	√	NA	√
	
	[74]	Nurses/Hospitals/USA			NA	√
	
	[69]	Nurses/Hospitals/USA, Canada			NA	√
	
	[26,70]	Nurses/Hospitals/Canada		√	NA	
	
	[66]	Allied/Variety/Canada		√	NA	
	
	[67]	Nurses/Long-term Care/USA		√	NA	√
	
	[80]	Nurses/Variety/Canada			NA	√
	
	[86]	Nurse Educators/Variety/Canada			NA	

Parahoo	[53,131-134]	Nurses/Hospitals/UK	√	√	NA	
	
	[135]	Nurses/Hospitals/Iran			NA	
	
	[136]	Nurses/Variety/UK	√	√	NA	

Other Single Items*	[139]	Allied/Variety/UK	√		NA	√
	
	[144]	Nurses/Hospitals/Nigeria	√		NA	√
	
	[83]	Nurses/Variety/USA	√	√	NA	
	
	[27]	Nurses/Hospitals/Canada		√	NA	√
	
	[88]	Nurses/Hospitals/USA	√	√	NA	
	
	[57]	Nurses/Hospitals/Taiwan	√	√	NA	

* = each study represents a unique measure

**Table 5:** Level three self-report research utilization measures (one validity source), n = 26 measures

Class	Citation	Participants/Setting/Country	Validity			
			Content	Response processes	Internal structure	Relations
Research Utilization Questionnaire	[55]	Nurses/Hospitals/USA	√			√
	
	[118,119]	Multiple Staff/Long-Term Care/Sweden				√
	
	[120]	Nurses/Long-Term Care/Sweden				√
	
	[71]	Multiple Staff/Variety/USA				√
	
	[72]	Nurses/Hospitals/Canada				√
	
	[121]	Nurses/Hospitals/UK				√
	
	[122]	Nurses/Hospitals/UK				
	
	[75]	Nurses/Hospitals/UK		√		
	
	[79,123]	Nurses/Hospitals/USA				√
	
	[81]	Nurses/Variety/USA				√
	
	[82]	Allied/Variety/Sweden				√
	
	[85]	Nurses/Hospitals/USA	√			√
	
	[124]	Nurses/Hospitals/Canada				√
	
	[89]	Nurses/Variety/Sweden				

Edmonton Research Orientation Survey	[125]	Allied/Hospitals/Canada	√			
	
	[126]	Nurses/Variety/Australia			√	√
	
	[127]	Multiple Staff/Hospitals/Australia				
	
	[77,78]	Nurses/Hospitals/Canada				√
	
	[76]	Allied/Hospitals/Canada				
	
	[37]	Allied/Variety/Canada		√		
	
	[128]	Allied/Variety/Canada				

Other General Research Utilization Indices*	[73]	Allied/Variety/Sweden				√
	
	[103]	Allied/Variety/USA				√
	
	[84]	Nurses/Hospitals/USA				√

Other Specific Practices Indices*	[129]	Allied/Variety/USA	√			

Past, Present, Future Use	[130]	Nurses/Variety/USA			NA	√
	
	[65]	Nurses/Hospitals/Canada			NA	√
	
	[90]	Nurses/Hospitals/USA			NA	√

Other Single Items*	[23]	Leaders/Community/Canada	√		NA	
	
	[137]	Physicians/Variety/Australia	√		NA	
	
	[138]	Allied/Variety/USA	√		NA	
	
	[140]	Nurses/Flight Team/USA			NA	√
	
	[28]	Allied/Variety/USA			NA	√
	
	[141]	Nurses/Variety/USA	√		NA	
	
	[22]	Leaders/Variety/USA		√	NA	
	
	[142]	Allied/Community/USA	√		NA	
	
	[25]	Allied/Hospitals/Europe		√	NA	
	
	[143]	Multiple Staff/Variety/USA			NA	√
	
	[145]	Physicians/Hospitals/Denmark			NA	√
	
	[37]	Allied/Variety/Canada		√	NA	
	
	[54]	Nurses/Hospitals/USA	√		NA	
	
	[87]	Allied/Variety/USA	√		NA	
	
	[146]	Allied/Variety/UK	√		NA	
	
	[58]	Nurses/Hospitals/Taiwan		√	NA	
	
	[147]	Nurses/Variety/UK		√	NA	
	
	[148]	Physicians/Variety/USA		√	NA	
	
	[149]	Nurses/Variety/Australia	√		NA	

* = each study represents a unique measure

### Measures reporting three sources of validity evidence (level one)

Six measures were grouped as level one: Specific Practices Indices (n = 1), General Research Utilization Indices (n = 3), and Other-Single Items (n = 2) (Table 3). Each measure was assessed in a single study. Five [24,50-52] of the six measures displayed content, response processes, and relations to other variables validity evidence, while the assessment of one measure [36] provided internal structure validity evidence. A detailed summary of the level one measures is located in Table 6.

**Table 6:** Level one summary

Class [Citation]	Research Utilization Measure Details	Sample and Setting	Validity Assessment	
			**Supporting Evidence**	**Comments**

Specific Practice Indices [50]	Use of 10 specific research practices. Sample practices include: • IM injection • Catheter removal • Sensory information/diagnostic Each practice was scored on a 3 pt scale: never (1), sometimes (2), always (3) or 'not applicable.' A mean score based on the ten practices was then calculated.	Population: Nurses Country: Canada Setting: Hospitals	Content: Measure assessed by an expert panel Response processes: a pilot test was conducted within a larger survey (of which the research utilization index was one component). Relations to other variables: correlations with other variables were reported that support theory and prior empirical research (*e.g*., with supportive climate and infrastructure)	Content: Unknown whether content assessment was on the specific research-based practices, the question pertaining to use that followed each practice, or both. A high quality assessment of content evidence should include both.

General Research Utilization Indices [50]	Research use index contains 10 general statements on research use. Sample items include: • Communicating concerns about the effectiveness of practices to colleagues • Use of research articles to support questioning practice • Identification of hospital policies based on research Each item is scored on a 4-point scale from not at all to always. Item scores are then summed for an index score (10 to 40).	Population: Nurses Country: Canada Setting: Hospitals	Content: Measure assessed by a peer panel Response processes: a pilot test was conducted with the larger survey (of which the research utilization index was one component). Relations to other variables: Non-significant correlations (as predicted) with other variables (education and valuing research) which support past empirical reviews.	Content: Process or findings of the content assessment not reported.

General Research Utilization Indices [24]	Research use index consists of five items focusing on the extent to which respondents participate in research activities. Sample items include: • Reviewed research literature in an effort to identify new knowledge for use in your practice • Evaluated a research study to determine its value for practice Each item is asked with respect to the past year and is scored on a 4-point scale: 0, 1, 2-4, 5 or more times. Mean of the items are then taken as a measure of research utilization.	Population: Nurses Country: USA Setting: Hospitals	Content: Development of the research utilization index was based on a set of five rules (See Additional File 4). Response processes: a small pretest was conducted with the larger survey (of which the research utilization index was one component). Relations to other variables: Covariance analysis reported. Several variables were shown to be nonsignificant as predicted, for example, professionalism.	

General Research Utilization Indices [36]	Research use index consists of 18 items measuring respondents' reported participation in nursing research utilization activities. Sample items include: • I read nursing research articles and learn about research-based nursing interventions. • I attend conferences/educational programs and learn about research-based nursing interventions Each item is scored on a 5-point scale from never to always. Item scores are then summed for an index score (18 to 90).	Population: Nurses Country: USA Setting: Hospitals	Content: A panel of four experts on research use by nurses assessed the index. Reasons for selecting each panel member were reported, illustrating the appropriateness of the panel selection. Internal structure: Factor analysis was conducted; findings revealed a 3-factor solution. Relations to other variables: A significant association between specialty (working in critical care settings) and research use was reported (as predicted).	Content: Findings from the content assessment were not reported. Internal structure: The 18 items were combined to compute one derived research utilization score (but factor analysis revealed three factors and thus supported deriving three scores and not one score).

Other Single-Items [51]	Five single items asking respondents (decision-makers) whether they have used five specific systematic reviews in the past two years to make a program-related decision. All five items are scored as yes or no. Each item is analyzed as an individual item.	Population: Decision-Makers Country: Canada Setting: Community	Content: The research utilization item, which was a component of a larger survey, was developed based on a review of research utilization literature, suggesting content validity evidence. Response processes: a pilot test was conducted with the larger survey (of which the research utilization item was one component). Relations to other variables: correlations with other variables, for example, perception that the systematic reviews are easy to use.	All applicable sources of validity evidence reported

Other Single-Items [52]	A single item asking respondents whether they have applied research to their practice. Scored on a 4-point Likert scale: never, rarely, sometimes, always	Population: Allied Health Professionals Country: Canada Setting: Variety of settings	Content: An expert panel assessed the research utilization item, which was a component of a larger survey. Response processes: A pilot test was conducted with the larger survey (of which the research utilization item was one component). Relations to other variables: a significant association with attitude towards research (as predicted).	All applicable sources of validity evidence reported. Content: The composition of the panel, process undertaken, or related findings were not reported.

### Measures reporting two sources of validity evidence (level two)

Sixteen measures were grouped as level two: Nurses Practice Questionnaire (n = 1); Knott and Wildvasky Standards (n = 1); General Research Utilization Indices (n = 4); Specific Practices Indices (n = 2); Estabrooks' Kinds of Research Utilization (n = 1); Past, Present, Future Use (n = 1); and Other Single-Items (n = 6) (Table 4). Most assessments occurred with nurses in hospitals. No single validity source was reported for all level two measures. For the 16 measures in level two, the most commonly reported evidence source was relations to other variables (reported for 12 [75%] of the measures), followed by response processes (n = 7 [44%] of the measures), content (n = 6 [38%] of the measures), and lastly, internal structure (n = 1 [6%] of the measures). Four of the measures were assessed in multiple studies: Nurses Practice Questionnaire, a Specific Practices Index [17,18], Parahoo's Measure, and Estabrooks' Kinds of Research Utilization.

### Measures reporting one source of validity evidence (level three)

The majority (n = 26) of research utilization measures identified fell into level three: Champion and Leach's Research Utilization Survey (n = 1); Edmonton Research Orientation Survey (n = 1); General Research Utilization Indices (n = 3); Specific Practices Indices (n = 1); Past, Present, Future Use (n = 1); and, Other Single-Item Measures (n = 19) (Table 5). The majority of level three measures are single-items (n = 20) and have been assessed in a single study (n = 23). Similar to level two, there was no single source of validity evidence common across all of the level three measures. The most commonly reported validity source was content (reported for 12 [46%] of the measures), followed by response processes (n = 10, 38%), relations to other variables (n = 10, 38%), and lastly, internal structure evidence (n = 1, 4%). Three level three measures were assessed in multiple studies: the Research Utilization Questionnaire; Past, Present, Future Use items; and the Edmonton Research Orientation Survey.

### Additional properties

As part of our validity assessment, we paid special attention to how each measure 'functioned'. That is, 'were the measures behaving as they should'. All six level one measures and the majority of level two measures (n = 12 of 16) displayed 'relations to other (external) variables' evidence, indicating that the measures are functioning as the literature hypothesizes a research utilization measure should function. Fewer measures in level three (n = 10 of 26) displayed optimal functioning (Table 5 and Additional File 5). We also looked for evidence of responsiveness of the measures (the extent to which the measure captures change over time); no evidence was reported.

## Discussion

Our discussion is organized around three areas: the state of the science of research utilization measurement, construct validity, and our proposed hierarchy of measures.

### State of the science

In 2003, Estabrooks *et al. *[12] completed a review of self-report research utilization measures. By significantly extending the search criteria of that review, we identified 42 additional self-report research utilization measures, a substantial increase in the number of measures available. While, on the surface, this gives the impression of an optimistic picture of research utilization measurement, detailed inspection of the 108 articles included in our review revealed several limitations to these measures. These limitations seriously constrain our ability to validly measure research utilization. The limitations center on ambiguity between different measures and between studies using the same measure, and methodological problems with the design and evaluation of the measures.

### Ambiguity in self-report research utilization measures

There is ambiguity with respect to the naming of self-report research utilization measures. For example, similar measures have different names. Parahoo's Measure [53] and Pettengil's single item [54], for example, both ask participants one question--whether they have used research findings in their practice in the past two years or three years, respectively. Conversely, other measures that ask substantially different questions are similarly named; for example, Champion and Leach [55], Linde [56], and Tsai [57,58] all describe a Research Utilization Questionnaire. Further ambiguity was seen in the articles that described the modification of a pre-existing research utilization measure. In most cases, despite making significant modifications to the measure, the authors retained the original measure's name and, thus, masked the need for additional validity testing. The Nurses Practice Questionnaire is an example of this. Brett [33] originally developed the Nurses Practice Questionnaire, which consisted of 14 research-based practices, to assess research utilization by hospital nurses. The Nurses Practice Questionnaire was subsequently modified (the number of and actual practices assessed, as well as the items that follow each of the practices) and used in eight additional studies [30-32,35,59-63], but each study retained the Nurses Practice Questionnaire name.

### Methodological problems

In the earlier research utilization measurement review, Estabrooks *et al. *[12] identified four core methodological problems, lack of: construct clarity, use of research utilization theory, use of measurement theory, and psychometric assessment. In our review, we found that, despite an additional 10 years of research, 42 new measures and 65 new reports of self-report research utilization measures, these problems and others persist.

### Lack of construct clarity

Research utilization has been, and is likely to remain, a complex and contested construct. Issues around clarity of research utilization measurement stems from four areas: a lack of definitional precision of research utilization, confusion around the formal structure of research utilization, lack of substantive theory to develop and evaluate research utilization measures, and confusion between factors associated with research utilization and the use of research *per se*.

Lack of definitional precision with respect to research utilization is well documented. In 1991, knowledge utilization scholar Thomas Backer [64] declared lack of definitional precision as part of a serious challenge of fragmentation that was facing researchers in the knowledge (utilization) field. Since then, there have been substantial efforts to understand what does and does not make research utilization happen. However, the issue of definitional precision continues to be largely ignored. In our review, definitions of research utilization were infrequently reported in the articles (n = 36 studies, 37%) [3,20,23,30,32,36,37,40,51,53,57,63,65-90] and even less frequently incorporated into the administered measures (n = 8 studies, 8%) [3,67-70,74,80,86,88]. Where definitions of research utilization were offered, they varied significantly between studies (even studies of the same measure) with one exception: Estabrooks' Kinds of Research Utilization. In this latter measure, the definitions offered were consistent in both the study reports and the administered measure.

A second reason for the lack of clarity in research utilization measurement is confusion around the formal structure of research utilization. The literature is characterized by multiple conceptualizations of research utilization. These conceptualizations influence how we define research utilization and, consequently, how we measure the construct and interpret the scores obtained from such measurement. Two prevailing conceptualizations dominating the field are research utilization as process (*i.e*., consists of a series of stages/steps) and research utilization as variable or discrete event (a 'variance' approach). Despite debate in the literature with respect to these two conceptualizations, this review revealed that the vast majority of measures that quantify research utilization do so using a 'variable' approach. Only two measures were identified that assess research utilization using a 'process' conceptualization: Nurses Practice Questionnaire [33] (which is based on Rogers' *Innovation Decision Process *Theory [91,92]) and Knott and Wildavsky's Standards measure (developed by Belkhodja *et al. *and based on Knott and Wildavsky's *Standards of Research Use *model [93]). Some scholars also prescribe research utilization as typological in addition to being a variable or a process. For example, Stetler [88] and Estabrooks [3,26,66-70,74,80,86] both have single items that measure multiple kinds of research utilization, with each kind individually conceptualized as a variable. Grimshaw *et al. *[8], in a systematic review of guideline dissemination and implementation strategies, reported a similar finding with respect to limited construct clarity in the measurement of guideline adherence in healthcare professionals. Measurement of intervention uptake, they argued, is problematic because measures are mostly around the 'process' of uptake rather than on the 'outcomes' of uptake. While both reviews point to lack of construct clarity with respect to process versus variable/outcome measures, they report converse findings with respect to the dominant conceptualization in existing measures. This finding suggests a comprehensive review targeting the psychometric properties of self-report measures used in guideline adherence is also needed. While each conceptualization (process, variable, typological) of research utilization is valid, there is, to date, no consensus regarding which one is best or the most valid.

A third reason for the lack of clarity in research utilization measurement is limited use of substantive theory in the development of research utilization measures. There are numerous theories, frameworks, and models of research utilization and of related constructs, from the fields of nursing (*e.g*., [94-96]), organizational behaviour (*e.g*., [97-99]), and the social sciences (*e.g*., [100]). However, only 1 of the 60 measures identified in this review explicitly reported using research utilization theory in its development. The Nurses Practice Questionnaire [33] was developed based of Rogers' Innovation-Decision Process theory (one component of Rogers' larger Diffusion of Innovations theory [91]). The Innovation-Decision Process theory describes five stages to the adoption of an innovation (research): awareness, persuasion, decision, implementation, and confirmation. A similar finding regarding limited use of substantive theory was also reported by Grimshaw *et al. *[8] in their review of guideline dissemination and implementation strategies. This limited use of theory in the development and testing of self-report measures may therefore reflect the more general state of the science in the research utilization and related (*e.g*., knowledge translation) fields that requires addressing.

A fourth and final reason that we identified for the lack of clarity in research utilization measurement is confusion between factors associated with research utilization and the use of research *per se*. The Nurses Practice Questionnaire [33] and all 10 Other General Research Utilization Indices ([24,36,50,73,84,101-105]) claim to directly measure research utilization. However, their items, which while compatible with a process view of research utilization, do not directly measure research utilization. For example, 'reading research' is an individual factor that fits into the awareness stage of Rogers' Innovation Decision-Process theory. The Nurses Practice Questionnaire uses this item to create an overall 'adoption' score, which is interpreted as 'research use', but it is not 'use'. A majority of the General Research Utilization Indices also includes reading research as an item. In these measures, such individual factors are treated as proxies for research utilization. We caution researchers that while many individual factors like 'reading research' may be a desirable quality for making research utilization happen, they are not research utilization. Therefore, when selecting a research utilization measure to use, the aim of the investigation is paramount; if the aim is to examine research utilization as an event, then measures that incorporate proxies should be avoided.

### Lack of measurement theory

Foundational to the development of any measure is measurement theory. The two most commonly used measurement theories are classical test score theory, and modern measurement (or item response) theory. Classical test score theory proposes that an individual's observed score on a construct is the additive composite of their true score and random error. This theory forms the basis for traditional reliability theory (Cronbach's Alpha) [106,107]. Item response theory is a model-based theory that relates the probability of an individual's response to an item on an underlying trait. It proposes that as an individual's level of a trait (research utilization) increases, the probability of a correct (or in the case of research utilization, a more positive) response also increases [108,109].

Similar to the previous review by Estabrooks *et al. *[12], none of the reports in our review explicitly stated that consideration of any kind was given to measurement theory in either the development or assessment of the respective measures. However, in our review, for 14 (23%) of the measures, there was reliability evidence consistent with the adoption of a classical test score theory approach. For example: Cronbach's alpha coefficients were reported on 13 (22%) measures (Table 2) and principal components (factor) analysis and item total correlations were reported on 2 (3%) measures (Tables 3 and 4).

### Lack of psychometric assessment

In the previous review, Estabrooks *et al. *[12] concluded, 'All of the current studies lack significant psychometric assessment of used instruments.' They further stated that over half of the studies in their review did not mention validity, and that only two measures displayed construct validity. This latter finding, we argue, may be attributed to the adoption of a traditional conceptualization of validity where only evidence labeled as validity by the original study authors were considered. In our review, a more positive picture was displayed, with only 12 (20%) of the self-report research utilization measures identified showing no evidence of construct validity. We attribute this, in part, to our implementation of the *Standards *as a framework for validity. Using this framework, we scrutinized all results (not just those labeled as validity), in terms of whether or not they added to overall construct validity.

### Additional limitations to the field

Several additional limitations in research utilization measurement were also noted as a result of this review. They include: limited reporting of data reflective of reliability beyond standard internal consistency (Cronbach's Alpha) coefficients; limited reporting of study findings reflective of validity; limited assessments of the same measure in multiple (> 1) studies; lack of assessment of acceptability and responsiveness; overreliance on the assessment made in the index (original) study of a measure; and failure to re-establish validity when modifications are made and/or the measure is assessed in a new population or context.

### Construct validity (the standards)

Traditionally, validity has been conceptualized according to three distinct types: content, criterion, and construct. While this way of thinking about validity has been useful, it has also caused problems. For example, it has led to compartmentalized thinking about validity, making it 'easier' to overlook the fact that construct validity is really the whole of validity theory. It has also led to the incorrect view of validity as a property of a measure rather than of the scores (and resulting interpretations) obtained with the measure. A more contemporary conceptualization of validity (seen in the *Standards*) was taken in this review. Using this approach, validity was conceptualized as a unitary concept with multiple sources of evidence, each contributing to overall (construct) validity [13]. We believe this conceptualization is both more relevant and more applicable to the study of research utilization than is the traditional conceptualization that dominates the literature [16,110].

All self-report measures require validity assessments. Without such assessments little to no intrinsic value can be placed on findings obtained with the measure. Validity is associated with the interpretations assigned to the scores obtained using a measure, and thus is intended to be hypothesis-based [110,111]. Hence, to establish validity, desired score interpretations are first hypothesized to allow for the deliberate collection of data to support or refute the hypotheses [112]. In line with this thinking, data collected using a research utilization self-report measure will always be more or less valid depending on the purpose of the assessment, the population and setting, and timing of the assessment (*e.g*., before or after an intervention). As a result, we are not able to declare any of the measures we identified in our review as valid or invalid, but only as more or less valid for selected populations, settings, and situations. This deviates substantially from traditional thinking, which suggests that validity either exists or not.

According to Cronbach and Meehl [113], construct validity rests in a nomological network that generates testable propositions that relate scores obtained with self-report measures (as representations of a construct) to other constructs, in order to better understand the nature of the construct being measured [113]. This view is comparable to the traditional conceptualization of construct validity as existing or not, and is also in line with the views of philosophers of science from the first half of the 20th century (*e.g*., Duhem [114] and Lakatos [115]). Duhem and Lakatos both contended that any theory could be fully justified or falsified based on empirical evidence (*i.e*., based on data collected with an specific measure). From this perspective, construct validity exists or not. In the second half of the 20th century, however, movement away from justification to what was described by Feyerabend [116] and Kuhn [117] as 'nonjustificationism' occurred. In nonjustificationism, a theory is never fully justified or falsified. Instead, at any given time, it is a closer or further approximation of the truth than another (competing) theory. From this perspective, construct validity is a matter of degree (*i.e*., more or less valid) and can change with the sample, setting, and situation being assessed. This is in line with a more contemporary (the *Standards*) conceptualization of validity.

### Self-report research utilization measure hierarchy

The *Standards *[13] provided us with a framework to create a hierarchy of research utilization measures and thus, synthesize a large volume of psychometric data. In an attempt to display the overall extent of construct validity of the measures identified, our hierarchy (consistent with the *Standards*) placed equal weight on all four evidential sources. While we were able to categorize 48 of the 60 self-report research utilization measures identified into the hierarchy, several cautions exist with respect to use of the hierarchy. First, the levels in the hierarchy are based on the number of validity sources reported, and not on the actual source or quality of evidence within each source. Second, some measures in our hierarchy may appear to have strong validity only because they have been subjected to limited testing. For example, the six measures in level one have only been tested in a single study. Third, the hierarchy included all 48 measures that displayed any validity evidence. Some of these measures, however, are proxies of research utilization. Overall, the hierarchy is intended to present an overview of validity testing to date on the research utilization measures identified. It is meant to inform researchers regarding what testing has been done and, importantly, where additional testing is needed.

## Limitations

Although rigorous and comprehensive methods were used for this review, there are three study limitations. First, while we reviewed dissertation databases, we did not search all grey literature sources. Second, due to limited reporting of findings consistent with the four sources of validity evidence in the *Standards*, we may have concluded lower levels of validity for some measures than actually exist. In the latter case, our findings may reflect poor reporting rather than less validity. Third, our decision to exclude articles that reported on healthcare providers' adherence to clinical practice guidelines may be responsible for the limited number of articles sampling physicians included in the review. A systematic review conducted by Grimshaw *et al. *[8] on clinical practice guidelines reported physicians alone were the target of 174 (74%) of the 235 studies included in that review. A future review examining the psychometric properties of self-report measures used to quantify guideline adherence would therefore be a fruitful avenue of inquiry.

## Conclusions

In this review, we identified 60 unique self-report research utilization measures used in healthcare. While this appears to be a large and definite set of measures, our assessment paints a rather discouraging picture of research utilization measurement. Several of the measures, while labeled research utilization measures, did not assess research utilization *per se*. Substantial methodological advances in the research utilization field, focusing in the area of measurement (in particular with respect to construct clarity, use of measurement theory, and psychometric assessment) are urgently needed. These advances are foundational to ensuring the availability of defensible self-report measures of research utilization. Also needed are improved reporting practices and the adoption of a more contemporary view of validity (the *Standards*) in future research utilization measurement studies.

## Competing interests

The authors declare that they have no competing interests.

## Authors' contributions

JES, CAE, PG, and LW participated in designing the study and securing funding for the project. JES, CAE, HMO, PG, and LW participated in developing the search strategy, study relevance, and data extraction tools. JES and HMO undertook the article selection and data extraction. All authors participated in data synthesis. JES drafted the manuscript. All authors provided critical commentary on the manuscript and approved the final version.

## Supplementary Material

Additional file 1 **Search Strategy**. This file contains the details of the search strategy used for the review. Click here for file

Additional file 2 **Exclusion List by Reason (N = 393)**. This file contains a list of the retrieved articles that were excluded from the review and the reason each article was excluded. Click here for file

Additional file 3 **The Standards**. This file contains an overview of the *Standards for Educational and Psychological Testing *Validity Framework and sample predictions used to assess 'relations to other variables' validity evidence according to this framework. Click here for file

Additional file 4 **Description of Other Specific Practices Indices and Other General Research Use Indices**. This file contains a description of the four measures included in the class 'Other Specific Practices Indices' and the ten measures included in the class 'Other General Research Use Indices'. Click here for file

Additional file 5 **Reported Reliability of Self-Report Research Utilization Measures**. This file contains the reliability coefficients reported in the included studies. Click here for file

Additional file 6 **Supporting Validity Evidence by Self-Report Research Utilization Measure**. This file contains the detailed validity evidence on each included self-report research utilization measure. Click here for file
