# Supplementary material for: A systematic review of the psychometric properties of self-report research utilization measures used in healthcare
Source: Implement Sci. 2011 Jul 27;6:83. doi: 10.1186/1748-5908-6-83 (PMC3169486; doi:10.1186/1748-5908-6-83)
Supplement: Additional file 1 — Search Strategy. This file contains the details of the search strategy used for the review. [file 1748-5908-6-83-S1.PDF]

Additional File 1. *Search Strategy*

Data Base	Edition	Search Terms	No. Articles
Cochrane Database of Systematic Reviews (CDSR)	Through to 4th quarter, 2008	"nursing practice questionnaire" or "edmonton research orientation survey" or (technolog* W diffus*) or (chnolog* W transfer*) or (technolog* W translat*) or (technolog* W adopt*) or "diffusion of innovation" or "diffusion of innovations" or "innovation diffusion" or "dissemination of innovation" or "dissemination of innovations" or (innovation* W/1 adopt*) or "adoption of innovation" or "adoption of innovations" or "dissemination of evidence" or "implementation of evidence" or "adoption of evidence" or "uptake of evidence" or "use of evidence" or "utilization of evidence" or "utilisation of evidence" or "diffusion of evidence" or "translation of knowledge" or "transfer of knowledge" or "implementation of knowledge" or "adoption of knowledge" or "uptake of knowledge" or "utilization of knowledge" or "utilisation of knowledge" or "dissemination of knowledge" or "diffusion of knowledge" or "implementation of technologies" or "adoption of technologies" or "uptake of technologies" or "dissemination of technologies" or "diffusion of technologies" or "translation of technologies" or "transfer of technologies" or "implementation of technology" or "adoption of technology" or "uptake of technology" or "dissemination of technology" or "diffusion of technology" or "translation of technology" or "transfer of technology" or "translation of research" or "transfer of research" or "implementation of research" or "adoption of research" or "uptake of research" or "use of research" or "utilization of research" or "utilisation of research" or "dissemination of research" or "diffusion of research" or "evidence uptake" or "evidence use" or "evidence diffusion" or "evidence dissemination" or "evidence utilization" or "evidence utilisation" or "evidence transfer" or "evidence translation" or "evidence implementation" or "evidence adoption" or "knowledge uptake" or "knowledge use" or "knowledge diffusion"or "knowledge dissemination" or "knowledge utilization" or "knowledge utilisation" or "knowledge transfer" or "knowledge translation" or "knowledge implementation" or "knowledge adoption" or "research uptake" or "research use" or "research diffusion" or "research dissemination" or "research utilization" or "research utilisation" or "research transfer" or "research translation" or "research implementation" or "research adoption" **AND** survey* or questionnaire* or inventor* or instrument* or scale* or assess* or evaluat* or measur* or tool* or reliability or validity or validation or reproducib* or benchmark* or psychometric*	0
Health and Psychosocial Instruments (HAPI)	Through to October 14, 2008		74
MEDLINE	Through to October 11, 2008		7064
CINAHL	Through to October 11, 2008		4939
EMBASE	Through to October 13, 2008		4684
Web of Science	Through to October 13, 2008		6692
SCOPUS	Through to November 17, 2008		8080
OCLC Papers First	Through to October 13, 2008		806
OCLC WorldCat	Through to October 13, 2008		2012
Sociological Abstracts	Through to October 13, 2008		2266
Proquest: Dissertation Abstracts, ABI Inform	Through to October 14, 2008		6135
Manual Search			18
TOTAL			42,770
