# Supplementary material for: A systematic review of the psychometric properties of self-report research utilization measures used in healthcare
Source: Implement Sci. 2011 Jul 27;6:83. doi: 10.1186/1748-5908-6-83 (PMC3169486; doi:10.1186/1748-5908-6-83)
Supplement: Additional file 2 — Exclusion List by Reason (N = 393). This file contains a list of the retrieved articles that were excluded from the review and the reason each article was excluded. [file 1748-5908-6-83-S2.PDF]

Additional File 2. ***Exclusion List by Reason (N=393)*** (organized by exclusion reason)

Citation	Exclusion reason
Steenrod, S. A. (2004). The use of evidence-based practices in substance abuse treatment programs. Journal of Evidence-Based Social Work, 1(4), 33-51.	Guideline adherence
Crane, J. (1989). Factors associated with the use of research-based knowledge in nursing (Dissertation)	Guideline adherence (use of CURN protocols)
Kreuger J. C. (1982). A survey of research utilization in community health nursing: Using research in practice. Western Journal of Nursing Research, 4, 244-248.	Guideline adherence (use of CURN protocols)
LoftusHills, A., & Duff, L. (1997). Implementation of nutrition standards for older adults. Nursing Standard, 11(44), 33-37.	Guideline adherence
Alerany, C., Campany, D., Monterde, J., & Semeraro, C. (2005). Impact of local guidelines and an integrated dispensing system on antibiotic prophylaxis quality in a surgical centre. Journal of Hospital Infection, 60(2), 111-117.	Guideline adherence
Bradley, et al. (2006). Implementation of evidence-based alcohol screening in the VHA. The American Journal of Managed Care, 12(10), 597-606.	Guideline adherence
Brehaut, J. C., Stiell, I. G., & Graham, I. D. (2006). Will a new clinical decision research rule be widely used? The case of the Canadian c-spine research rule. Academic Emergency Medicine, 13(4), 413-420.	Guideline adherence
Burkoski, V. (2002). Infant sleep position: Nurses' awareness and practice of the Canadian joint statement recommendation. (University of Windsor, Canada).	Guideline adherence
Crawford, V. L., McPeake, B., & Stout, R. W. (1995). Diagnostic regimes for urinary tract infection: Are research results applied to practice? Ulster Medical Journal, 64(2), 131-136.	Guideline adherence
Delnevo, C. D. et al. (2000). Injury-prevention counseling among residents of internal medicine. American Journal of Preventive Medicine, 19(1), 63-65.	Guideline adherence
Friedman, J.R. (1984). The acceptance of national dietary recommendations by nutrition educators as related to their use of professional sources of information and to their professional and personal characteristics (New York University).	Guideline adherence
Graham, I. D., et al. (2001). Awareness and use of the Ottawa Ankle and Knee Research Rules in 5 countries: Can publication alone be enough to change practice? Annals of Emergency Medicine, 37(3), 259-266.	Guideline adherence
Hill, M.N (1986). Diffusion of 1984 hypertension consensus recommendations among clinicians. Maryland, the John Hopkins University.	Guideline adherence
Johnson, F. E., & Maikler, V. E. (2001). Nurses' adoption of the AWHONN/NANN neonatal skin care project. Newborn and Infant Nursing Reviews, 1(1), 59-67.	Guideline adherence

Citation	Exclusion reason
Kinsman, L., & James, E. L. (2001). Evidence-based practice needs evidence-based implementation. Lippincott's Case Management, 6(5), 208-216.	Guideline adherence
Knaus, et al. (1996). Innovations in continuing education. We audit our own charts, thank you! Journal of Continuing Education in the Health Professions, 16(2), 117-124.	Guideline adherence
Kothari, A., Birch, S., & Charles, C. (2005). "Interaction" and research utilisation in health policies and programs: Does it work? Health Policy, 71(1), 117-125.	Guideline adherence
Lia-Hoagberg, B., Schaffer, M., & Strohschein, S. (1999). Public health nursing practice guidelines: An evaluation of dissemination and use. Public Health Nursing, 16(6), 397-404.	Guideline adherence
Manes, G., et al. (2001). Diffusion of knowledge about Helicobacter pylori as assessed in an open-access endoscopy system: A prospective observational study based on the Maastricht guidelines. Digestive Diseases, 19(2), 158-163.	Guideline adherence
McGlynn, E. A., et al. (2003). The quality of health care delivered to adults in the United States. The New England Journal of Medicine, 348(26), 2635-2645.	Guideline adherence
McPhee, S. J., & Bird, J. A. (1990). Implementation of cancer prevention guidelines in clinical practice. Journal of General Internal Medicine, 5(5 Suppl), S116-22.	Guideline adherence
Mehta, R. R. (2002). The implementation of new treatment guidelines in asthma in a social system by health care providers: An application of the diffusion theory. Idaho State University (Thesis).	Guideline adherence
Mesters, I., & Meertens, R. M. (1999). Monitoring the dissemination of an educational protocol on pediatric asthma in family practice: A test of associations between dissemination variables. Health Education & Behavior, 26(1), 103-120.	Guideline adherence
Miller, E. H., et al. (1999). Institution-wide pain management improvement through the use of evidence-based content, strategies, resources, and outcomes. Quality Management in Healthcare, 7(2), 28-40.	Guideline adherence
Montgomery, L. A., Hanrahan, K., Kottman, K., Otto, A., Barrett, T., & Hermiston, B. (1999). Guideline for I.V. infiltrations in pediatric patients. Pediatric Nursing, 25(2), 167-169.	Guideline adherence
O'Connor, et al. (1999) Geographic variation in the treatment of acute myocardial infarction. JAMA, 281(7), 627-633.	Guideline adherence
Pratt, C. N. U., Paone, D., Carter, R. J., & Layton, M. C. (2002). Hepatitis C screening and management practices: A survey of research use treatment and syringe exchange programs in New York City. American Journal of Public Health, 92(8), 1254-1256.	Guideline adherence

Citation	Exclusion reason
Rahimian, A., Driscoll, M., & Taylor, D. (1998). The maternal and child health sites' practices regarding HIV education, counseling, and testing of women of reproductive age in Chicago: Barriers to universal implementation. Maternal and Child Health Journal, 2(1), 35-44.	Guideline adherence
Redfern, et al. 2000. Evaluation of change in practice: South Thames Evidence-based Practice Project (STEP). Executive summary.<br><br>***Referenced by Titler 2004***	Guideline adherence
Ring, N., Coull, A., Howie, C., Murphy-Black, T., & Watterson, A. (2006). Analysis of the impact of a national initiative to promote evidence-based nursing practice. International Journal of Nursing Practice, 12(4), 232-240.	Guideline adherence
Ring, N., Malcolm, C., Coull, A., Murphy-Black, T., & Watterson, A. (2005). Nursing best practice statements: An exploration of their implementation in clinical practice. Journal of Clinical Nursing, 14(9), 1048-1058.	Guideline adherence
Roghmann, M., Perdue, B. E., & Polish, L. (1999). Concise communications. Vancomycin use in a hospital with vancomycin restriction. Infection Control and Hospital Epidemiology, 20(1), 60-63.	Guideline adherence
Roila, F. (2004). Transferring scientific evidence to oncological practice: A trial on the impact of three different implementation strategies on antiemetic prescriptions. Supportive Care in Cancer, 12(6), 446-453.	Guideline adherence
Rosenthal, V. D., Guzman, S., & Safdar, N. (2004). Effect of education and performance feedback on rates of catheter-associated urinary tract infection in intensive care units in Argentina. Infection Control and Hospital Epidemiology, 25(1), 47-50.	Guideline adherence
Rof, J., Mittendorf, T., Pirk, O., & Graf Von Der Schulenburg, J.M. (2002). Diffusion of innovations: Treatment of Alzheimer's disease in Germany. Health Policy, 60(1), 59-66.	Guideline adherence
Shah, S. S., Sinkowitz-Cochran, R. L., Keyserling, H. L., & Jarvis, W. R. (1999). Vancomycin use in pediatric neurosurgery patients. American Journal of Infection Control, 27(6), 482-487.	Guideline adherence
Sheehan, A. K., Walrath, C. M., & Holden, E. W. (2007). Evidence-based practice use, training and implementation in the community-based service setting: A survey of children's mental health service providers. Journal of Child and Family Studies, *16*(2), 169-182.	Guideline adherence
Specht, J. P., Bergquist, S., & Frantz, R. A. (1995). Adoption of a research-based practice for treatment of pressure ulcers. Nursing Clinics of North America, 30(3), 553.	Guideline adherence
Sproat, L. J., & Inglis, T. J. (1994). A multicentre survey of hand hygiene practice in intensive care units. Journal of Hospital Infection, 26(2), 137-148.	Guideline adherence

Citation	Exclusion reason
Valenstein, M., McCarthy, J. F., Austin, K. L., Greden, J. F., Young, E. A., & Blow, F. C. (2006). What happened to lithium? Antidepressant augmentation in clinical settings. American Journal of Psychiatry, 163(7), 1219-1225.	Guideline adherence
Valente, S. M. (2005). Evaluation of innovative research-based fact sheets. Journal for Nurses in Staff Development, 21(4), 171-176.	Guideline adherence
Wakefield, B., Johnson, J. A., Kron-Chalupa, J., & Paulsen, L. (1998). A research-based guideline for appropriate use of transdermal fentanyl to treat chronic pain. Oncology Nursing Forum, 25(9), 1505-1513.	Guideline adherence
Weilburg, J. B., O'Leary, K. M., Meigs, J. B., Hennen, J., & Stafford, R. S. (2003). Evaluation of the adequacy of outpatient antidepressant treatment. Psychiatric Services, 54(9), 1233-1239.	Guideline adherence
Williams. (1998). A lack of motivation: Infection control measures. Nursing times, 84(22), 60-64.	Guideline adherence
Won, et al. (2004). Handwashing program for the prevention of nosocomial infections in a neonatal intensive care unit. Infection Control and Hospital Epidemiology, 25(9), 742-746.	Guideline adherence
Madhok, R., Thomson, R. G., Mordue, A., Mendelow, A. D., & Barker, J. (1993). An audit of distribution and use of guidelines for management of head injury. Quality in Health Care, 2(1), 27-30.	Guideline adherence
Bahtsevani, C., Khalaf, A., Willman, A., Bahtsevani, C., Khalaf, A., & Willman, A. (2005). Evaluating psychiatric nurses' awareness of evidence-based nursing publications. Worldviews on Evidence-Based Nursing, 2(4), 196-206.	Guideline adherence
Bahtsevani, C., Willman, A., Khalaf, A., & OÌstman, M. (2008). Developing an instrument for evaluating implementation of clinical practice guidelines: A test-retest study. Journal of Evaluation in Clinical Practice.	Guideline adherence
Hosoglu, S., et al. (2003). A national survey of surgical antibiotic prophylaxis in Turkey. Infection Control and Hospital Epidemiology, 24(10), 758-761.	Guideline adherence
Chen, J., & Zhou, L. P. (2006). A survey of the application of evidence-based medicine in medical postgraduates. Chinese Journal of Evidence-Based Medicine*, 6*(8), 596-599.	Language: Chinese
Zhou, L. P., et al. (2007). Can training courses improve medical postgraduates' knowledge, skill, attitude and behavior related to evidence-based medicine? A before-and-after study. Chinese Journal of Evidence-Based Medicine*, 7*(5), 337-343.	Language: Chinese
Kalliomaki, T. (2002). Midwives as users of research-based knowledge in childbirth nursing. Sairaanhoitaja, 75(4), 14-17.	Language: Finnish

Citation	Exclusion reason
Perleth, M., Jakubowski, E., & Busse, R. (2000). "Best practice" in health care--or why we need evidence-based medicine, guidelines and health technology assessment. Zeitschrift fur Arztliche Fortbildung und Qualitatssicheresearch useng*, 94*(9), 741-744.	Language: German
Puschner, B., Vauth, R., Jacobi, F., & Becker, T. (2006). Evidence basis of psychotherapy for schizophrenia patients in Germany. *Nervenarzt, 77*(11), 1301.	Language: German
Veith, A., Buchbinder, C., & Beelmann, A. (1998). Research orientation in psychotherapeutic postgraduate training programs and psychotherapeutic practice. Verhaltenstherapie, 8(4), 263-269.	Language: German
Oh, E. G., Oh, H. J., & Lee, Y. J. (2004). Nurses' research activities and barriers of research utilization. Taehan Kanho Hakhoe chi, 34(5), 838-848.	Language: Korean.
Bostrom A-M, Wallin L & Nordstrom G. (2006). Research use in the care of older people: A survey among healthcare staff. International Journal of Older People Nursing 1, 131-140 (2006). International Journal of Older People Nursing, 1(4), 252-252. Erratum.	Not an instrument to measure research use. Not on development or use.
Youngstrom, L. H. (1996). Nursing staff development educators and research utilization. (Widener University School of Nursing). (UMI Order #PUZ9709112.)	Not an instrument to measure research use. Not on development or use.
Barg, F. K., McCorkle, R., Robinson, K., Yasko, J. M., Jepson, C., & McKeehan, K. M. (1992). Gaps and contract: Evaluating the diffusion of new information. Cancer Nursing, 15(6), 401-405.	Not an instrument to measure research use. Not on development or use.
Bjorkstrom, M. E., & Hamrin, E. K. F. (2001). Swedish nurses' attitudes towards research and development within nursing. Journal of Advanced Nursing, 34(5), 706-714.	Not an instrument to measure research use. Not on development or use.
Ehrenfeld, M., & Eckerling, S. (1991). Perceptions and attitudes of registered nurses to research: A comparison with a previous study. Journal of Advanced Nursing16, 224-232.	Not an instrument to measure research use. Not on development or use.
Eckerling, S., Bergman, R., Bar-Tal, Y (1998). Perceptions and attitudes of academic nursing students to research. Journal of Advanced Nursing, 13, 759-767.	Not an instrument to measure research use. Not on development or use.
Eller, L. S., Kleber, E., & Wang, S. L. (2003). Research knowledge, attitudes and practices of health professionals. Nursing Outlook, 51(4), 165-170.	Not an instrument to measure research use. Not on development or use.
Reed, J. H., & Jordan, G. (2007). Using systems theory and logic models to define integrated outcomes and performance measures in multi-program settings. *Research Evaluation, 16*(3), 169-181.	Not an instrument to measure research use. Not on development or use.
Richey, Blythe, & Berlin. (1987). Do social workers evaluate their practice? Social Work Research & Abstracts, 23, 14-20.	Not an instrument to measure research use. Not on development or use.

Citation	Exclusion reason
Welch. (1983). Will graduates use single-case design to evaluate their casework practice? Journal of Education for Social Work, 19, 42-47.	Not an instrument to measure research use. Not on development or use.
Rangeley, H., & Arthurs, J. (2004). The long-term effects of undertaking a research course on clinical practice. Nurse Education in Practice, 4(1), 12-19.	Not an instrument to measure research use. Not on development or use.
Carlson, C. L., Plonczynski, D. J. (2008). Has the BARRIERS Scale changed nursing practice? An integrative review. Journal of Advanced Nursing*, 63*(4), 322-333.	Not an instrument to measure research use. Not on development or use.
Craik, J., & Rappolt, S. (2006). Enhancing research utilization capacity through multifaceted professional development. American Journal of Occupational Therapy, 60(2), 155-164.	Not an instrument to measure research use. Not on development or use.
Hoffart, N., & Cobb, A. K. (2002). Assessing clinical pathways use in a community hospital: It depends on what "use" means. The Joint Commission Journal on Quality Improvement, 28(4), 167-179.	Not an instrument to measure research use. Not on development or use.
Howell, S. L., Foster, R. L., Hester, N. O., Vojir, C. P., & Miller, K. L. (1996). Evaluating a pediatric pain management research utilization program. Canadian Journal of Nursing Research, 28(2), 37-57.	Not an instrument to measure research use. Not on development or use.
Schreiber, J., & Stern, P. (2005). A review of the literature on evidence-based practice in physical therapy. *Internet Journal of Allied Health Sciences & Practice, 3*(4), 17p.	Not an instrument to measure research use. Not on development or use.
Linde, B. J. (1989). The effectiveness of three interventions to increase research utilization among practicing nurses, The University of Michigan (Thesis).	Not an instrument to measure research use. Not on development or use.
Midodzi, W. K., Hayduk, L., Cummings, G. G., Estabrooks, C. A., & Wallin, L. (2007). An alternative approach to addressing missing indicators in parallel datasets: Research utilization as a phantom latent variable. *Nursing Research, 56*(4 SUPPL. 1).	Not an instrument to measure research use. Not on development or use of research.
Panagiotopoulou, K., & Kerr, S. M. (2002). Pressure area care: An exploration of Greek nurses' knowledge and practice. Journal of Advanced Nursing, 40(3), 285-296.	Not an instrument to measure research use. Not on development or use of research.
Peach, H. (2003). Should Australia's hospitals be reviewing the use of research in patient care by nurses, managers and allied health professionals? A systematic review of recent evidence. Australian Health Review, 26(2), 49-62.	Not an instrument to measure research use. Not on development or use of research.
Pennington, L. (2001). Attitudes to and use of research in speech and language therapy. British Journal of Therapy & Rehabilitation, 8(10), 375-6, 378-9.	Not an instrument to measure research use. Not on development or use of research.

Citation	Exclusion reason
Research utilization (2000). Kentucky Nurse, 48(3), 30-30.	Not an instrument to measure research use. Not on development or use of research.
Smith, T. J. (1997). An examination of the direct consequences and the individual characteristics influencing physician adoption of a practice guideline innovation. (Nova Southeastern University).	Not an instrument to measure research use. Not on development or use of research.
Thompson, C., McCaughan, D., Cullum, N., Sheldon, T., & Raynor, P. (2004). International research reviews. The potential for evidence based primary care nursing: clinical decisions and research information use by UK primary care nurses. Hypothesis: Journal of the Research Section of MLA, 18(1), 7-8.	Not an instrument to measure research use. Not on development or use of research.
Walker, A. E., Grimshaw, J., Johnston, M., Pitts, N., Steen, N., & Eccles, M. (2003). PRIME--Process modeling in Implementation research: Selecting a theoretical basis for interventions to change clinical practice. BMC Health Services Research, 3(1), 22.	Not an instrument to measure research use. Not on development or use of research.
Farbstein, K., & Clough, J. (2001). Improving medication safety across a multihospital system. Joint Commission Journal on Quality Improvement, 27(3), 123-137. (245)	Not an instrument to measure research use. Not on development or use of research. Is about adherence to 16 selected practices to reduce medication errors but the research basis for these are not discussed.
Dorsey, M., Overman, P., Hayden, W. J., Mayberry, W., Requa-Clark, B., & Krust, K. (1991). Relationships among and demographic predictors of dentists' self-reported adherence to national guidelines. Social Science and Medicine, 32(11), 1263-1268.	Not an instrument to measure research use. Not on development or use of research. Is about adherence to national guidelines but the research basis for these guidelines are not discussed.
Hart, G. M. (1988). Change theory and the impact of an educational program on the case-finding activities of registered nurses in the early identification of alcohol-related problems. Rutgers the State University of New Jersey-New Brunswick.	Not an instrument to measure research use. Not on development or use of research. Is about the effects of an educational program on nurses' behaviour but research use is not discussed.
McCleary, L., & Brown, G. T. (2003). Barriers to paediatric nurses' research utilization. Journal of Advanced Nursing, 42(4), 364-372.	Not an instrument to measure research use. Not on development or use of research.

Citation	Exclusion reason
Kirk & Fisher. (1976). Do social workers understand research? Journal of Education for Social Work, 12, 63-70.	Not an instrument to measure research use. Not on development or use of research. Potential for use measured, not actual use.
Fattal, J., Lehoux, P. (2008). Health technology assessment use and dissemination by patient and consumer groups: Why and how? *International Journal of Technology Assessment in Health Care, 24*(4), 473-480.	Not an instrument to measure research use. Not on development or use of research. Purpose is not to measure the amount of HTA/research use but rather to examine *how* HTA reports were used by organizations in relation to four cases.
Van Mullem, et al. (1999). Strategic planning for research use in nursing practice. Journal of Nursing Administration, 29(12), 38-45.	Not an instrument to measure research use. Not on development or use of research. The Kap survey measures willingness but not actual research use
Stock, J. L., et al. (1998). Clinical reporting to primary care physicians leads to increased use and understanding of bone densitometry and affects the management of osteoporosis, Annals of Internal Medicine, 128(12 part 1), 996-999.	Not an instrument to measure research use. Not on development or use of research. The outcome (management of osteoporosis) is not necessarily research based.
Adamsen, L., Larsen, K., Bjerregaard, L., & Madsen, J. K. (2003). Danish research-active clinical nurses overcome barriers in research utilization. Scandinavian Journal of Caring Sciences, 17(1), 57-65.	Not an instrument to measure research use. Not on development or use of research.
Taylor, G., Herrick, T., & Mah, M. (1998). Wound infections after hysterectomy: Opportunities for practice improvement. American Journal of Infection Control, 26(3), 254-257.	Not an instrument to measure research use. Not on development or use of research.
Schmader, K., et al. (1994). Appropriateness of medication prescribing in ambulatory elderly patients. Journal of the American Geriatrics Society, 42(12), 1241-1247.	Not an instrument to measure research use. Not on development or use of research.

Citation	Exclusion reason
Klabunde, C.N. (1997). Physicians' reactions to change in recommendations for mammography screening, American Journal of Preventive Medicine, 13(6), 432-438.	Not an instrument to measure research use. Not on development or use of research.<br>Evidence is inconclusive, measures use of the 'Statement of Evidence"
Lacey, E. A. (1996). Facilitating research-based practice by educational intervention. Nurse Education Today, 16(4), 296-301.	Not an instrument to measure research use. Not on development or use of research. Evaluation of the extent to which an educational intervention can influence practice.
Nelson, A., & Weaver, F. M. (2004). Promoting evidence-based practice in spinal cord injury/disorders health care. Sci Nursing, 21(3), 129-135.	Not an instrument to measure research use. Not on development or use of research.
Miller, L. M., & Nugent, K. P. (2003). Surgical integrated care pathway development: Compliance and staff satisfaction. Journal of Integrated Care Pathways, 7(1), 36-46.	Not an instrument to measure research use. Not on development or use of research.<br>Measures compliance with an integrated care pathway. Research basis for this pathway is not clearly identified.
Weyts, A., Morpeth, L., & Bullock, R. (1999). Department of Health research overviews -- past, present and future: an evaluation of the dissemination of the Blue Book, Child Protection: Messages from research. Child and Family Social Work, 5(3), 215-223.	Not an instrument to measure research use. Not on development or use of research.<br>Measures use of exercises in a research-based report, but, from what is described, these exercises are for the physicians to evaluate their practice and attitudes, not to change them.
Lombarts, M. J. M. H., Klazinga, N. S., & Redekop, K. (2005). Measuring the perceived impact of facilitation on implementing recommendations from external assessment: Lessons from the Dutch visitatie programme for medical specialists. Journal of Evaluation in Clinical Practice, 11(6), 587-597.	Not an instrument to measure research use. Not on development or use of research.

Citation	Exclusion reason
Landon, B. E., et al. (2003). Physician specialization and antiretroviral therapy for HIV - adoption and use in a national probability sample of persons infected with HIV. Journal of General Internal Medicine, 18(4).	Not an instrument to measure research use. Not on development or use of research. Physician characteristics are the independent variables here (for pt use of HAART meds); their prescribing practice is not asked.
Melzer, B. A., Hubbard, S. M., & Huang, J. Y. (2003). TIPs evaluation project prospective study. Evaluation and program planning, 26(1), 81-89.	Not an instrument to measure research use. Not on development or use of research.<br>Research basis for the Treatment Improvement Protocol (TIP) is not discussed.
Vaidyanathan, V. T. (2004). Looking beyond the adoption decision in innovation research investigating innovation, Ohio: The Ohio State University (Thesis).	Not an instrument to measure research use. Not on development or use of research. State that the EBPs chosen have variable scientific support.
Wenghofer, E. F., Way, D., Moxam, R. S., Wu, H., Faulkner, D., & Klass, D. J. (2006). Effectiveness of an enhanced peer assessment program: Introducing education into regulatory assessment. Journal of Continuing Education in the Health Professions, 26(3), 199-208.	Not an instrument to measure research use. Not on development or use of research. States that physician change practice based on assessor's recommendations but does not state what the basis is (research or otherwise) for these recommendations.
Whellan, D. J., Cohen, E. J., Matchar, D. B., & Califf, R. M. (2002). Disease management in healthcare organizations: results of in-depth interviews with disease management decision makers. American Journal of Managed Care, 8(7), 633-641.	Not an instrument to measure research use. Not on development or use of research.
Mutschler, E. (1984). Evaluating practice - a study of research utilization by practitioners. Social Work, 29(4), 332-337.	Not an instrument to measure research use. Not on development or use of research. Use of research methods, not of research information.
Camiletti, Y. A., & Huffman, M. C. (1998). Research utilization: Evaluation of initiatives in a public health nursing division. Canadian Journal of Nursing Administration, 11(2), 59-77.	Not an instrument to measure research use. Not on development or use of research. Valuing research.

Citation	Exclusion reason
Ferlie, E., Fitzgerald, L., & Wood, M. (2000). Getting evidence into clinical practice: An organizational behaviour perspective. Journal of Health Services & Research Policy, 5(2), 96-102.	Not an instrument to measure research use. Not on development or use of research. Case study of change issues.
Jacobson, A. F., et al. (2008). Factors influencing nurses' participation in clinical research. Gastroenterology Nursing, 31(3), 198-208.	Not an instrument to measure research use. Not on development or use of research.
Murtaugh, C. M., Pezzin, L. E., McDonald, M. V., Feldman, P. H., & Peng, T. R. (2005). Just-in-time evidence-based e-mail "reminders" in home health care: Impact on nurse practices. Health Services Research, 40(3), 849-864.	Not an instrument to measure research use. Not on development or use of research. About cost.
McCleary, L., Ellis, J. A., & Rowley, B. (2004). Evaluation of the pain resource nurse role: A resource for improving pediatric pain management. Pain Management Nursing, 5(1), 29-36.	Not an instrument to measure research use. Not on development or use of research. About how a program influenced a specialists' role.
Michie, S., & Johnston, M. (2004). Improving health care delivery: Making psychological theory useful. Psychology and Health, 19(SUPPL. 1), 113-114.	Not an instrument to measure research use. Not on development or use of research.
Crowe (1996). Making best use of research evidence -- a course for MSLC members. Changing Childbirth Update (5), 8-8.	Not an instrument to measure research use. Not on development or use of research. Advertisement for a workshop.
Forsetlund, L., & Bjorndal, A. (2001). The potential for research-based information in public health: Identifying unrecognized information needs. BMC Public Health, 1, 1.	Not an instrument to measure research use. Not on development or use of research. Assesses information needs.
Cuddihy, J. T. (1979). Clinical research: Translation into nursing practice. International Journal of Nursing Studies, 16(1), 65-72.	Not an instrument to measure research use. Not on development or use of research. Assesses nursing process.
Di Pietro, T., et al. (2008). What nurses want: Diffusion of an innovation. Journal of Nursing Care Quality, *23*(2), 140-146.	Not an instrument to measure research use. Not on development or use of research. Assesses what nurses want to make decisions.

Citation	Exclusion reason
Clifford, C. M., Murray, S., & Kelly, S. M. (2001). A multiprofessional perspective of the role and training needs for research utilisation in healthcare. Journal of Clinical Excellence*, 3*(4), 175-182.	Not an instrument to measure research use. Not on development or use of research. Assesses attitudes.
Gray, M. J., Elhai, J. D., & Schmidt, L. O. (2007). Trauma professionals' attitudes toward and utilization of evidence-based practices. Behavior Modification*,* 31(6), 732-748.	Not an instrument to measure research use. Not on development or use of research. Assesses attitudes.
Larrabee, J. H., et al. (2007). Evaluation of a program to increase evidence-based practice change. Journal of Nursing Administration, 37(6), 302-310.	Not an instrument to measure research use. Not on development or use of research. Assesses attitudes.
Mehrdad, N., Salsali, M., & Kazemnejad, A. (2008). Iranian nurses' attitudes toward research utilisation. Journal of Research in Nursing, 13(1), 53-65.	Not an instrument to measure research use. Not on development or use of research. Assesses attitudes.
Mildon, D., Courtright, P., Rollins, D., Blicker, J., & Law, F. (2001). Knowledge, attitudes and practices regarding evidence-based medicine and outcome assessment: A survey of British Columbia cataract surgeons. Canadian Journal of Ophthalmology-Journal Canadien D Ophtalmologie, 36(6), 323-331.	Not an instrument to measure research use. Not on development or use of research. Assesses attitudes.
Moledor, H. (1999). Conduction and utilization of research: The relationship between air force nurses' attitudes, levels of education, and rank. Uniformed Services University of the Health Sciences (Thesis).	Not an instrument to measure research use. Not on development or use of research. Assesses attitudes.
Thibodeau, J. A., & Hawkins, J. W. (1994). Moving toward a nursing model in advanced practice. Western Journal of Nursing Research, 16(2), 205-218.	Not an instrument to measure research use. Not on development or use of research. Assesses attitudes.
McClarey, M. (2008). Iranian nurses' attitudes toward research utilisation. Journal of Research in Nursing, 13(1), 66-67.	Not an instrument to measure research use. Not on development or use of research. Assesses attitudes.
Metcalfe, C., Lewin, R., Wisher, S., Perry, S., Bannigan, K., & Moffett, J. K. (2001). Barriers to implementing the evidence base in four NHS therapies: Dietitians, occupational therapists, physiotherapists, speech and language therapists. Physiotherapy, 87(8), 433-441.	Not an instrument to measure research use. Not on development or use of research. Assesses barriers.

Citation	Exclusion reason
Parahoo, K. (2000). Barriers to, and facilitators of, research utilization among nurses in Northern Ireland. Journal of Advanced Nursing, 31(1), 89-98.	Not an instrument to measure research use. Not on development or use of research. Assesses barriers.
Mitton, C., & Patten, S. (2004). Evidence-based priority-setting: What do the decision-makers think? Journal of Health Services Research and Policy, 9(3), 146-152.	Not an instrument to measure research use. Not on development or use of research. Assesses barriers.
Michie, S., Hendy, J., Smith, J., & Adshead, F. (2004). Evidence into practice: A theory based study of achieving national health targets in primary care. Journal of Evaluation in Clinical Practice, 10(3), 447-456.	Not an instrument to measure research use. Not on development or use of research.
Chummun, H., Tiran, D. (2008). Increasing research evidence in practice: A possible role for the consultant nurse. Journal of Nursing Management, 16(3), 327-333.	Not an instrument to measure research use. Not on development or use of research. Commentary
Gawlinski, A. (2007). Evidence-based practice changes: Measuring the outcome. AACN Advanced Critical Care, 18(3), 320-322.	Not an instrument to measure research use. Not on development or use of research. Commentary
Sisson, J. (2002). Evidence based practice. Journal of Community Nursing, 16(5).	Not an instrument to measure research use. Not on development or use of research. Commentary.
Treasure, T. (2006). The evidence on which to base practice: Different tools for different times. European Journal of Cardio-Thoracic Surgery, 30(6), 819-824.	Not an instrument to measure research use. Not on development or use of research. Commentary.
Meijers, J. M. M., Janssen, M. A. P., Cummings, G. G., Wallin, L., Estabrooks, C. A., & Halfens, R. Y. G. (2006). Assessing the relationships between contextual factors and research utilization in nursing: Systematic literature review. Journal of Advanced Nursing, 55(5), 622-635.	Not an instrument to measure research use. Not on development or use of research. Assesses context.
Block, A. E. (2007). The diffusion of medical information in hospitals, patients and physicians. Harvard University, United States -- Massachusetts.	Not an instrument to measure research use. Not on development or use of research. Cost benefit analysis.
Shortell, S. M., et al. (2001). Implementing evidence-based medicine: The role of market pressures, compensation incentives, and culture in physician organizations. Medical Care, 39(7 SUPPL.).	Not an instrument to measure research use. Not on development or use of research. Assesses culture, compensation measures.

Citation	Exclusion reason
Bostrom, J., & Wise, L. (1994). Closing the gap between research and practice... "Retrieval and Application of Research in Nursing". Journal of Nursing Administration, 24(5), 22-27.	Not an instrument to measure research use. Not on development or use of research. Describes a program.
Weiss, A. (1994). Adoption Of innovation and policy-making in organizations questionnaire identifying sources of error in informant reports: A confirmatory measurement model approach. Evaluation Review, 18, 592-612.	Not an instrument to measure research use. Not on development or use of research. Describes how to do confirmatory factor analysis.
Greer, A. L. (1977). Advances in the study of diffusion of innovation in health care organizations. Milbank Memorial Fund Quarterly/Health and Society, 55(4), 505-532.	Not an instrument to measure research use. Not on development or use of research. Assesses diffusion factors.
Upton, J. (2007). Part 42: Research evidence: Finding it and using it. Practice Nurse, 34(4), 43-48.	Not an instrument to measure research use. Not on development or use of research. Discussed how to do a literature search.
Pearson, B. (1994). Translating research into practice. Journal of Urological Nursing, 13(3), 838-842.	Not an instrument to measure research use. Not on development or use of research.
Simpson, D. D. (2002). A conceptual framework for transferring research to practice. Journal of Substance Abuse Treatment, 22(4), 171-182.	Not an instrument to measure research use. Not on development or use of research.
Hart, P., et al. (2008). Effectiveness of a computer-based educational program on nurses' knowledge, attitude, and skill level related to evidence-based practice. Worldviews on Evidence-Based Nursing, 5(2), 75-84.	Not an instrument to measure research use. Not on development or use of research. Assesses EBP Skills.
Haines, A., & Donald, A. (1998). Getting research findings into practice. Making better use of research findings. British Medical Journal, 317(7150), 72-75.	Not an instrument to measure research use. Not on development or use of research. Editorial.
Pipe, T. B., Cisar, N. S., Caruseso, E., Wellik, K. E., Pipe, T. B., Cisar, N. S., et al. (2008). Leadership strategies: Inspiring evidence-based practice at the individual, unit, and organizational levels. Journal of Nursing Care Quality, 23(3), 265-271.	Not an instrument to measure research use. Not on development or use of research. Education interventions for leadership.

Citation	Exclusion reason
Melnyk, B. M. (2007). Enhancing research utilization capacity through multifaceted professional development. Worldviews on Evidence-Based Nursing, 4(3), 172-173.	Not an instrument to measure research use. Not on development or use of research. Evidence Digest.
Sheehan, A., Walrath-Greene, C., Fisher, S., Crossbear, S., & Walker, J. (2007). Evidence-based practice knowledge, use, and factors that influence decisions: Results from an evidence-based practice survey of providers in American Indian/ Alaska Native communities. American Indian and Alaska Native Mental Health Research, 14(2), 29-48.	Not an instrument to measure research use. Not on development or use of research. Evidence/knowledge use in native communities.
Regan, J. A. (1998). Will current clinical effectiveness initiatives encourage and facilitate practitioners to use evidence-based practice for the benefit of their clients? Journal of Clinical Nursing, 7(3), 244-250.	Not an instrument to measure research use. Not on development or use of research. Facilitators of change.
Dobbins, M., Cockerill, R., Barnsley, J., & Ciliska, D. (2001). Factors of the innovation, organization, environment, and individual that predict the influence five systematic reviews had on public health decisions. International Journal of Technology Assessment in Health Care, 17(4), 467-478.	Not an instrument to measure research use. Not on development or use of research.
Sredl, D., & Sredl, D. (2008). Evidence-based nursing practice: What US nurse executives really think. Nurse Researcher, 15(4), 51-67.	Not an instrument to measure research use. Not on development or use of research. Factors related to EBP.
Redfern, S., & Murrells, T. (1998). Occasional paper. Research, audit and networking: Who's in the lead? Nursing Times, 94(28), 57-60.	Not an instrument to measure research use. Not on development or use of research. Factors to build a research culture.
Kenrick, M., & Luker, K. A. (1996). An exploration of the influence of managerial factors on research utilization in district nursing practice. Journal of Advanced Nursing, 23(4), 697-704.	Not an instrument to measure research use. Not on development or use of research.
Guenter, D., et al. (2005). Community-based HIV education and prevention workers respond to a changing environment. Journal of the Association of Nurses in AIDS Care, 16(1), 29-36.	Not an instrument to measure research use. Not on development or use of research. HIV Prevention Program.
Oermann, M. H., Roop, J. C., Nordstrom, C. K., Galvin, E. A., & Floyd, J. A. (2007). Effectiveness of an intervention for disseminating Cochrane reviews to nurses. MEDSURG Nursing, 16(6), 373-377.	Not an instrument to measure research use. Not on development or use of research.

Citation	Exclusion reason
Mulhall, A., le May, A., & Alexander, C. (1996). The utilization of research in nursing: A report of a study involving nurses and managers. Professional Update, 4(7), 50-51.	Not an instrument to measure research use. Not on development or use of research. Interview uncovering factors related to research utilization.
Luker, K. A., & Kenrick, M. (1995). Towards knowledge-based practice: An evaluation of a method of dissemination. International Journal of Nursing Studies, 32(1), 59-67.	Not an instrument to measure research use. Not on development or use of research. Items measure change to knowledge, not practice.
Munroe, D., Duffy, P., & Fisher, C. (2008). Nurse knowledge, skills, and attitudes related to evidence-based practice: Before and after organizational supports. Medsurg Nursing: Official Journal of the Academy of Medical-Surgical Nurses, 17(1), 55-60.	Not an instrument to measure research use. Not on development or use of research.
Daigle-LeBlanc, M. B. (2002). Measuring knowledge use in organizations. Saint Mary's University, Canada.	Not an instrument to measure research use. Not on development or use of research. Knowledge not required to be research based.
Black, S. D. (1975). The use of research. Journal of the Royal College of Surgeons of Edinburgh, 20(6), 355-364.	Not an instrument to measure research use. Not on development or use of research. Lecture to physicians.
Clifford, C., & Murray, S. (2001). Pre- and post-test evaluation of a project to facilitate research development in practice in a hospital setting. Journal of Advanced Nursing, 36(5), 685-695.	Not an instrument to measure research use. Not on development or use of research, is a report on factors related to research use without measuring research use.
Cheatham. (1987). The empirical evaluation of clinical practice: A survey of four groups of practitioners. Journal of Social Service Research, 10, 163-177.	Not an instrument to measure research use. Not on development or use of research. Measures level of integration of research procedures into practice (self-evaluation), not research use.
Pronovost, P., et al. (2006). How will we know patients are safer? An organization-wide approach to measuring and improving safety. Critical Care Medicine, 34(7), 1988-1995.	Not an instrument to measure research use. Not on development or use of research. Measures of safety

Citation	Exclusion reason
Holden, J. (2002). St. Helens and Knowsley MAAG 1991-2001: Were we effective? Journal of Clinical Governance, 10(3), 139-149.	Not an instrument to measure research use. Not on development or use of research. Measures the effectiveness of audit and feedback program.
Eldridge (1983). Practitioners and self-evaluation. Social Casework, 64, 426-430.	Not an instrument to measure research use. Not on development or use of research. Measures use of research for self-evaluation, not for practice.
Hayashi, S. W., Suzuki, M., Hubbard, S. M., Huang, J. Y., & Cobb, A. M. (2003). A qualitative study of the treatment improvement protocols (TIPS): A qualitative study of the use of TIPs by individuals affiliated with the addiction technology transfer centres (ATTCs). Evaluation and Program Planning, 26(1), 69-79.	Not an instrument to measure research use. Not on development or use of research. Measures use of tips (treatment improvement protocols) but does not present it as a research use measure.
Cobban, S. J., Edgington, E. M., Clovis, J. B.(2008). Moving research knowledge into dental hygiene practice. Journal of Dental Hygiene, 82(2), 21.	Not an instrument to measure research use. Not on development or use of research, report on related factors but not on measure of research use (complexity of research use).
Kiresuk (1993). Nonspecific knowledge transfer and utilization intervention scale. The evaluation of knowledge utilization: Placebo and nonspecific effects, dynamical systems, and chaos theory. Journal of the American Society for Information Science, 44, 235-241.	Not an instrument to measure research use. Not on development or use of research.
Manion (1993). ENA'S Nursing Scan in Emergency Care, 3(6), 16-16.<br><br>Retrieved from this ref: Drury, T. (1993). Commentary on Chaos or transformation? Managing innovation [original article by Manion J appears in JONA 1993;23(5):41-8].	Not an instrument to measure research use. Not on development or use of research.
Matejko, A. J. (1983). Utilization of social research. The Alberta case. Sociologia Internationalis, 21(1 - 2), 117-144.	Not an instrument to measure research use. Not on development or use of research.
Wimpenny, P., et al. (2008). Tracing and identifying the impact of evidence-use of a modified pipeline model. Worldviews on Evidence-Based Nursing, 5(1), 3-12.	Not an instrument to measure research use. Not on development or use of research, is a model.

Citation	Exclusion reason
Gartenberg, M. J. (2007). A study of the role of psychologists practicing in long-term care. Rutgers The State University of New Jersey, Graduate School of Applied and Professional Psychology, United States .	Not an instrument to measure research use. Not on development or use of research.
Sales, A. E. (2007). A view from health services research and outcomes measurement. Nursing Research, 56(4 Suppl), S67-71.	Not an instrument to measure research use. Not on development or use of research. A Critique.
Jbilou, J., Amara, N., Landry, R.(2007). Research-based-decision-making in Canadian health organizations: A behavioural approach. Journal of Medical Systems, 31(3), 185-196.	Not an instrument to measure research use. Not on development or use of research, is a report on factors related to research use without measuring research use.
Koehn, M. L., Lehman, K., Koehn, M. L., & Lehman, K. (2008). Nurses' perceptions of evidence-based nursing practice. Journal of Advanced Nursing, 62(2), 209-215.	Not an instrument to measure research use. Not on development or use of research, is a report on factors related to research use without measuring research use.
Kuuppelomaki, M., & Tuomi, J. (2005). Finnish nurses' attitudes towards nursing research and related factors. International Journal of Nursing Studies, 42(2), 187-196.	Not an instrument to measure research use. Not on development or use of research, is a report on factors related to research use without measuring research use.
Nursing Standard (2005). Using research in practice. 19(26), 30-31.	Not an instrument to measure research use. Not on development or use of research, is a report on factors related to research use without measuring research use.
Thompson, D. S., et al. (2007). Interventions aimed at increasing research use in nursing: A systematic review. Implementation Science, 2, 15.	Not an instrument to measure research use. Not on development or use of research, is a report on factors related to research use without measuring research use.

Citation	Exclusion reason
Rodgers, S. (1994). An exploratory study of research utilization by nurses in general medical and surgical wards. JAN, 20, 904-911.	Not an instrument to measure research use. Not on development or use of research, is a report on factors related to research use without measuring research use.
Thompson, C. (2002). Nurses' use of research information in clinical decision making a descriptive and analytical study: final report, from http://www.york.ac.uk/healthsciences/centres/evidence/decrpt.pdf	Not an instrument to measure research use. Not on development or use of research, is a report on factors related to research use without measuring research use.
Pearcey (1995). Research Skills Questionnaire "Nurses And Tutors" (1995). Achieving research-based nursing practice. Journal of Advanced Nursing, 22, 33-39.	Not an instrument to measure research use. Not on development or use of research, is a report on factors related to research use without measuring research use.
Robinson et al. (2000). Attitudes toward research questionnaire: What are the attitudes of general practitioners towards research? British Journal of General Practice, 50, 390-392.	Not an instrument to measure research use. Not on development or use of research, is a report on factors related to research use without measuring research use.
Upton, D., & Upton, P. (2005). Professional issues: Nurses' attitudes to evidence-based practice: impact of a national policy. British Journal of Nursing (BJN), 14(5), 284-288.	Not an instrument to measure research use. Not on development or use of research, is a report on factors related to research use without measuring research use.
McWilliam, C. L., et al. (2008). Accelerating client-driven care: Pilot study for a social interaction approach to knowledge translation. Canadian Journal of Nursing Research, 40(2), 58-74.	Not an instrument to measure research use. Not on development or use of research, is a report on factors related to research use without measuring research use.

Citation	Exclusion reason
Thompson, C., McCaughan, D., Cullum, N., Sheldon, T., & Raynor, P. (2005). Barriers to evidence-based practice in primary care nursing: Viewing decision-making as context is helpful. Journal of Advanced Nursing, 52(4), 432-444.	Not an instrument to measure research use. Not on development or use of research, is a report on factors related to research use without measuring research use.
Thompson, D. R., Chau, J. P. C., & Lopez, V. (2006). Barriers to, and facilitators of, research utilisation: A survey of Hong Kong registered nurses. International Journal of Evidence-Based Healthcare, 4(2), 77-82.	Not an instrument to measure research use. Not on development or use of research, is a report on factors related to research use without measuring research use.
Pepler, C. J., et al. (2006). Strategies to increase research-based practice: Interplay with unit culture. Clinical Nurse Specialist: The Journal for Advanced Nursing Practice, 20(1), 23-33.	Not an instrument to measure research use. Not on development or use of research, is a report on factors related to research use without measuring research use.
Scott, S. D., Pollock, C. (2008). The role of nursing unit culture in shaping research utilization behaviors. Research in Nursing & Health, 31(4), 298-309.	Not an instrument to measure research use. Not on development or use of research, is a report on factors related to research use without measuring research use.
Titler, M. (2007). Translating research into practice. American Journal of Nursing, 107(6 Suppl), 26-33; quiz 33.	Not an instrument to measure research use. Not on development or use of research, is a report on factors related to research use without measuring research use.
Titler, M. G., et al. (2001). The Iowa Model of evidence-based practice to promote quality care. Critical Care Nursing Clinics of North America, 13(4), 497-509.	Not an instrument to measure research use. Not on development or use of research, is a report on factors related to research use without measuring research use.

Citation	Exclusion reason
Thomas, D. E., Kukuruzovic, R., Martino, B., Chauhan, S. S., & Elliott, E. J. (2003). Knowledge and use of evidence-based nutrition: A survey of paediatric dietitians. Journal of Human Nutrition and Dietetics, 16(5), 315-322.	Not an instrument to measure research use. Not on development or use of research, is a report on factors related to research use without measuring research use.
Stetler, C. B., Caramanica, L. (2007). Evaluation of an evidence-based practice initiative: Outcomes, strengths and limitations of a retrospective, conceptually based approach. Worldviews on Evidence-Based Nursing, 4(4), 187-199.	Not an instrument to measure research use. Not on development or use of research, is a report on factors related to research use without measuring research use.
Veeramah, V. (2008). Exploring strategies for promoting the use of research findings in practice. British journal of nursing, 17(7), 466-471.	Not an instrument to measure research use. Not on development or use of research, is a report on factors related to research use without measuring research use.
Upton, D. (1999). Clinical effectiveness: How much do radiographers know about it and what do they think of the concept? Radiography, 5(2), 79-87.	Not an instrument to measure research use. Not on development or use of research, is a report on factors related to research use without measuring research use.
Upton, D., & Upton, P. (2006). Knowledge and use of evidence-based practice by allied health and health science professionals in the United Kingdom. Journal of Allied Health, 35(3), 127-133.	Not an instrument to measure research use. Not on development or use of research, is a report on factors related to research use without measuring research use.
Upton, D., & Upton, P. (2006). Knowledge and use of evidence-based practice of GPs and hospital doctors. Journal of Evaluation in Clinical Practice, 12(3), 376-384.	Not an instrument to measure research use. Not on development or use of research, is a report on factors related to research use without measuring research use.

Citation	Exclusion reason
Sargent, M. M. C. (1984). Influence of psychotherapy research on clinical practice. University of Delaware, United States .	Not an instrument to measure research use. Not on development or use of research, is a report on factors related to research use without measuring research use.
Schreiber, J. M. (2007). Pediatric physical therapists and evidence-based practice: A participatory action research project. Duquesne University.	Not an instrument to measure research use. Not on development or use of research, is a report on factors related to research use without measuring research use.
Stetler, C. B., et al. (2007). Improving quality of care through routine, successful implementation of evidence-based practice at the bedside: An organizational case study protocol using the Pettigrew and Whipp model of strategic change. Implementation Science, 2, 3.	Not an instrument to measure research use. Not on development or use of research, is a report on factors related to research use without measuring research use.
Rye, C. B., & Kimberly, J. R. (2007). Review: The adoption of innovations by provider organizations in health care. Medical Care Research and Review, 64(3), 235-278.	Not an instrument to measure research use. Not on development or use of research, is a report on factors related to research use without measuring research use.
McQueen, J. (2008). Practice development: Bridging the research-practice divide through the appointment of a research lead. British Journal of Occupational Therapy, 71(3), 112-118.	Not an instrument to measure research use. Not on development or use of research, is a report on factors related to research use without measuring research use.
Johnson, L. N., Sandberg, J. G., & Miller, R. B. (2000). Research practices of marriage and family therapists. American Journal of Family Therapy, 28(5), 239-249.	Not an instrument to measure research use. Not on development or use of research, is a report on factors related to research use without measuring research use.
Qian, X., et al. (2006). Evidence-informed obstetric practice during normal birth in China: Trends and influences in four hospitals. BMC Health Services Research, 6, 29.	Not an instrument to measure research use. Not on development or use of research, but reports on patient outcomes.

Citation	Exclusion reason
Zwarenstein, M., & Reeves, S. (2006). Knowledge translation and interprofessional collaboration: Where the rubber of evidence-based care hits the road of teamwork. Journal of Continuing Education in the Health Professions, 26(1), 46-54.	Not an instrument to measure research use. Not on development or use of research, is a report on factors related to research use without measuring research use.
Wickham, S. (1999). Evidence-informed midwifery 2: Using research in midwifery practice. Midwifery Today (52), 39-41.	Not an instrument to measure research use. Not on development or use of research, is a report on factors related to research use without measuring research use.
Zafar, I., Michael, C., & David, J. T. (1998). Clinical effectiveness: The potential for change in maternity care. Journal of Clinical Effectiveness, 3(2), 67.	Not an instrument to measure research use. Not on development or use of research, is a report on factors related to research use without measuring research use.
Van Caulil, G. F., Mombers, C. A. M., & Van Den Beemt, F. C. H. D. (1996). Quantifying the utilization of research: The difficulties and two models to evaluate the utilization of research results. Scientometrics, 37(3), 433-444.	No instrument to measure research use. Not a development or use of research use measure. Discussion of what research use means.
Low, L. K., Miller, J. (2006). A clinical evaluation of evidence-based maternity care using the Optimality Index. JOGNN - Journal of Obstetric, Gynecologic, & Neonatal Nursing, 35(6), 786-793.	Not an instrument to measure research use. Not on development or use of research, is a report on factors related to research use without measuring research use.
Hakkennes, S., Green, S. (2006). Measures for assessing practice change in medical practitioners. Implementation Science, 1, 29.	Not an instrument to measure research use. Not on development or use of research, is a report on factors related to research use without measuring research use.

Citation	Exclusion reason
Hannes, K., Leys, M., Vermeire, E., Aertgeerts, B., Buntinx, F., & Depoorter, A. (2005). Implementing evidence-based medicine in general practice: A focus group based study. BMC Family Practice, 6, 13p.	Not an instrument to measure research use. Not on development or use of research, is a report on factors related to research use without measuring research use.
Forbes, D., & Phillipchuk, D. (2001). The dissemination and use of nursing research. Canadian Nurse, 97(7), 18-22.	Not an instrument to measure research use. Not on development or use of research, is a report on factors related to research use without measuring research use.
Funk, S. G., Tornquist, E. M., & Champagne, M. T. (1989). Application and evaluation of the dissemination model. Western Journal of Nursing Research, 11(4), 486-491.	Not an instrument to measure research use. Not on development or use of research, is a report on factors related to research use without measuring research use.
Gifford, W., et al. (2007). Managerial leadership for nurses' use of research evidence: An integrative review of the literature. Worldviews on Evidence-Based Nursing, 4(3), 126-145.	Not an instrument to measure research use. Not on development or use of research, is a report on factors related to research use without measuring research use.
Gira, E. C., Kessler, M. L., & Poertner, J. (2004). Influencing social workers to use research evidence in practice: Lessons from medicine and the allied health professions. Research on Social Work Practice, 14(2), 68-79.	Not an instrument to measure research use. Not on development or use of research, is a report on factors related to research use without measuring research use.
Hefferin, E. A., Horsley, J. A., & Ventura, M. R. (1982). Promoting research-based nursing: The nurse administrator's role. Journal of Nursing Administration, 12(5), 34-41.	Not an instrument to measure research use. Not on development or use of research, is a report on factors related to research use without measuring research use.

Citation	Exclusion reason
Haug, N. A., et al. (2008). Adoption of evidence-based practices among substance abuse treatment providers. Journal of Drug Education, 38(2), 181-192.	Not an instrument to measure research use. Not on development or use of research, is a report on factors related to research use without measuring research use.
Gervasini, A. (1999). The research process -- part II. Journal of Trauma Nursing, 6(4), 88-97.	Not an instrument to measure research use. Not on development or use of research, is a report on factors related to research use without measuring research use.
Rutledge, D. N., Bookbinder, M. (2002). Processes and outcomes of evidence-based practice. Seminars in Oncology Nursing, 18(1), 3-10.	Not an instrument to measure research use. Not on development or use of research.
Russell, M. N. (1990). Clinical social work: Research and practice. Newbury Park, California: Sage Publications.	Not an instrument to measure research use. Not on development or use of research. Not a primary study.
Scheirer, M. (1982). Measuring the implementation of innovations. Final report to the National Science Foundation from grant no. PRA-8022612. Annandale, Va.: American Research Institute.<br><br>Author referred us to a 1983 article, which reports the main findings from this report.	Not an instrument to measure research use. Not on development or use of research but is a review that reports on implementation of specific practices.
Hivon, M., Lehoux, P., Denis, J. L., & Tailliez, S. (2005). Use of health technology assessment in decision-making: Co-responsibility of users and producers? International Journal of Technology Assessment in Health Care, 21(2), 268-275.	Not an instrument to measure research use. Not on development or use of research.
Happell, B., & Martin, T. (2004). Exploring the impact of the implementation of a nursing clinical development unit program: What outcomes are evident? International Journal of Mental Health Nursing, 13(3), 177-184.	Not an instrument to measure research use. Not on development or use of research.
Peterson, J. C., et al. (2007). A framework for research utilization applied to seven case studies. American Journal of Preventive Medicine, 33(1 Suppl), S21-34.	Not an instrument to measure research use. Not on development or use of research. Instrument's purpose to measure the adoption of a single research-based practice.

Citation	Exclusion reason
Sea-Orchid Group. (2008). Use of evidence-based practices in pregnancy and childbirth: South East Asia Optimising Reproductive and Child Health in Developing countries project. PLoS ONE 3(7)(e2646).	Not an instrument to measure research use. Not on development or use of research. Is a review/synthesis report. Is a report on factors related to research use without measuring research use.
Estabrooks, C. A., Floyd, J. A., Scott-Findlay, S., O'Leary, K. A., & Gushta, M. (2003). Individual determinants of research utilization: A systematic review. Journal of Advanced Nursing, 43(5), 506-520.	Not an instrument to measure research use. Not on development or use of research. Is a review/synthesis report. Is a report on factors related to research use without measuring research use.
Lavis, J., et al. (2003). Measuring the impact of health research. Journal of Health Services & Research Policy, 8(3), 165-170.	Not an instrument to measure research use. Not on development or use of research. Is a review/synthesis report. Is a report on factors related to research use without measuring research use.
Lerner, E. B., Mosesso, V., Jr., Zak, C. (2002). Implementation of research in the out-of-hospital setting. Prehospital Emergency Care, 6(2 Suppl), S24-27.	Not an instrument to measure research use. Not on development or use of research.
Ashford, J. B., & Lecroy, C. W. (1991). Problem-solving in social work practice: Implications for knowledge utilization. Research on Social Work Practice, 1(3), 306-318.	Not an instrument to measure research use. Not on development or use of research
Beyer, J. M., & Trice, H. M. (1982). The utilization process: A conceptual framework and synthesis of empirical findings. Administrative Science Quarterly, 27(4), 591-622.	Not an instrument to measure research use. Not on development or use of research; Is a conceptual paper.
Bircumshaw, D. (1990). The utilization of research findings in clinical nursing practice. Journal of Advanced Nursing, 15(11), 1272-1280.	Not an instrument to measure research use. Not on development or use of research; Is a review or synthesis report.

Citation	Exclusion reason
Adily, A., & Ward, J. (2004). Evidence based practice in population health: A regional survey to inform workforce development and organizational change. Journal of Epidemiology & Community Health, 58(6), 455-460.	Not an instrument to measure research use. Not on development or use of research, report on related factors but not on measure of research use.
Andersson, N., Cederfjall, C., Jylli, L., Nilsson Kajermo, K., & Klang, B. (2007). Professional roles and research utilization in paediatric care: Newly graduated nurses experiences. Scandinavian Journal of Caring Sciences, 21(1), 91-97.	Not an instrument to measure research use. Not on development or use of research, report on related factors but not on measure of research use.
Armitage, S. (1990). Research utilisation in practice. Nurse Education Today, 10(1), 10-15.	Not an instrument to measure research use. Not on development or use of research, report on related factors but not on measure of research use.
Armstrong, R., et al. (2007). The nature of evidence resources and knowledge translation for health promotion practitioners. Health Promotion International, 22(3), 254-260.	Not an instrument to measure research use. Not on development or use of research, report on related factors but not on measure of research use.
Bradbury, L., Clipsham, K., & Kitson, A. (2005). Developing nursing research. Journal of Orthopaedic Nursing, 9(4), 199-204.	Not an instrument to measure research use. Not on development or use of research, report on related factors but not on measure of research use.
Conklin, J., Stolee, P. (2008). A model for evaluating knowledge exchange in a network context. Canadian Journal of Nursing Research, 40(2), 116-124.	Not an instrument to measure research use. Not on development or use of research, report on related factors but not on measure of research use.
Conway, P. H., Edwards, S., Stucky, E. R., Chiang, V. W., Ottolini, M. C., & Landrigan, C. P. (2006). Variations in management of common inpatient pediatric illnesses: Hospitalists and community pediatricians. Pediatrics, 118(2), 441-447.	Not an instrument to measure research use. Not on development or use of research, report on related factors but not on measure of research use.

Citation	Exclusion reason
Danielson, E., & Berntsson, L. (2007). Registered nurses' perceptions of educational preparation for professional work and development in their profession. Nurse Education Today, 27(8), 900-908.	Not an instrument to measure research use. Not on development or use of research, report on related factors but not on measure of research use.
Brenner, M. (2005). Children's nursing in Ireland: Barriers to, and facilitators of, research utilisation. Paediatric Nursing, 17(4), 40-45.	Not an instrument to measure research use. Not on development or use of research, report on related factors but not on measure of research use.
Buxton, V., James, T., & Harding, W. (1998). Occasional paper. Using research in community nursing. Nursing Times, 94(35), 57-60.	Not an instrument to measure research use. Not on development or use of research, report on related factors but not on measure of research use.
Aarons, G. A. (2004). Mental health provider attitudes toward adoption of evidence-based practice: The evidence-based practice attitude scale (EBPAS). Mental Health Services Research, 6(2), 61-74.	Not an instrument to measure research use. Not on development or use of research, report on related factors but not on measure of research use.
Aarons, G. A. (2005). Measuring provider attitudes toward evidence-based practice: Consideration of organizational context and individual differences. Child and Adolescent Psychiatric Clinics of North America, 14(2), 255-271.	Not an instrument to measure research use. Not on development or use of research, report on related factors but not on measure of research use.
Aarons, G. A. (2006). Transformational and transactional leadership: Association with attitudes toward evidence-based practice. Psychiatric Services, 57(8), 1162-1169.	Not an instrument to measure research use. Not on development or use of research, report on related factors but not on measure of research use.
Adams, F., & Cooke, M. (1998). Evidence-based practice. Implementing evidence-based practice for urinary catheterization. British Journal of Nursing (BJN), 7(22), 1393.	Not an instrument to measure research use. Not on development or use of research, purpose is to examine use of specific practice, not to measure research use.
Rabin, B. A., et al. (2008). A glossary for dissemination and implementation research in health. Journal of Public Health Management & Practice, 14(2), 117-123.	Not an instrument to measure research use. Not primary study: a glossary

Citation	Exclusion reason
Camiah, S. (1997). Utilization of nursing research in practice and application strategies to raise research awareness amongst nurse practitioners: A model for success. Journal of Advanced Nursing, 26(6), 1193-1202.	Not an instrument to measure research use. One qualitative question in focus groups with aim of measuring research use.
Roberts, K. L. (1998). Evidence-based practice: An idea whose time has come. Collegian, 5(3), 24-27.	Not an instrument to measure research use. Opinion article
Boissel, J. P., et al. (2005). How to measure non-consistency of medical practices with available evidence in therapeutics: A methodological framework. Fundamental & Clinical Pharmacology, 19(5), 591-596.	Not an instrument to measure research use. Opinion piece.
Bond, M. P. (2000). Information use: Appreciating the subtleties. British Journal of Therapy & Rehabilitation, 7(5), 241-245.	Not an instrument to measure research use. Opinion piece.
Merrin, J. B. (2008). Program evaluation of clinical services at a community-based behavioral health clinic: An action research approach. Alliant International University, Fresno, United States .	Not an instrument to measure research use. Assesses organizational factors.
Pfouts & McDaniels. (1977). Medical handmaidens or professional colleagues: A survey of social work practice in the pediatrics departments of twenty-eight teaching hospitals. Social Work in Health Care, 2I, 275-283.	Not an instrument to measure research use. Participation in research is measured but no clear measure of use.
Redfern, S., et al. (1997). An evaluation of nursing development units... including commentary by Bond S. NT Research, 2(4), 292-304.	Not an instrument to measure research use. Measures predictors.
Nicklin, W., & Stipich, N. (2005). Enhancing skills for evidence-based healthcare leadership: the Executive Training for Research Application (EXTRA) program. Canadian Journal of Nursing Leadership, 18(3), 35-44.	Not an instrument to measure research use. Program evaluation.
Bridges, P. H., Bierema, L. L. (2007). The propensity to adopt evidence-based practice among physical therapists. BMC Health Services Research, 7, 103.	Not an instrument to measure research use. Propensity for EBP
Kelly, J. A., et al. (2000). Bridging the gap between the science and service of HIV prevention: Transferring effective research-based HIV prevention interventions to community AIDS service providers. American Journal of Public Health, 90(7), 1082-1088.	Not an instrument to measure research use. Purpose is to measure model adoption.
Gingerich. (1984). Generalizing single-case evaluation from classroom to practice setting. Journal of Education for Social Work, 20, 74-82.	Not an instrument to measure research use. Purpose is to measure use of single-case evaluation, not research use.

Citation	Exclusion reason
Richens, Y. (2002). Are midwives using research evidence in practice? British Journal of Midwifery, 10(1), 11-16.	Not an instrument to measure research use. Qualitative comments r/elated to research use.
Mueller, C., Degenholtz, H., & Kane, R. (2004). Do evidence-based clinical and administrative policies/practices in nursing homes influence quality? Nursing & Health Policy Review, 3(1), 35-47.	Not an instrument to measure research use. Report on patient outcomes.
Rutledge, D. N., & Donaldson, N. E. (1995). Building organizational capacity to engage in research utilization. Journal of Nursing Administration, 25(10), 12-16.	Not an instrument to measure research use. Reports on factors related to research use in health care organizations.
Paukert, J. L., Chumley-Jones, H. S., & Littlefield, J. H. (2003). Do peer chart audits improve residents' performance in providing preventive care? Academic Medicine, 78(10), S39-S41.	Not an instrument to measure research use. Research basis for the 12 preventative practices is not discussed.
Cummings, G. G., et al. (2007). Influence of organizational characteristics and context on research utilization. Nursing Research, 56(4 Suppl), S24-39.	Not an instrument to measure research use. Research use variable is derived.
Sprang, G., Craig, C., Clark, J. (2008). Factors impacting trauma treatment practice patterns: The convergence/divergence of science and practice. Journal of Anxiety Disorders, 22(2), 162-174.	Not an instrument to measure research use. Used combination of tested and untested practices.
Ricketts, T., Saul, C., Newton, P., & Brooker, C. (2003). Evaluating the development, implementation and impact of protocols between primary care and specialist mental health services. Journal of Mental Health, 12(4), 369-383.	Not an instrument to measure research use.
Kovach, A. C. (1997). Hospital breastfeeding policies in the Philadelphia area: A comparison with the ten steps to successful breastfeeding. Birth, 24(1), 41-48.	Not an instrument to measure research use. Compares hospital breastfeeding practices to WHO/UNIICEF recommendations, which were developed by government ministers from 32 countries. Does not mention research-base.
Thompson, D. S. (2006). Research utilization interventions in nursing. University of Alberta, Canada.	Not an instrument to measure research use. Content analysis of the process of use but level of use is not measured.

Citation	Exclusion reason
Michie, S., et al. (2005). Making psychological theory useful for implementing evidence based practice: A consensus approach. Quality & Safety in Health Care, 14(1), 26-33.	Not an instrument to measure research use. Is a report on factors related to research use without measuring research use.
Dubouloz, C., Egan, M., Vallerand, J., & von Zweck, C. (1999). Occupational therapists' perceptions of evidence-based practice... an earlier version of this paper was presented at the World Federation of Occupational Therapists Conference in Montreal, July 1998. American Journal of Occupational Therapy, 53(5), 445-453.	Not an instrument to measure research use. Assesses perceptions of EBP.
Nelson, D. (1995). Research into research practice. Accident and Emergency Nursing, 3(4), 184-189.	Not an instrument to measure research use. Evaluates whether action plans are being implemented.
Gagnon, M. P., Sanchez, E., & Pons, J. M. (2006). Integration of health technology assessment recommendations into organizational and clinical practice: A case study in Catalonia. International Journal of Technology Assessment in Health Care, 22(2), 169.	Not an instrument to measure research use. Focus on factors related to use of HTA.
Rizzuto, C, et al. (1994). Predictors of nurses' involvement in research activities. Western Journal of Nursing Research 16(2): 193-204.	Not an instrument to measure research use. Focus on research involvement.
Bostrom, J., & Suter, W. N. (1993). Research utilization: Making the link to practice. Journal of Nursing Staff Development, 9(1), 28-34.	Not an instrument to measure research use. Focus on research involvement.
Parkin, C., & Bullock, I. (2005). Evidence-based health care: Development and audit of a clinical standard for research and its impact on an NHS trust. Journal of Clinical Nursing, 14(4), 418-425.	Not an instrument to measure research use. Lacks clarity in reporting. Asks about use of 'best evidence' for practice change.
Ornstein, S., Meiert, P. J., Jenkins, R. G., Wessell, A. M., Nemeth, L. S., & Rose, H. L. (2008). Improving the translation of research into primary care practice: Results of a national quality improvement demonstration project, Joint Commission Journal on Quality and Patient Safety.	Not an instrument to measure research use. Assesses quality outcomes.
Shaffer, C. M. (1996). Support for Research in Hospitals Questionnaire. Hospital research programs and barriers to research utilization. IMAGE: Journal of Nursing Scholarship, 28, 278.	Not an instrument to measure research use. Measures research activities not research use.

Citation	Exclusion reason
Shaffer, C. M. (1994). Staff nurse perceptions of barriers to research utilization and administrative supports for research in hospitals. George Mason University.	Not an instrument to measure research use. Measures research activities not research use.
Stomski, N., Grimmer-Somers, K., & Petkov, J. (2008). A survey of the uptake and implementation of research evidence by South Australian acupuncturists in clinical practice: Attitudes and associated predictive factors. Complementary Therapies in Medicine, 16(4), 199-205.	Not an instrument to measure research use. Measures research importance and barriers.
Kimberly, J., Cook, J. M. (2008). Organizational measurement and the implementation of innovations in mental health services. Administration & Policy in Mental Health, 35(1-2), 11-20.	Not an instrument to measure research use. Review/synthesis report. Is a report on factors related to research use without measuring research use.
Drury, T. (1993). Commentary on chaos or transformation? Managing Innovation, JONA, 23(5), 41-48.	Not an instrument to measure research use. Commentary
Muthard, J. & Joint, A. (1978). Measuring and improving research utilization practices in rehabilitation. Gainesville: Rehabilitation Research Institute, College of Health Related Professions, University of Florida.	Not an instrument to measure research use. Generic tool for evaluation of effects of a program.
Schlamp, F. T. (1975). Researching the use of using research: Final report of the Research Utilization Project in California. Sacramento: California Dept. of Rehabilitation.	Not an instrument to measure research use.
Pettengill (1994). Factors encouraging and discouraging the use of nursing research findings. IMAGE: Journal of Nursing Scholarship, 26, 143-147.	Not an instrument to measure research use. Measures predictors.
Wallin, L., Estabrooks, C. A., Midodzi, W. K., & Cummings, G. G. (2006). Development and validation of a derived measure of research utilization by nurses. Nursing Research, 55(3), 149-160.	No instrument to measure research use. Research use variable is derived.
Cole, N., Tucker, L. J., & Foxcroft, D. R. (2000). Benchmarking evidence-based nursing... including commentary by Thompson C. NT Research, 5(5), 336-345.	Not an instrument to measure research use. Not on development or use of research, report on related factors but not on measure of research use. Assesses aspects of EBP.
Measuring organizational implementation status: CII knowledge implementation index (CKII) (2004). [Austin, Tex.]: Construction Industry Institute.	Not healthcare.
Costa-Mitrano, L. R. (2001). Research and school psychologists: Training, consumption, application, perceptions, and attitudes. Alfred University, United States.	Not healthcare.

Citation	Exclusion reason
Landry, R., Amara, N., & Lamari, M. (2001). Utilization of social science research knowledge in Canada. Research Policy, 30(2), 333-349.	Not healthcare. Also, are asking a different question about researchers' perspective on the respondents' use of research.
Standefer, R. L. Research utilization and the development of research utilization systems.	Not healthcare. No research use measure.
Research utilization and the social indicators project (1975). Denver: The Center.	Not healthcare. Research use not measured.
Smith, H., Brown, H., Hofmeyr, G. J., & Garner, P. (2004). Evidence-based obstetric care in South Africa--influencing practice through the 'Better Births Initiative'. South African Medical Journal.Suid-Afrikaanse Tydskrif Vir Geneeskunde, 94(2), 117-120.	Purpose is to measure use of specific practice, not to measure research use.
Abouzelof, R. H. (1999). Diffusion of innovations: Describing the perceptions of the stages in the innovation-decision process for handwashing and alcohol hand rubs. (University of Utah College of Nursing).	Purpose is to measure use of specific practice, not to measure research use.
Amsallem, E., et al. (2007). Evaluation of two evidence-based knowledge transfer interventions for physicians. A cluster randomized controlled factorial design trial: The CardioDAS study	Purpose is to measure use of specific practice, not to measure research use.
Andrzejewski, M. E., Kirby, K. C., Morral, A. R., & Iguchi, M. Y. (2001). Technology transfer through performance management: the effects of graphical feedback and positive reinforcement on drug treatment counselors' behavior. Drug & Alcohol Dependence, 6.	Purpose is to measure use of specific practice, not to measure research use.
Bjornson, D.C. (1990). Impact of a drug-use review program intervention on prescribing after publication of a randomized clinical trial. American Journal of Hospital Pharmacy, 47(7), 1541-154.	Purpose is to measure use of specific practice, not to measure research use.
Bookbinder, M. I. (1992). Nurse linkage agents' efforts to facilitate the use of a research-based innovation. (New York University) (UMI Order #PUZ9237737.)	Purpose is to measure use of specific practice, not to measure research use.
Cantor, M. N., et al. (2005). Barriers to implementing a surgical beta-blocker protocol. Joint Commission Journal on Quality and Patient Safety / Joint Commission Resources, 31(11), 640-648.	Purpose is to measure use of specific practice, not to measure research use.
Capra. (1992). RNs utilization of research findings. The American Journal of Advanced Nursing, 10(1), 21-25.	Purpose is to measure use of specific practice, not to measure research use.
Carlson, C. L. (2006). Prior conditions influencing nurses' decisions to adopt evidence-based postoperative pain assessment practices. (Indiana University).	Purpose is to measure use of specific practice, not to measure research use.

Citation	Exclusion reason
Chien, C. R., & Lai, M. S. (2006). Trends in the pattern of care for lung cancer and their correlation with new clinical evidence: Experiences in a university-affiliated medical center. American Journal of Medical Quality, 21(6), 408-414.	Purpose is to measure use of specific practice, not to measure research use.
ColonEmeric, C., et al. (2006). Translating evidence-based falls prevention into clinical practice in nursing facilities: Results and lessons from a quality improvement collaborative. Journal of the American Geriatrics Society, 54(9), 1414-1418.	Purpose is to measure use of specific practice, not to measure research use.
Davies, B. L. (1999). Evaluation of two strategies for the transfer of research results about labour support and electronic fetal monitoring into practice. (University of Toronto, Canada).	Purpose is to measure use of specific practice, not to measure research use.
Dennis, A. R., Leeson-Payne, C. G., Langham, B. T., & Aitkenhead, A. R. (1995). Local anaesthesia for cannulation. Has practice changed? Anaesthesia, 50(5), 400-402.	Purpose is to measure use of specific practice, not to measure research use.
Doerflinger, D. M. (2004). The relationship between acute care nurse administrators' knowledge and attitudes and restraint reduction. (George Mason University).	Purpose is to measure use of specific practice, not to measure research use.
Dopson, S., Mant, J., & Hicks, N. (1994). Getting research into practice: Facing the issues. Journal of Management in Medicine, 8(6), 4-12.	Purpose is to measure use of specific practice, not to measure research use.
Drenning, C. (2006). Collaboration among nurses, advanced practice nurses, and nurse researchers to achieve evidence-based practice change. Journal of Nursing Care Quality, 21(4), 298-301.	Purpose is to measure use of specific practice, not to measure research use.
Fineberg, H. V., Gabel, R. A., & Sosman, M. B. (1978). Acquisition and application of new medical knowledge by anesthesiologists: Three recent examples. Anesthesiology, 48(6), 430-436.	Purpose is to measure use of specific practice, not to measure research use.
Frantz, R. A., Gardner, S., Harvey, P., & Specht, J. (1992). Adoption of research-based practice for treatment of pressure ulcers in long-term care. Decubitus, 5(1), 44-5, 48-50, 52.	Purpose is to measure use of specific practice, not to measure research use.
Freeman, C. K. et al. (1993). Breastfeeding care in Ohio hospitals.	Purpose is to measure use of specific practice, not to measure research use.
Ghali, W. A., & Cornuz, J. (2000). Early uptake of research findings after fast-track publication. Lancet, 355(9203), 579-580.	Purpose is to measure use of specific practice, not to measure research use.
Gordon, M., & Montgomery, L. A. (1996). Minimizing epidermal stripping in the very low birth weight infant: Integrating research and practice to affect infant outcome. Neonatal Network: NN, 15(1), 37-44.	Purpose is to measure use of specific practice, not to measure research use.
Grap, M. J., Pettrey, L., & Thornby, D. (1997). Hemodynamic monitoring: A comparison of research and practice. American Journal of Critical Care, 6(6), 452-456.	Purpose is to measure use of specific practice, not to measure research use.

Citation	Exclusion reason
Hammond, A., & Klompenhouwer, P. (2005). Getting evidence into practice: Implementing a behavioural joint protection education programme for people with rheumatoid arthritis. British Journal of Occupational Therapy, 68(1), 25-33.	Purpose is to measure use of specific practice, not to measure research use.
Harris, M. (1992). The impact of research findings on current practice in relieving postpartum perineal pain in a large district general hospital. Midwifery, 8(3), 125-131.	Purpose is to measure use of specific practice, not to measure research use.
Helberg, D., Mertens, E., Halfens, R. J., & Dassen, T. (2006). Treatment of pressure ulcers: Results of a study comparing evidence and practice. Ostomy Wound Management, 52(8), 60-72.	Purpose is to measure use of specific practice, not to measure research use.
Henderson, J. L., MacKay, S., & Peterson-Badali, M. (2006). Closing the research-practice gap: Factors affecting adoption and implementation of a children's mental health program. Journal of Clinical Child & Adolescent Psychology, 35(1), 2-12.	Purpose is to measure use of specific practice, not to measure research use.
Hermann, R. C., Ettner, S. L., Dorwart, R. A., LangmanDorwart, N., & Kleinman, S. (1999). Diagnoses of patients treated with ECT: A comparison of evidence-based standards with reported use. Psychiatric Services, 50(8), 1059-1065.	Purpose is to measure use of specific practice, not to measure research use.
Jordan, H. S., Burke, J. F., Fineberg, H., & Hanley, J. A. (1983). Diffusion of innovations in burn care: Selected findings. Burns, 9(4), 271-279.	Purpose is to measure use of specific practice, not to measure research use
Kaner, E. F. S., Lock, C. A., McAvoy, B. R., Heather, N., & Gilvarry, E. (1999). A RCT of three training and support strategies to encourage implementation of screening and brief alcohol intervention by general practitioners. British Journal of General Practice, 49(446), 699-703.	Purpose is to measure use of specific practice, not to measure research use.
Kirchhoff, K. T. (1982). A diffusion survey of coronary precautions. Nursing Research, 31(4), 196-201.	Purpose is to measure use of specific practice, not to measure research use.
LaVela, S. L., Legro, Weaver, & Smith. (2004). Staff influenza vaccination: Lessons learned. SCI Nursing, 21(3), 153-157.	Purpose is to measure use of specific practice, not to measure research use.
Lock, C. A., & Kaner, E. F. S. (2000). Use of marketing to disseminate brief alcohol intervention to general practitioners: Promoting health care interventions to health promoters. Journal of Evaluation in Clinical Practice, 6(4), 345-357**.**	Purpose is to measure use of specific practice, not to measure research use.
Mant, J., Hicks, N. R., Dopson, S., & Hurley, P. (1999). Uptake of research findings into clinical practice: A controlled study of the impact of a brief external intervention on the use of corticosteroids in preterm delivery.	Purpose is to measure use of specific practice, not to measure research use.
Martin, P., Thomsen, A. S., Rautanen, K., Hjalt, C. A., Jonsson, A., & Lofroth, G. (1999). Diffusion of knowledge of Helicobacter pylori and its practical application by Nordic clinicians. Scandinavian Journal of Gastroenterology, 34(10), 974-980.	Purpose is to measure use of specific practice, not to measure research use.

Citation	Exclusion reason
McGovern, M. P., Fox, T. S., Xie, H., & Drake, R. E. (2004). A survey of clinical practices and readiness to adopt evidence-based practices: Dissemination research in an addiction treatment system. Journal of Substance Abuse Treatment, 26(4), 305-312.	Purpose is to measure use of specific practice, not to measure research use.
McMenamin, S. B. et al. (2002) Support for smoking cessation interventions in physician organizations: Institutional and resource dependence perspectives. California: University of California.	Purpose is to measure use of specific practice, not to measure research use.
Morse, B. A., Idelson, R. K., Sachs, W. H., Weiner, L., & Kaplan, L. C. (1992). Pediatricians' perspectives on fetal alcohol syndrome. Journal of Substance Abuse, 4(2), 187-195.	Purpose is to measure use of specific practice, not to measure research use.
Mullen, P. D., Ito, J. R., Carbonari, J. P., & DiClemente, C. C. (1991). Assessing the congruence between physician behavior and expert opinion in smoking cessation counseling. Addictive Behaviors, 16(5), 203-210.	Purpose is to measure use of specific practice, not to measure research use.
Mullenbach, D. M. (1997). Pédiatrie Endotracheal Suctioning: Practice and Complications. Winona, Minn: Winona State University.	Purpose is to measure use of specific practice, not to measure research use.
Munschauer, F. E., Priore, R. L., Hens, M., & Castilone, A. (1997). Thromboembolism prophylaxis in chronic atrial fibrillation: Practice patterns in community and tertiary-care hospitals. Stroke, 28(1), 72-76.	Purpose is to measure use of specific practice, not to measure research use.
Obyrne, K. K., Peterson, L., & Saldana, L. (1997). Survey of pediatric hospitals' preparation programs: Evidence of the impact of health psychology research. Health Psychology, 16(2), 147-154.	Purpose is to measure use of specific practice, not to measure research use.
Rappolt, S., Mitra, A. L., & Murphy, E.. (2002). Professional accountability in restructured contexts of occupational therapy practice. Canadian Journal of Occupational Therapy - Revue Canadienne d Ergotherapie, 69(5), 293-302.	Purpose is to measure use of specific practice, not to measure research use.
Rappolt, S., Pearce, K., McEwen, S., & Polatajko, H. J. (2005). Exploring organizational characteristics associated with practice changes following a mentored online educational module. Journal of Continuing Education in the Health Professions, 25(2), 116.	Purpose is to measure use of specific practice, not to measure research use.
Riegel, B., Thomason, T., Carlson, B., & Gocka, I. (1996). Are nurses still practicing coronary precautions? A national survey of nursing care of acute myocardial infarction patients. American Journal of Critical Care, 5(2), 91-98.	Purpose is to measure use of specific practice, not to measure research use.
Scorpiglione, N., et al. (1995). Appropriateness and variation of surgical treatment of breast cancer in Italy: When excellence in clinical research does not match with generalized good. Clin Epidemiol, 48(3):345–352.	Purpose is to measure use of specific practice, not to measure research use.
Scott, W., & Marfell-Jones, M. (2004). Evidence alone is not enough to bring about practice change. Nursing New Zealand, 10(1), 14-16.	Purpose is to measure use of specific practice, not to measure research use.

Citation	Exclusion reason
Stetler, C. B., Corrigan, B., SanderBuscemi, K., & Burns, M. (1999). Integration of evidence into practice and the change process: Fall prevention program as a model. Outcomes Management for Nursing Practice, 3(3), 102-111.	Purpose is to measure use of specific practice, not to measure research use.
Stevenson, K., Lewis, M., & Hay, E. (2006). Does physiotherapy management of low back pain change as a result of an evidence-based educational programme? Journal of Evaluation in Clinical Practice, 12(3), 365-375.	Purpose is to measure use of specific practice, not to measure research use.
Valanis, B., Labuhn, K. T., Stevens, N. H., Lichtenstein, E., & Brody, K. K. (2003). Integrating prenatal-postnatal smoking interventions into usual care in a health maintenance organization. Health Promotion Practice, 4(3), 236-248.	Purpose is to measure use of specific practice, not to measure research use.
Varney, Carolynne. Tu, Jack V., Institute for Clinical Evaluative Sciences in Ontario, & Canadian Cardiovascular Outcomes Research Team. (2004). Quality of cardiac care in Ontario: EFFECT (enhanced feedback for effective cardiac treatment). phase 1 report.	Purpose is to measure use of specific practice, not to measure research use.
Watters, C. A. (2007). Nutrition evidence in practice: How heart health promotion and guidelines are used by dietitians and regional health authorities. (University of Alberta, Canada).	Purpose is to measure use of specific practice, not to measure research use.
White, C. L. (1999). Changing pain management practice and impacting on patient outcomes. Clinical Nurse Specialist, 13(4), 166-172.	Purpose is to measure use of specific practice, not to measure research use.
Winter, J. C. (1990). Relationship between sources of knowledge and use of research findings. Journal of Continuing Education in Nursing, 21(3), 138-140.	Purpose is to measure use of specific practice, not to measure research use.
Wolanczyk, T., Moskwa, M., Gniadek, E., & Komender, J. (1999). Psychopharmacological preferences of Polish child and adolescent psychiatrists. European Child & Adolescent Psychiatry, 8(4), 320-324.	Purpose is to measure use of specific practice, not to measure research use.
Wygnanski-Jaffe, T. (2005). The effect on pediatric ophthalmologists of the randomized trial of patching regimens for treatment of moderate amblyopia. Journal of Aapos: American Association for Pediatric Ophthalmology & Strabismus, 9(3), 208-211.	Purpose is to measure use of specific practice, not to measure research use.
Young, W. W., Marks, S. M., Kohler, S. A., & Hsu, A. Y. (1996). Dissemination of clinical results. Mastectomy versus lumpectomy and radiation therapy. Medical Care, 34(10), 1003-1017.	Purpose is to measure use of specific practice, not to measure research use.
Zwart-van Rijkom, Leufkens, H. G. M., Simoons, M. L., & Broekmans, A. W. (2002). Variability in abciximab (ReoPro (R)) prescribing: Evidence based or budget driven? Pharmacoepidemiology and drug safety, 11(2), 135-141.	Purpose is to measure use of specific practice, not to measure research use.
Goode, C. J., Lovett, M. K., Hayes, J. E., & Butcher, L. A. (1987). Use of research based knowledge in clinical practice. Journal of Nursing Administration, 17(12), 11-18.	Purpose is to measure use of specific practice, not to measure research use.

Citation	Exclusion reason
Heiberger, G. L. (2002). Factors affecting the health care beliefs, attitudes and caregiving behaviors of pediatric nurse practitioners: A case study of change. Rutgers The State University of New Jersey - New Brunswick.	Purpose is to measure use of specific practice, not to measure research use.<br><br>Is a report on factors related to research use without measuring research use.
Davies, S. (1999). Occasional paper: Practice nurses' use of evidence-based research. Nursing Times, 95(4), 57-60.	Purpose is to measure use of specific practice, not to measure research use.
Ketefian, S. (1975) Application of selected nursing research findings into nursing practice: A pilot study. Nursing Research, 24(2) 89-92.	Purpose is to measure use of specific practice, not to measure research use.
Farruggia, M. (2003). A case study of the "Gourmet Education" situated learning model for teaching and learning research in the nursing profession. University of Idaho.	Purpose is to measure use of specific practice, not to measure research use.
Shibata, M. C., Soneff, C. M., & Tsuyuki, R. T. (2005). Utilization of evidence-based therapies for heart failure in the institutionalized elderly. European Journal of Heart Failure, 7(7), 1122-1125.	Purpose is to measure use of specific practice, not to measure research use.
Wolgin (1996). Perspectives on research. Practice changes through research utilization. Journal of Nursing Staff Development, 12(4), 219-220.	Purpose is to measure use of specific practice, not to measure research use.
Williamson, J. W. et al. (1989) Health science information management and continuing education of physicians. Annals of Internal Medicin, 110, 151-160.	Sources of knowledge. Not an instrument to measure research use.
Winters, C. A., Besel, J., Dea, J. E., III, Jorgensen, K. P., & Lee, H. J. (2006). Understanding health research utilization in rural settings: Research use & access: Interviews with practicing rural nurses in Montana. Communicating Nursing Research, 39, 167-167.	Sources of knowledge. Not an instrument to measure research use.
Glover, P. (2000). The journal has something for everyone: Journal reading habits of midwives who are members of the Australian College of Midwives: A national survey. Australian College of Midwives Journal, 13(3), 26-30.	Sources of knowledge. Not an instrument to measure research use. Not a development or use of research use report. Is a report on factors related to research use without measuring research use.
Amin, M., Saunders, J. A., & Fenton, J. E. (2007). Pilot study of the knowledge and attitude towards evidence based medicine of otolaryngology higher surgical trainees. Clinical Otolaryngology, 32(2), 133-135.	Sources of knowledge. Report on related factors but not on measure of research use.

Citation	Exclusion reason
Akindipe, T. A., & Guidon, M. (2008). Evidence based practice: attitudes, use, and knowledge of physiotherapists in the Republic of Ireland: Rehabilitation and Therapy Research Society Fourth Annual Conference. Physical Therapy Reviews, 13(3), 198-199.	Sources of knowledge. No research use measure. Assesses sources and attitudes.
Amin, F. A., Fedorowicz, Z., Montgomery, A. J.(2006). A study of knowledge and attitudes towards the use of evidence-based medicine among primary health care physicians in Bahrain. Saudi Medical Journal, 27(9), 1394-1396.	Sources of knowledge. Not on development or use of research, report on related factors but not on measure of research use.
Baessler, C. A., et al. (1994). Medical-surgical nurses' utilization of research methods and products. Medsurg nursing : Official journal of the Academy of Medical-Surgical Nurses, 3(2), 113-117, 120.	Sources of knowledge.
Erickson, B. A. (1988). Method for incorporating nursing research findings into critical care nursing practice. Union for experimenting colleges and universities.	Sources of knowledge.
Estabooks (1998). Research Utilization Questionnaire--"modified" (1998). Will evidence-based nursing practice make practice perfect? Canadian Journal of Nursing Research, 30, 15-36.	Sources of Knowledge.
Glenton, C., Oxman, A. D., & Oxman, A. (1998). The use of evidence by health care user organizations. Health Expectations, 1(1), 14-22.	Sources of knowledge.
O'Donnell, C. A. (2004). Attitudes and knowledge of primary care professionals towards evidence-based practice: A postal survey. Journal of Evaluation in Clinical Practice, 10(2), 197-205.	Sources of knowledge.
Profetto-McGrath, J., et al. (2007). Clinical nurse specialists' use of evidence in practice: a pilot study. Worldviews on Evidence-Based Nursing, 4(2), 86-96.	Sources of knowledge.
Sackett, D. L., Straus, S. E., & Firm, A. N. (1998). Finding and applying evidence during clinical rounds - The "evidence cart". Journal of the American Medical Association, 280(15), 1336-1338.	Sources of knowledge.
Salisbury, C., Bosanquet, N., Wilkinson, E., Bosanquet, A., & Hasler, J. (1998). The implementation of evidence-based medicine in general practice prescribing. British Journal of General Practice, 48(437), 1849-1852.	Sources of knowledge.
Turner, P. (2001). Evidence-based practice and physiotherapy in the 1990s. Physiotherapy Theory and Practice, 17(2), 107-121.	Sources of knowledge.
Gerrish, K., & Clayton, J. (2004). Promoting evidence-based practice: An organizational approach. Journal of Nursing Management, 12(2), 114-123.	Sources of knowledge & barriers.

Citation	Exclusion reason
An investigation of the attitudes towards, and implementation of evidence based practice in physiotherapy in Ireland (2006). Physiotherapy Ireland, 27(2), 33-34.	Sources of knowledge (see Jette 2003). Not a research report; is an outline of a student project.
Schaafsma, F., et al. (2007). Enhancing evidence-based advice of occupational health physicians. Scandinavian Journal of Work, Environment & Health, 33(5), 368-378.	Sources of knowledge and search skills.
Caldwell, K., et al. (2007). Preparing for professional practice: How well does professional training equip health and social care practitioners to engage in evidence-based practice? Nurse Education Today, 27(6), 518-528.	Sources of knowledge. Not on development or use of research, report on related factors but not on measure of research use.
Nieri, M., & Mauro, S. (2008). Continuing professional development of dental practitioners in Prato, Italy. Journal of Dental Education, 72(5), 616-625.	Sources of knowledge.
Barnieh, L., & Edge, D. S. (2006). Understanding health research utilization in rural settings: Cross-border collaborative research: pilot questionnaire results from Montana. Communicating Nursing Research, 39, 168-168. **Published abstract for Winters 2007 study**	Sources of knowledge.
Gerrish, K., Ashworth, P., Lacey, A., & Bailey, J. (2008). Developing evidence-based practice: Experiences of senior and junior clinical nurses. Journal of Advanced Nursing, 62(1), 62-73.	Sources of knowledge.
Weiss, R., et al. (1990). Changing patient management: What influences the practicing pediatrician? Pediatrics, 85(5), 791-795.	Sources of knowledge. No instrument to measure research use.
Turner, P., & Whitfield, T. W. A. (1997). Physiotherapists' use of evidence based practice: A cross-national study. Physiotherapy Research International, 2(1), 17-29.	Sources of knowledge. Not an instrument to measure research use. Not a development or use of research use measure. Reports on factors related to research use without reporting on measure of research use.
Winters, C. A., et al. (2007). Access to and use of research by rural nurses. Rural & Remote Health, 7(3), 758.	Sources of knowledge. Not an instrument to measure research use. Not a development or use of research use measure. Reports on factors related to research use without reporting on measure of research use.

Citation	Exclusion reason
Stross, J. K. (1987). Information sources and clinical decisions. Journal of General Internal Medicine, 2(3), 155-159.	Sources of knowledge. Not research use measure. Criteria for appropriate use (of diagnostic/ therapeutic modalities) were based on expert opinion. Remainder of data is about sources of knowledge.
Estabrooks, C. A., Chong, H., Brigidear, K., & Profetto-McGrath, J. (2005). Profiling Canadian nurses' preferred knowledge sources for clinical practice. Canadian Journal of Nursing Research, 37(2), 118-140.	Sources of knowledge. No research use measure.
Hickie, S., Ross, S., & Bond, C. (1998). A survey of the management of leg ulcers in primary care settings in Scotland. Journal of Clinical Nursing, 7(1), 45-50.	Sources of knowledge. Not research use measure. Instrument measures current practice.
Bogdan-Lovis, E. A., & Sousa, A. (2006). The contextual influence of professional culture: Certified nurse-midwives' knowledge of and reliance on evidence-based practice. Social Science & Medicine, 62(11), 2681-2693.	Sources of knowledge. Not on development or use of research, report on related factors but not on measure of research use.
Burkiewicz, J. S., Zgarrick, D. P. (2005). Evidence-based practice by pharmacists: Utilization and barriers. Annals of Pharmacotherapy, 39(7-8), 1214-1219.	Sources of knowledge. Not on development or use of research, report on related factors but not on measure of research use.
James, E. L., et al. (2007). Use of research by the Australian health promotion workforce. Health Education Research, 22(4), 576-587.	Sources of knowledge. Not on development or use of research use. Reports on factors related to research use without reporting on measure of research use.
Forsetlund, L., Bradley, P., Forsen, L., Nordheim, L., Jamtvedt, G., & Bjørndal, A. (2003). Randomised controlled trial of a theoretically grounded tailored intervention to diffuse evidence-based public health practice. BMC Medical Education, 3, 1-12.	Sources of knowledge. Not on development or use of research use. Reports on factors related to research use without reporting on measure of research use.
Coleman, P., & Nicholl, J. (2001). Influence of evidence-based guidance on health policy and clinical practice in England. Quality in Health Care, 10(4), 229-237.	Sources of knowledge. Not on development or use of research, report on related factors but not on measure of research use.

Citation	Exclusion reason
Mattila, L. M., Koivisto, V., & Haggman-Laitila, A. (2005). Evaluation of learning outcomes in a research process and the utilization of research knowledge from the viewpoint of nursing students. Nurse Education Today, 25(6), 487-495.	Sources of knowledge. Reports on factors related to research use (with nursing students) without reporting on measure of research use.
Philibert, D. B., et al. (2003). Practitioners' reading patterns, attitudes, and use of research reported in occupational therapy journals. American Journal of Occupational Therapy, 57(4), 450-458.	Sources of knowledge. Operationalized more as use of a source of knowledge.
Banning, M. (2005). Conceptions of evidence, evidence-based medicine, evidence-based practice and their use in nursing: Independent nurse prescribers' views. Journal of Clinical Nursing, 14(4), 411-417.	Sources of knowledge. Reports on EBP terms.
Jette, D. U., et al. (2003). Evidence-based practice: Beliefs, attitudes, knowledge, and behaviors of physical therapists. Physical Therapy, 83(9), 786-805.	Sources of knowledge. Reports on factors related to research use without reporting on measure of research use.
Zipoli, R. P., Jr. (2004). Evidence-based practice among speech-language pathologists: Attitudes, utilization, and barriers. Southern Connecticut State University, United States -- Connecticut.	Sources of knowledge. Research use defined as use of sources; barriers to EBP.
Pierce, S. T. (2000). Readiness for evidence-based practice: Information literacy needs of nursing faculty and students in a Southern United States state. Northwestern State University of Louisiana.	Sources of knowledge; EBP Process.
Zipoli, R. P., Jr., Kennedy, M. (2005). Evidence-based practice among speech-language pathologists: Attitudes, utilization, and barriers. American Journal of Speech-Language Pathology, 14(3), 208-220.	Sources of knowledge; research use defined as use of sources. Barriers to EBP.
Bauer, I., Lechner, S., & Wojciech, J. (2007). Evidence-based practice in physiotherapy: The current situation in Germany as compared to England (part 2). Zeitschrift fuer Physiotherapeuten, 59(2), 122-137.	Sources of knowledge; research use defined as use of sources. Barriers to EBP.
Egerod, I. (2004). Survey of evidence-based practice among critical care nurses in Denmark. CONNECT: The World of Critical Care Nursing, 3(2), 38-42.	Sources of knowledge; research use defined as use of sources. Barriers to EBP.
Estabrooks, C. A. Translating research into practice: Implications for organizations and administrators. Can J Nurs Res. 2003 Sep;35(3):53-68.	Sources of knowledge; research use defined as use of sources. Barriers to EBP.
