# Supplementary material for: A systematic review of the psychometric properties of self-report research utilization measures used in healthcare
Source: Implement Sci. 2011 Jul 27;6:83. doi: 10.1186/1748-5908-6-83 (PMC3169486; doi:10.1186/1748-5908-6-83)
Supplement: Additional file 3 — The Standards. This file contains an overview of the Standards for Educational and Psychological Testing Validity Framework and sample predictions used to assess 'relations to other variables' validity evidence according to this framework. [file 1748-5908-6-83-S3.PDF]

## Additional File 3. The *Standards*

## Overview of the *Standards for Educational and Psychological Testing* Validity Framework

Reliability, in the *Standards*, is described as the consistency of measurement when a testing procedure is repeated on a population of individuals or groups [1]. The *Standards* further suggests that critical information on reliability includes: the identification of major sources of measurement error; summary statistics bearing on the size of such errors; and the degree of generalizability of scores across alternate forms, administrations, or other relevant dimensions [1]. Reliability information may be reported in terms of variances or standard deviations of measurement errors, in terms of item response theory test/measure information functions, or more commonly, in terms of one or more coefficients. Three categories of reliability coefficients are traditionally reported: (1) coefficients based on the correlations between scores obtained from individual items or subsets of items in a measure accruing from a single administration (i.e., internal consistency – Cronbach's Alpha), (2) coefficients obtained from administrating a measure to the same individuals on two separate occasions (i.e., test-retest reliability), and (3) coefficients derived from the administration of parallel forms of a measure in independent testing situations (i.e., alternate-form coefficients) [1].

Validity, in the *Standards*, is defined as "the degree to which evidence and theory support the interpretations of test scores entailed by proposed uses of tests" [1] (p. 9). Thus, it is the scores obtained from the administration of a self-report measure and the interpretations made based on these scores that are validated, not the measure itself. The *Standards* present a contemporary conceptualization of validity. In this approach, validity is thought of as a unitary concept (not as distinct types) where all evidence sources

contribute equally to construct validity. Therefore, all study results (not just those labeled as validity) are scrutinized in terms of whether or not they add to a construct validity argument. Four sources of validity evidence are outlined in the *Standards*: (1) content, (2) response processes, (3) internal structure, and (4) relations to other variables.

*Content evidence* refers to the extent to which the items in a self-report measure adequately represent the content domain of the concept or construct of interest [1, 2]. This validity source is relevant to all measures regardless of content domain (cognitive or affective) and format (paper-and-pencil, online, observation schedule, interview protocol, etc.). Published literature and experts' evaluations are key approaches for obtaining content validity evidence.

*Response processes evidence* refers to how respondents interpret, process, and elaborate upon item content and whether this behaviour is in accordance with the concept or construct being measured [1]. This validity source can provide evidence on: (1) the fit between the concept or construct of interest and the nature of the response given by the respondents; (2) and any differences in meaning or interpretation of scores across subgroups of respondents. Interviews with, and observation of respondents while engaging in the concept or construct under evaluation, as well as pilot tests and feasibility work, provide data of this type [3].

*Internal structure evidence* examines the relationships between the items on a self-report measure to evaluate its dimensionality [1]. This validity source attempts to answer the question, "To what extent do the relationships among items match the concept or construct as operationally defined"? and provide a rationale for combining scores obtained on individual items to produce a derived score. Factor analysis (exploratory and

confirmatory) and item-total statistics (e.g., item total correlations, scale alpha when an item is deleted) are commonly used to provide internal structure validity evidence.

*Relations to other variables evidence* provide the fourth source of validity evidence. External variables may include measures of criteria that the concept or construct of interest is expected to predict, as well as relationships to other scales hypothesized to measure the same concepts or constructs, and variables measuring related or different concepts or constructs [1]. This type of evidence is most often reported using bivariate correlations, predictive statistical models, and multi-group-comparisons. In our review, to assess relations to other variables, we *a priori* (based on commonly used research utilization theories and systematic reviews) identified established relationships between research utilization and other (external) variables. Tables 1 and 2, presented next, contain a sample of these predictions.

## Table 1. Sample Predictions Based on Research Utilization Theory

| **Author**<br><br>**[Citation Number]** | **Theory** | **Assumptions** | **Comments** |
|---|---|---|---|
| Rogers EM. 1983/2005 Diffusion of Innovations. [4, 5] | Diffusion of Innovation | • **Innovation-decision process: the stages of the innovation-adoption process include knowledge (or awareness), persuasion, decision, use (or implementation), and confirmation. These stages may progress in a linear manner, but not always.**<br><br>Other assumptions that may come into play in articles utilizing Rogers' framework are:<br><br>• Individual innovativeness: A bell shaped curve illustrates the percentage of who (innovators, early adopters, early majority, late majority, laggards) adopts the innovation in relation to a given time frame.<br><br>• Rate of Adoption: An s-curve on a graph best represents the rate of adoption of innovations, occurring slowly at first, followed by a period of rapid growth that will taper off, stabilize, and eventually decline.<br><br>• Perceived attributes of the innovation contributing to adoption include: relative advantage over the status quo, compatibility with values/current practices, complexity, trialability, and observability of results. (Rogers discusses the following characteristics thought to influence innovation diffusion and adoption. NOTE: remember this theory was developed in the context of agriculture innovation diffusion.<br><br><u>Innovation</u><br><br>• Complexity – the degree to which an innovation is perceived as relatively difficult to understand and use (higher complexity is associated with lower utilization)<br><br>• Relative advantage/research relevance – | Theory has been applied and tested repeatedly both within and outside of nursing<br><br>Has demonstrated longevity, within a number of disciplines.<br><br>Has not been proven that innovation = research |

| Author<br><br>[Citation Number] | Theory | Assumptions | Comments |
| --- | --- | --- | --- |
| | | the degree to which an innovation is perceived as being better than what it replaces (more relative advantage, the more utilization)<br><br>• Compatibility – the degree to which an innovation is perceived as consistent with values, experiences, and need (more compatible, the more utilization)<br><br>• Trialability – the degree to which an innovation may be experimented with on a limited basis (more trialable an innovation, the more utilization)<br><br>• Observability – the degree to which the results of implementing an innovation are visible (more observable, the more utilization)<br><br>• Additional factors that influence innovation behaviour include: support for innovation; mass media; people/co-worker support<br><br><u>Individual</u><br><br>• Socioeconomic characteristics: more education, income/social status, literacy, age (findings are inconclusive on this point)<br><br>• Adoptiveness<br><br>• Personality variables (empathy, dogmatism, ability to deal with abstractions, rationality, intelligence, positive change attitude, ability to cope with uncertainty, favourable attitude towards education, optimism, high levels of achievement motivation)<br><br>• Communication behaviour (social participation, interconnected social systems, cosmopoliteness, change agent contact, contact with | |

| Author<br><br>[Citation Number] | Theory | Assumptions | Comments |
|---|---|---|---|
| | | interpersonal communication channels, knowledge of innovations, high levels of opinion leadership, part of highly interconnected systems, localite) | |
| Logan J, Graham I. 1998 Toward a comprehensive interdisciplinary model of health care research use. [6] | Ottawa Model of Research Use | • There are three sources for the barriers and supports to research use. These relate to the practice environment (consisting of structural factors, social factors, and patients), the potential adopters, and the evidence-based innovation.<br><br>• Viewing the proposed change from the perspective of the adopters and understanding scientific and extra-scientific considerations may influence adoption<br><br>• The attributes of an innovation interact with potential adopters. Those thought to positively influence adoption include credible developers and involvement of potential adopters in the process, clear translation processes (i.e. rigorous literature searching and incorporation of objective methods to synthesize the evidence), compatibility, relative advantage, low complexity, and high trialability<br><br>• While evidence of the efficacy of various transfer strategies is limited, in general, the use of multiple strategies appears to be more effective. This model suggests that tailoring strategies to the barriers/supports of the particular setting may enhance implementation of research. | Model refined through discussions with participants<br><br>Constructs are supported by evidence (where available)<br><br>Authors state that the model is based on the literature, but method for identifying literature in this area is not discussed.<br><br>Report study findings to support model components but quality assessment of studies was not undertaken |
| Kitson et al., 1998. Enabling the implementation of evidence based practice: A conceptual framework. [7] | PARiHS<br><br>Promoting Action on Research in Health Services | **2 key assumptions**<br><br>1. research implementation is not a linear process<br><br>2. implementation of quality research will | While this is a fairly new framework, there have been some empirical reports supporting its propositions |

| Author<br><br>[Citation Number] | Theory | Assumptions | Comments |
|---|---|---|---|
| Rycroft-Malone, J (2004). The PARIIHS framework—A framework for guiding the implementation of evidence-based practice. [8] | | result in improved patient outcomes<br><br>Successful research implementation is the result of interplay between:<br><br>1. evidence (research, clinical experience, patient preferences)<br><br>2. context (culture, leadership, and evaluation, and resources)<br><br>3. facilitation (process of enabling).<br><br>Structural resources include things like access to nursing journals and libraries | |
| Godin G et al., 2008. Healthcare professionals' intentions and behaviours: A systematic review of studies based on social cognitive theories. [9]<br><br>Sheeran P 2002. Intention-behavior relations: A conceptual and empirical review. [10] | TPB<br><br>Theory of Planned Behavior | TPB posits that individual behaviour (use of research) is driven by behavioural intentions where behavioural intentions are a function of:<br><br>(1) Behavioural Beliefs, i.e., beliefs about the likely outcomes of the behaviour and the evaluations of these outcomes<br><br>→ Behavioural beliefs produce a favourable or unfavourable ***attitude*** toward the behaviour<br><br>(2) Normative Beliefs, i.e., beliefs about the normative expectations of others and motivation to comply with these expectations<br><br>→ Normative beliefs result in perceived social pressure or ***subjective norm***<br><br>(3) Control Beliefs, i.e., beliefs about the presence of factors that may facilitate or impede performance of the behaviour<br><br>→ Control beliefs give rise to ***perceived behavioural control*** | This theory has been used extensively by physicians (in particular in the examination of prescribing behaviour). However, one of the main premises is that the greatest predictor of behaviour is intention and in recent reviews, on average, approximately only 30% of the variance in behaviour is accounted for by intentions |
| Grol R, et al., 2007. Planning and studying improvement in patient care: The use of theoretical perspectives. [11] | Multiple Theories | • There is a list of ‘innovation characteristics that may promote or hinder their implementation’ on p. 100- the scientific basis for these principles, they state, lies in organizational research with little investigation in healthcare: | **Predictions are based on commonalities between a variety of theories**<br><br>**Note: Factors hypothesized to** |

| Author<br><br>[Citation Number] | Theory | Assumptions | Comments |
| --- | --- | --- | --- |
| | | 1. relative advantage or utility<br>2. compatibility<br>3. complexity<br>4. costs<br>5. risks<br>6. flexibility, adaptability<br>7. involvement<br>8. divisibility<br>9. trialability, reversibility<br>10. visibility, observability<br>11. centrality<br>12. pervasiveness, scope, impact<br>13. magnitude, disruptiveness, radicalness<br>14. duration<br>15. form, physical properties<br>16. collective action<br>17. presentation | **affect ‘implementation of change’**<br><br>Combined search methods (databases, hand-searching, and expert review). Database search from 2000-2002. |

## Table 2. Sample Predictions Based on Empirical Evidence (Reviews)

| **Author**<br><br>**[Citation Number]** | **Dependent Variable** | **Unit of Analysis** | **Significant External Variable(s)** | **Direction of Effect** | **Quality**<br><br>(Oxman QA checklist) |
|---|---|---|---|---|---|
| | | **External Variables: Individual Characteristics** | | | |
| Estabrooks CA, et al. 2003. Individual determinants of research utilization: A systematic review. [12] | **Research Utilization**<br><br>*Not defined* | **Individual**<br><br>*Nurses* | • Attitude towards research | Positive | 6/7<br><br>'Minimal Flaws' |
| Squires JE et al. 2011. Individual determinants of research utilization: A systematic review update.[13] | **Research Utilization**<br><br>*"that process by which specific research-based knowledge (science) is implemented in practice"*<br><br>**Kinds of Research Utilization**<br><br>*"Instrumental research utilization refers to the concrete application of research findings in clinical practice."*<br><br>*"Conceptual research utilization refers to the cognitive use of research where the research may be used to change one's thinking about* | | • Attitude towards research<br>• Attending in-services/confere nce<br>• Role (leadership/ advanced practice compared to staff nurse)<br>• Education (graduate degree compared to bachelors/ diploma)<br>• Clinical specialty (specialty unit compared to general hospital ward)<br>• Job satisfaction | | No QA as this review is forthcoming |

| **Author**<br><br>**[Citation Number]** | **Dependent Variable** | **Unit of Analysis** | **Significant External Variable(s)** | **Direction of Effect** | **Quality**<br><br>(Oxman QA checklist) |
|---|---|---|---|---|---|
| | *a specific practice, but may or may not result in a change in action."*<br><br>*"Symbolic research utilization is the use of research as a persuasive or political tool to legitimate a position or influence the practice of others."*<br><br>*"Overall research utilization refers to the use of any kind of research in any way in practice."* | | | | |
| **External Variables: Contextual/Organizational Characteristics** | | | | | |
| Meijers JMM, et al. 2006. Assessing the relationships between contextual factors and research utilization in nursing: A systematic literature review. [14] | **Research Utilization**<br><br>*"indirect (using research to influence thinking at a general level) and direct (the application of research in clinical practice)"* | **Individual**<br><br>*Nurses working in clinical practice* | • Role (sustained participation in QI work; 1 study) | Positive | 5/7<br><br>'Minor Flaws' |
| Greenhalgh T, et al ., 2004. Diffusion of innovations in service organizations: Systematic review | **Innovation Adoption**<br><br>*Innovation in service delivery and organization: "a novel set of* | **Organization**<br><br>*Service sector, focused on health care* | • Size (larger)<br>• Maturity<br>• Organizational complexity<br>• Functionally differentiated: presence of | Positive<br><br>(Strong-direct; Consistent findings in two or more empirical | 5/7<br><br>'Minor Flaws' |

| Author<br><br>[Citation Number] | Dependent Variable | Unit of Analysis | Significant External Variable(s) | Direction of Effect | Quality<br><br>(Oxman QA checklist) |
|---|---|---|---|---|---|
| and recommendations. [15] | *behaviours, routines, and ways of working that are directed at improving health outcomes, administrative efficiency, cost effectiveness, or users' experience and that are implemented by planned and coordinated actions".* | | semi-autonomous departments/units<br>• Specialized: foci of professional knowledge<br>• Organizational slack: access to and amount of resources, slack resources to channel into new projects<br>• Centralization: De-centralized decision making structures, professional autonomy<br>• Absorptive capacity for new knowledge: Encompasses the organizations existing knowledge and skills, pre-existing related technologies, a 'learning organization' culture, and proactive leadership toward sharing knowledge | studies of appropriate design and high scientific quality undertaken in health service organizations) | |
| **External Variables: Interventions*** | | | | | |
| Thompson DS, et al. 2007. Interventions aimed at increasing research use in nursing: A systematic review. [16] | **Research Use**<br><br>*Instrumental: "the concrete application of research to practice"*<br><br>*Conceptual: "use of research to* | **Individual and Unit**<br><br>*Nurses* | • Multidisciplinary committees (1 study; low quality)<br>• Local opinion leaders (1 study; low quality) | Positive | 7/7<br><br>'Minimal Flaws' |

| Author<br><br>[Citation Number] | Dependent Variable | Unit of Analysis | Significant External Variable(s) | Direction of Effect | Quality<br><br>(Oxman QA checklist) |
|---|---|---|---|---|---|
| | *change one's thinking but not necessarily one's action"*<br><br>*Symbolic:" use of research to influence policies or decisions"* | | | | |
| Grimshaw JM, et al. 2004. Effectiveness and efficiency of guideline dissemination and implementation strategies. [17] | **Guideline Adherence**<br><br>*Guideline: "systematically developed statements to assist practitioner decisions about appropriate health care for specific clinical circumstances"* | **Individual**<br><br>*Medically qualified healthcare professionals* | Median absolute improvement in performance for clustered randomized comparisons of the following interventions:<br><br>• Reminders (14 comparisons) = 14.1%<br>• Educational materials (4 comparisons) = 8.1%<br>• Audit and feedback (5 comparisons) = 7.0%<br>• Multifaceted interventions involving educational outreach (13 comparisons) = 6.0% | Median absolute improvement in performance | 6/7<br><br>'Minimal Flaws' |
| Grol R, et al. 1999. Evidence-based implementation of evidence-based medicine. [18] | **Guideline Adherence**<br><br>*Improvement in performance related to guideline implementation.* | **Individual**<br><br>*Health care professionals; not specified* | • Reminders (unless they are used for routine items of care or if too many prompts are presented at the same time)<br>• Introduction of other computer information systems<br>• Educational outreach or | Reported as 'generally effective' | 3/7 'Major flaws' |

| Author<br><br>[Citation Number] | Dependent Variable | Unit of Analysis | Significant External Variable(s) | Direction of Effect | Quality<br><br>(Oxman QA checklist) |
|---|---|---|---|---|---|
| | | | academic detailing for prescribing decisions<br>• Tailored interventions based on assessment of barriers<br>• Multifaceted interventions | | |

*Interventions were included as an 'external variable' to ensure our assessment of relations between research use and external variables was as comprehensive as possible and because interventions to increase research use are often modeled as predictors of research use.

## References


1. American Educational Research Association, American Psychological Association, National Council on Measurement in Education: **Standards for Educational and Psychological Testing**. Washington, D.C.: American Educational Research Association; 1999.
2. Waltz C, Strickland OL, Lenz E, R: **Measurement in Nursing and Health Research**, third edn. New York: Springer Publishing Company; 2005.
3. Tourangeau R, Rips L, Rasinski K: **The Psychology of the Survey Response**. New York: Cambridge University Press; 2000.
4. Rogers EM: **Diffusion of Innovations**, 3rd edn. New York: The Free Press; 1983.
5. Rogers E: **Diffusion of Innovations**, 4th edn. New York: The Free Press; 1995.
6. Logan J, Graham ID: **Toward a comprehensive interdisciplinary model of health care research use**. *Science Communication* 1998, **20**(2):227-246.
7. Kitson A, Harvey G, McCormack B: **Enabling the implementation of evidence based practice: a conceptual framework**. *Quality in Health Care* 1998, **7**(3):149-158.
8. Rycroft-Malone J: **The PARIHS framework - A framework for guiding the implementation of evidence-based practice**. *Journal of Nursing Care Quality* 2004, **19**:297-304.
9. Godin G, Belanger-Gravel A, Eccles M, Grimshaw G: **Healthcare professionals' intentions and behaviours: A systematic review of studies based on social cognitive theories**. *Implementation Science* 2008, **3**(36).
10. Sheeran P: **Intention-behaviour relations: A conceptual and empirical review**. *Eur Rev Soc* 2002, **12**:1-36.
11. Grol RPTM, Bosch MC, Hulscher MEJL, Eccles MP, Wensing M: **Planning and studying improvement in patient care: The use of theoretical perspectives**. *Milbank Quarterly* 2007, **85**(1):93-138.
12. Estabrooks CA, Floyd JA, Scott-Findlay S, O'Leary KA, Gushta M: **Individual determinants of research utilization: A systematic review**. *Journal of Advanced Nursing* 2003, **43**(5):506-520.
13. Squires J, Estabrooks C, Gustavsson P, Wallin L: **Individual determinants of research utilization by nurses: A systematic review update**. *Implementation Science* 2011, **6**(1).
14. Meijers JMM, Janssen MAP, Cummings GG, Wallin L, Estabrooks CA, Halfens RYG: **Assessing the relationships between contextual factors and research utilization in nursing: systematic literature review**. *Journal of Advanced Nursing* 2006, **55**(5):622-635.
15. Greenhalgh T, Robert G, Macfarlane F, Bate P, Kyriakidou O: **Diffusion of innovation in service organizations:Systematic review and recommendations** *Milbank Q* 2004, **82**(4):581-629.

16. Thompson DS, Estabrooks CA, Scott-Findlay S, Moore K, Wallin L, Thompson DS, Estabrooks CA, Scott-Findlay S, Moore K, Wallin L: **Interventions aimed at increasing research use in nursing: a systematic review**. *Implementation Science* 2007, **2**:15.
17. Grimshaw JM, Thomas RE, MacIennan G, Fraser CR, Vale L, Whity P, Eccles MP, Matowe L, Shirran L, Wensing M *et al*: **Effectiveness and efficiency of guideline dissemination and implementation strategies**. *Health Technology Assessment* 2004, **8**(6):1-72.
18. Grol R, Grimshaw J: **Evidence-based implementation of evidence-based medicine**. *Journal on Quality Improvement* 1999, **25**(10):503-513.
