# Supplementary material for: A systematic review of the psychometric properties of self-report research utilization measures used in healthcare
Source: Implement Sci. 2011 Jul 27;6:83. doi: 10.1186/1748-5908-6-83 (PMC3169486; doi:10.1186/1748-5908-6-83)
Supplement: Additional file 4 — Description of Other Specific Practices Indices and Other General Research Use Indices. This file contains a description of the four measures included in the class 'Other Specific Practices Indices' and the ten measures included in the class 'Other General Research Use Indices'. [file 1748-5908-6-83-S4.PDF]

Additional File 4. ***Description of Other Specific Practices Indices and Other General Research Use Indices***

Other Specific Practices Indices

First Author [citation in manuscript]	Description of research use measure
Aron [129]	20 items relating to the use of specific therapeutic techniques for depression. Sample items include:<br>• Establish and maintain rapport<br>• Be sincere and genuine<br>• Convey a warm and interested attitude<br><br>Scored on a 7-point Likert scale from 1=do not use at all to 7=use all the time. For analysis, scale points were combined to produce 3 separate categories: 1-2 (do not use), 3-5 (use sometimes), 6-7 (use all the time). A mean score based on the 20 practices was then calculated.
Knudsen [21]	Dependant variable was the organizational use of treatment innovations. Sample treatment innovations assessed include:<br>• Disulfiram<br>• Naltrexone<br>• Rapid opiate detoxification<br><br>Each item was scored dichotomously as yes/no. An aggregate measure of innovation adoption based on an additive index of the 15 innovative substance abuse treatment techniques was calculated.
Tita [17]<br>Tita [18]<br><br>[1 study, 2 articles]	Questionnaire designed to estimate awareness and use of 13 obstetrical interventions. Sample interventions include:<br>• Antenatal corticosteroids for impending prematurity<br>• Antiretrovirals to prevent mother-to-child transmission of HIV/AIDS<br>• Uterotonics to prevent postpartum hemorrhage<br><br>Each item was scored dichotomously as yes/no for awareness and use. An additive composite score based on the 13 interventions was then calculated.
Varcoe [50]	Use of 10 specific research practices. Sample practices include:<br>• IM injection<br>• Catheter removal<br>• Sensory information/ diagnostic<br><br>Each practice was scored on a 3 pt scale: never (1), sometimes (2), always (3) or ‘not applicable’. A mean score based on the ten practices was then calculated.

Other General Research Use Indices

Author, Year [Citation in manuscript	Description of research use measure
Forbes [101]	Research use index reflects the frequency with which staff nurses engaged in 5 research activities during the past year. Sample items include:<br>• Reviewing research literature applicable to their practice<br>• Rejecting or implementing a practice activity based on the results of a research study<br><br>Scored using the following four response options: 0, 1, 2-4, 5 or more times. Research use index score obtained by taking a sum of the 5 items.
Grasso [102]	Research use index composed of 9 items, which ask respondents how often they use research information in 9 different ways. Sample items include:<br>• Discussion with coworkers<br>• Discussion with students<br>• Assessing individual client change<br><br>Scored using a 4-point scale from 'never' to 'frequently'. Research use index score obtained by taking the sum of the 9 items.
Kamwendo [105]	Research use index composed of 4 statements measuring present engagement in research use. A sample item is:<br>• Apply research findings to improve physiotherapy practice<br><br>Each item is scored on a 5-point Likert scale from 'I do not agree at all' to 'I very much agree'. Item scores are then summed for an index score (4-20).
Karlsson [73]	Same index used as above (Kamwendo, 2002)
Morrow-Bradley [103]	Research use index composed of 6 items (called a 'research utility index'). Sample items include:<br>• Rate the degree to which the results from psychotherapy research have influenced the way you do psychotherapy<br>• Use of psychotherapy research in dealing with difficult treatment cases in the last year<br>• Practice changed by conferences in the last year<br><br>One item (rate the degree) scored using a 6-point scale from 'not at all' to 'more than any other factor'. Remainder of items scored dichotomously as yes/no. Method for combining items to form the index is not reported.
Pelz [84]	Research use index consisting of 10 items that measure the extent of research use directly or indirectly.<br><br>*Direct Measures of Research Use* sample items:<br>• Reviewed research literature in an effort to identify new knowledge for use in your practice<br>• Evaluated a research study to determine its value for practice

Author, Year [Citation in manuscript	Description of research use measure
	Scored using a 4-point scale from 0 to 5 or more times in the past year.<br><br>*Indirect Measures of Research Use* sample item:<br><br>• Extent to which several possible types of committees 'influence nursing practice in your hospital'<br><br>Scored using a 5-point scale from none to complete OR no extent to very great (depending on the item).<br><br>Research use index scores obtained by taking a mean of the 10 items.
Rardin [104]	Research use index measuring impact of psychotherapy research on practice with 3 items (this is within the section on research attitudes in the survey). Items are:<br><br>• Please estimate how much impact research in psychotherapy has on your actual practice of psychotherapy.<br>• Please estimate how much impact research in psychotherapy has on your thinking about the process of psychotherapy<br>• Please estimate how much impact research in psychotherapy has on your conceptualizations of clients/patients in psychotherapy<br><br>Scored on a 5-point Likert scale from 'very low' to 'very high'. Results from these items are then summed for an *Impact on Practice* rating ranging from 3-15.
Reynolds [24]	Research use index consists of 5 items focusing on the extent to which respondents participate in research activities. Sample items include:<br><br>• Reviewed research literature in an effort to identify new knowledge for use in your practice<br>• Evaluated a research study to determine its value for practice<br><br>Each item is asked with respect to the past year and is scored on a 4-point scale: 0, 1, 2-4, 5 or more times**.** Mean of the items are then taken as a measure of research utilization.
Stiefel [36]	Research use index consists of 18 items measuring respondents' reported participation in nursing research utilization activities. Sample items include:<br><br>• I read nursing research articles and learn about research-based nursing interventions<br>• I attend conferences/educational programs and learn about research-based nursing interventions<br>• I think about ways of using a research-based nursing intervention in my nursing practice when I become aware of it<br><br>Each item is scored on a 5-point scale from 'never' to 'always'. Item scores are then summed for an index score (18-90).
Varcoe [50]	Research use index contains 10 general statements on research use. Sample items include:<br><br>• Communicating concerns about the effectiveness of practices to colleagues

Author, Year [Citation in manuscript	Description of research use measure
	• Use of research articles to support questioning practice<br>• Identification of hospital policies based on research<br><br>Each item is scored on a 4-point scale from ‘not at all’ to ‘always’. Item scores are then summed for an index score (10-40).
