# Supplementary material for: A systematic review of the psychometric properties of self-report research utilization measures used in healthcare
Source: Implement Sci. 2011 Jul 27;6:83. doi: 10.1186/1748-5908-6-83 (PMC3169486; doi:10.1186/1748-5908-6-83)
Supplement: Additional file 5 — Reported Reliability of Self-Report Research Utilization Measures. This file contains the reliability coefficients reported in the included studies. [file 1748-5908-6-83-S5.PDF]

Additional File 5. ***Reported Reliability of Self-Report Research Utilization Measures (N=14 of 60 measures reported reliability evidence)***

Class	[citation number in manuscript]	Reliability		
		Internal consistency	Stability	Inter-rater
Nurses Practice Questionnaire	[33, 34]	α=0.95	r=0.83	
	[59]	α=0.74		
	[32]	α=0.68		
	[60]	α=0.91		
	[61]	α=0.85		
	[30, 31]	α=0.63		
	[62]	α=0.75		
	[63]	α=0.82		
	[35]	α=0.89	r=0.99	
Other Specific Practices Indices*	[50]	α=0.87		
Research Utilization Questionnaire	[55]	α=0.92		
	[118, 119]	α=0.88		
	[120]	α=0.84		
	[71]	α≥0.79		
	[79, 123]	α=0.93		
	[81]	α=0.92		
	[82]	α=0.86		
	[85]	α=0.94		
	[124]	α=0.93		
Edmonton Research Orientation Survey	[127]	α=0.89		
	[77, 78]	α=0.87		
	[76]	α=0.83		
Knott and Wildvasky	[20]	α=0.87		
Other General Research Utilization Indices*	[101]	α=0.78		
	[105]	α=0.73		
	[73]	α=0.80		
	[103]	α=0.73		
	[84]	α=0.87		
	[24]	α=0.86		
	[36]	α=0.94	r=0.88	
	[50]	α=0.87		
Other Single Items*	[37]			r=0.80 – 0.91

*each study represents a separate measure
