# Supplementary material for: A systematic review of the psychometric properties of self-report research utilization measures used in healthcare
Source: Implement Sci. 2011 Jul 27;6:83. doi: 10.1186/1748-5908-6-83 (PMC3169486; doi:10.1186/1748-5908-6-83)
Supplement: Additional file 6 — Supporting Validity Evidence by Self-Report Research Utilization Measure. This file contains the detailed validity evidence on each included self-report research utilization measure. [file 1748-5908-6-83-S6.PDF]

Additional File 6: Supporting Validity Evidence by Self-Report Research Utilization Measure

Study [citation in manuscript]	Content	Response Processes	Internal Structure	Relations with Other Variables
**NPQ n=11 articles (9 studies)**				
Brett<br>1987 [33]<br><br>**Index**	"Assumed" as practices were derived from published research using specific criteria developed by Haller et al. 1979	No evidence	Not reported	**Significant** ($p<0.05$)<br>• Nurses' perception about the existence of organizational policy and procedure (r=.626)<br>• Percentage of nurses with non-nursing master's degree<br>• (r=.133)<br><br>**Non-significant**<br>• Type or number of degrees [BN vs. diploma]
Brett<br>1989 [34]<br><br>*A report of Brett 1987*	"Assumed" as the practices were derived from published research reports using specific criteria developed by Haller et al. 1979	No evidence	Not reported	None reported
Barta<br>1995 [59]	Expert panel of 3 paediatric nurses active in paediatric pain assessment and management reviewed the research base for each practice.	No evidence	Not reported	None reported
Berggen<br>1996 [32]	Midwifery practices taken from doctoral dissertations and articles published in the journal of the Swedish Midwives' Association	No evidence	Not reported	**Non-significant**<br>• Experience<br>• Education (degree vs. diploma)
Coyle<br>1990 [60]	Appropriateness of the nursing practices; practices replicated in 1 study	No evidence	Not reported	**Significant** ($p<0.05$)<br>• Attendance at conferences (X^2=5.179, df=1)<br>• Policy perception: significant for 5/14 practices (Range r=0.50 to r=0.70) and significant overall (r=0.58).

Study [citation in manuscript]	Content	Response Processes	Internal Structure	Relations with Other Variables
				**Non-significant**<br>• Level of education<br>• Years experience in nursing
Michel<br>1995 [61]	"Assumed" as research findings derived from published nursing literature using specific criteria; replicated in at least 1 study	No evidence	Not reported	**Significant** ($p<0.05$)<br>• Educational level [bachelors vs masters]<br>• Awareness of agency policy (R^2=.18, F=16.26)<br><br>**Non-significant**<br>• Years of work experience
Rodgers<br>2000 [30]	Panel of nurse researchers and educators. *(Unclear if practice only evaluated or if items were evaluated as well.)*<br>14 practices and influencing factors were identified in the earlier exploratory study.	Validity of self-reporting levels of research utilization confirmed in pilot with 20 nurses.<br><br>Process and details of this are not reported.	Not reported	**Significant** ($p<0.05$)<br>• Whether ward gets nursing journals (z= -2.68)<br><br>**Non-significant**<br>• Time qualified<br>• Age
Rodgers<br>2000 [31]<br><br>*A report of Rodgers 2000a above*	Panel of nurse researchers and educators.<br>*(Unclear if practice only or if items were evaluated as well.)*<br><br>14 practices and influencing factors were identified in the earlier exploratory study	Ability of nurses to self-report open and honestly on their level of research utilization confirmed in pilot with 20 nurses. Option of 'not able to use' was added to survey but was coded as 'not using' for purpose of analysis.	Not reported	Progression through the stages of adoption was linear for the vast majority of nurses. Non-linear progression did occur for a small percentage.
Rutledge<br>1996 [62]	Expert panel: RU subcommittee and the clinical practice committee.<br>*(Unclear if practice only or if items were evaluated as well.)*	No evidence	Not reported	Progression through the stages of adoption was linear for the vast majority of nurses. Non-linear progression (i.e., persuasion or implementation without awareness) did occur for small percentage (<1%).<br><br>**Significant** ($p<0.05$)

Study [citation in manuscript]	Content	Response Processes	Internal Structure	Relations with Other Variables
				• Highest degree (diploma, degree, masters, doctorate) (r= -0.1205)<br>• Conferences attended in the last year (r=0.1168)
Squires 2007 [63]	"Assumed" as the research-based practices selected were identified from existing research literature (systematic reviews and clinical practice guidelines) using specific criteria.	No evidence<br><br>Use Brett's scoring to allow for comparability but notes that averaging the 8 practices for a final TIAB may not be appropriate.	Not reported	***Correlations with classification as a 'User'** ***(i.e., nurse who reported any use for at least 4 of the 6 practices)***<br><br>**Significant** ($p<0.05$)<br>(β; 95% CI)<br>• Aware overall (β=2.52; 0.98, 4.06)<br>• Aware by regular use (β=3.49; 2.47, 4.50)<br>• Persuaded of the appropriateness of the practice (β=2.11; 0.40, 3.83)<br><br>**Correlations with classification as a 'Consistent User'** ***(i.e., nurse who reported that they 'always use' 4 of the 6 practices)***<br><br>**Significant** ($p<0.05$)<br>(β; 95% CI)<br>• Perception of existence of policy/procedure (β=0.58; 0.09, 1.07)<br>• Work in critical care unit (CCU as reference group) (β= -0.42; -0.72, -0.12)
Thompson 1997 [35]	By expert panel of five doctoral-prepared nurses.<br>*(Unclear if practice only or if items were evaluated as well.)*<br><br>Content validity index (pilot) = .94	No evidence	Not reported	None reported
**RUQ n= 16 articles (14 studies)**				
Champion 1989 [55]	Expert panel	No evidence	Not reported	**Significant** ($p<0.05$)<br>• Attitude (r=0.55)

Study [citation in manuscript]	Content	Response Processes	Internal Structure	Relations with Other Variables
**Index**				• Availability (r=0.52)<br>• Multiple R=0.65 (accounts for 42% of the variance)<br><br>**Non-significant**<br>• Age<br>• Years employed
Bostrom 2006 [118]	Not reported	No evidence	Not reported	None reported
Bostrom 2007 [119]<br><br>*A report of Bostrom 2006*	State that the RUQ is valid<br><br>Details not provided<br>Cite:<br>Champion & Leach (1989)<br>Pettengill et al. (1994)<br>Humphris et al. (1999)	No evidence	Not reported	***Single item "*I use research findings in my daily practice*" from the RUQ used as the dependant variable for this study***<br><br>***Likert scale dichotomized into *agree* versus *do not know/disagree* to divide into research user vs. non-user groups for analysis***<br><br>**Significant** ($p<0.05$)<br>• Attitudes toward research (β=1.71; OR=5.52)<br>• Access to research findings at work place<br>• (β=1.90; OR=6.65)<br>• Support from unit manager (β=1.40; OR=4.03)<br><br>**Non-significant**<br>• Years of employment<br>• Age
Bostrom 2008 [120]	Not reported	No evidence	Not reported	**Significant** ($p<0.05$)<br>• Presentation subscale (i.e., RNs reporting more RU are less likely to perceive presentation of research as a barrier to RU) (r= -0.289)
Hansen 1999 [71]	Content & Predictive<br>Cite:	No evidence	Not reported	**Significant** ($p<0.05$)<br>(β coefficients are not reported)

Study [citation in manuscript]	Content	Response Processes	Internal Structure	Relations with Other Variables
	Champion & Leach (1989)			**<u>Physicians</u>** (All are measures of collaboration)<br>• Communication timeliness<br>• Within-unit coordination<br>• Communication openness between groups (47% of variance)<br>**<u>Nurses</u>**<br>• Communication openness within group (9.3% of variance)
Hatcher 1997 [72]	Not reported	No evidence	Not reported	**Significant** ($p<0.05$)<br>• Support (27% of variance when entered alone)<br>• Attitude (52% of variance when entered alone)<br>• Availability<br>• Significant difference between Nursing Advisory Committee (NAC) members (RU=4.03) & Staff Nurses (RU=3.27) (t=5.57, df=155).
Humphris 1999 [121]	Not reported	No evidence	Not reported	**Significant** ($p<0.05$)<br>(X^2 analysis; test statistic value not reported)<br>• Greater number of Diabetes Nurse Specialists (DNS) implement specific findings into practice as compared to the Non-Nurse Specialist (NNS) group (74% vs. 62%).
Humphris 2000 [122]	Judged by professional opinion. *(Not clear as to whether this is for the new measures or for RU.)*<br><br>*Reviewers' comment*: Although not reported as content validity, the authors developed the items for the questionnaire based on qualitative analysis from phase one of the study and from an extensive literature review.	No evidence	Not reported	None reported

Study [citation in manuscript]	Content	Response Processes	Internal Structure	Relations with Other Variables
Lacey 1994 [75]	Not reported	**Follow-up interviews**<br><br>Validity of self-reports in the questionnaire assessed by follow-up interviews. Respondents asked how they defined research utilization, to give examples of research-based practice in their own clinical area and about difficulties in implementing research findings.<br><br>Nurses were able to provide appropriate examples of research utilization and to interpret the term 'research' correctly (although they were unable to define the term 'research', could use the concept knowledgeably).<br><br>Many examples of research use given, indicating that it was likely that the respondents were accurately reporting their research use behaviour in the questionnaire	Not reported	**Significant** ($p<0.05$)<br>(β coefficients are not reported)<br>• Attitude<br>• Availability<br>• Support<br>(combined to account for 35.4% explained variance)

Study [citation in manuscript]	Content	Response Processes	Internal Structure	Relations with Other Variables
McCloskey 2005 [79]	Cite:<br>Champion & Leach (1989)- panel of experts	No evidence	Refer to Champion and Leach (1989)– factor analysis.<br><br>But no report of factor analysis in this reference.	**Significant** ($p<0.05$)<br>• Salary (β=0.003; R^2=.115)<br>• Direct (β=0.098; R^2=.164)<br>• Assets (β=0.113; R^2=.174)<br><br>**Variable details:**<br>***Salary*** : degree to which the nurse has paid work time to engage in research utilization activities<br>***Direct:*** support through direct consultation with mentors, statisticians, budgeted money, grants, and support for writing reports<br>***Assets:*** degree to which the respondent is able to use hospital resources such as supplies, services, equipment, and computers
McCloskey 2008 [123]<br><br>*A report of McCloskey 2005*	Cite<br>Champion and Leach (1989)- panel of experts	No evidence	Cite<br>Champion and Leach (1989)– factor analysis.<br><br>But no report of factor analysis in this reference.	**Significant** ($p<0.05$)<br>• Masters degree [vs. baccalaureate or associate/diploma] (*F*=11.34, df=2)<br>• Management position or advanced practice nurses [vs. staff nurses] (*F*=7.901, df=2)<br><br>**Non-significant**<br>• Years of nursing experience
Nash 2005 [81]	Not reported	No evidence	Not reported	**Significant** ($p<0.05$)<br>• Primary population (Mean RU score highest to lowest): critical (i.e., ICU, ER, surgery, or recovery), ambulatory, intermediate, long term, other (*F*=2.43)
Ohrn 2005 [82]	Not reported	No evidence	Not reported	**Significant** ($p<0.05$)<br>(t-test; test statistic value not reported)<br>• Education
Prin 1997 [85]	By three nursing informatics experts (process unclear)	No evidence	Not reported	**Significant** ($p<0.05$)<br>• Attitudes (r=.5793)
Tranmer 2002 [124]	Not reported	No evidence	Not reported	**Significant** ($p<0.05$)<br>**<u>All respondents (i.e., pretest + post test):</u>**

Study [citation in manuscript]	Content	Response Processes	Internal Structure	Relations with Other Variables
				• Attitude (β=0.63)<br>• Support (β=0.20)<br>• Access (β=0.20)<br><br>**Non-significant**<br>• Work experience<br>• Highest level of education [diploma vs. baccalaureate]
Wallin 2003 [89]	Not reported	No evidence	Not reported	None reported
**EROS n= 8 articles (7 studies)**				
Pain 1996 [125]<br><br>**Index**	Instrument developed based on focus groups.<br><br>4 sections of the EROS based on 90-minute focus group findings with representatives (4 clinicians, 4 clinician-researchers, and 3 administrators) from 7 institutions.<br><br>Informal discussions based on 4 questions, of which two ask about use.<br>1) In what ways (if any) have research findings influenced your own practice and that of others?<br>2) In the ideal world, how should research be integrated into clinical practice?<br><br>Findings, based on content	No evidence	Not reported for use subscale	None reported

Study [citation in manuscript]	Content	Response Processes	Internal Structure	Relations with Other Variables
	analysis, from this stage demonstrated that the concept of 'research orientation' went beyond specific changes in clinical procedures to include the four subcomponents.			
Bonner 2008 [126]	Not reported<br><br>Authors state that the EROS does not clearly define what research is and that this may limit validity	No evidence	Factor analysis with three retained components (45.1% explained variance in total):<br>1) Attitude (18.0%)<br>2) Use of Research (15.6%)<br>3) Knowledge of Research (11.4%)<br><br>But the analysis does not indicate which items are included in this scale. Further, there are four subscales in the original EROS.	**Significant** ($p<0.05$)<br>• Nurse unit managers and CN consultants as compared to nurses in other positions (H=12.67)<br>• Completion of a masters degree (H=11.16)
Henderson 2006 [127]	Not reported<br><br>Refer to Pain et al. 1996	No evidence	Not reported	None reported
McCleary 2002 [76]	Not reported	No evidence	Not reported for use subscale	**Significant** ($p<0.05$)<br>• Graduate [vs. baccalaureate or community college]

Study [citation in manuscript]	Content	Response Processes	Internal Structure	Relations with Other Variables
‘Use of the EROS...’	Refer to Pain et al.1996			(F=8.8, df=2172)<br><br>**Non-significant**<br>• Age<br>• Work experience
McCleary 2002 [77]<br><br>‘Research utilization among...’<br><br>*A report of McCleary 2002 above*	Not reported<br><br>Refer to Pain et al. 1996	No evidence	Not reported	None reported
McCleary 2003 [78]<br><br>*A report of McCleary 2002 above*	Not reported<br><br>Refer to Pain 1996	No evidence	Not reported	None reported
Pain 2004 [37]	Not reported	Utilize two methods to gain information about research utilization behaviours but the authors do NOT compare/contrast the findings from each of these methods as a way to assess the validity of the survey measure.	Not reported	None reported

Study [citation in manuscript]	Content	Response Processes	Internal Structure	Relations with Other Variables
Waine 1997 [128]	Not reported	No evidence	Not reported	None reported
**Specific practices n=5 articles (4 studies)**				
Aron 1990 [129]	Not reported<br><br>*Reviewer note:* Therapeutic techniques based on empirical research	No evidence	Not reported	None reported
Varcoe 1995 [50]	Total instrument- peer review *(Unclear if practice only or if items were evaluated as well.)*	Pilot testing with revision (whole instrument; nothing specific to the research use items).	Not reported	**Significant** (p<0.05)<br>• Climate (r=.33)<br>• Supportive infrastructures for research (e.g. libraries) (r=.31)<br><br>**Non-significant**<br>• Education
Knudsen 2004 [21]	Not reported<br><br>*Reviewer note*—the authors do report that the innovations were selected based on the literature, although research on acupuncture has mixed results, and were chosen to represent a wide range of approaches.	No evidence	Not reported	**Significant** (p<0.05)<br>(Structural equation model coefficients)<br>• Large size (0.290)<br>• Environmental scanning (information seeking in the external environment 4 items) (0.289)<br>• Collection of satisfaction data (from referral sources and third party payers 2 items) (0.140)
Tita 2005 [17]	Not reported	Respondents encouraged to add clarifying comments which enhanced validity for 3% of the sample (32.9% wrote in comments)<br><br>Debriefing allowed for	Not reported	None reported

Study [citation in manuscript]	Content	Response Processes	Internal Structure	Relations with Other Variables
		adjustment of prevalence ratios for 17 responses (3 for folic acid question which was misinterpreted and 14 for planned use of caesarean section which was likely over-reported)		
Tita 2006 [18]<br><br>*A report of Tita 2005*	Not reported	Refer to Tita 2005	Not reported	***Associated with at least 50% variation in awareness of practice***<br><br>**Significant** (p<0.05)<br>• Awareness is associated with a 15-fold increase in practice (PR=15.4; 95% CI: 4.3-55)<br>• Have internet access (aPOR=prevalence odds ratio adjusted for other confounders retained in the logistic regression model=3.4)
**General Research Utilization Indices n=10 articles (10 studies)**				
Forbes 1997 [101]	Not reported	No evidence	Factor Loadings for RU subscale:<br>Item29=.68<br>Item30=.80<br>Item31=.82<br>Item32=.72<br>Item33=.66	**Significant** (p<0.05)<br>• Group cohesion (r=.07)<br><br>**Significant** (p<0.05)<br>(post-hoc Duncan test; test statistic value not provided).<br>• Difference between groups for critical care nurses (M=9.2, SD 3.1) as compared to medical/surgical (M=8.2, SD 3.0) or obstetrical/gynecological (M=8.7, SD 3.0)
Grasso 1988 [102]	Index score results from sum of first 9 items; One item omitted from index score based on content analysis that it is	No evidence	Not reported	**Significant** (p<0.05)<br>• Perception of facilitators=27.7% (r=.51)<br>• Pro-research attitudes= 3.5% (r=.39)

Study [citation in manuscript]	Content	Response Processes	Internal Structure	Relations with Other Variables
	different from the remaining items.			
Kamwendo 2002 [105]	Cite:<br>Eckerling et al. (1988)-four dimensions and the four research activities chosen based on an extensive literature review	Trialed the instrument with 30 physiotherapy colleagues and students resulting in minor changes	Not reported	***Correlations to engagement dimension for 'apply research findings' activity***<br><br>**Significant** ($p<0.05$)<br>(Spearman coefficient)<br>The following are work-related factors:<br>• I have set time aside to read (0.14) or to execute research (0.24)<br>• Research activities are encouraged by physiotherapist colleagues (0.15), by other colleagues (0.22), by nearest superior (0.12), by management (0.19)
Karlsson 2007 [73]	Cite:<br>Eckerling et al. (1988)-four dimensions and the four research activities chosen based on an extensive literature review	No evidence	Not reported	***Correlations to engagement dimension for 'apply research findings' activity***<br><br>**Significant** ($p<0.05$)<br>(Spearman coefficient 1997; 2003)<br>The following are work-related factors:<br>• I have set time aside to read (0.21; 0.13) or to execute (0.24; 0.11)<br>• Research activities are encouraged by OT colleagues (0.27; 0.18), by other colleagues (0.24; 0.14), by nearest superior (0.24; 0.10), by management (0.21; 0.10)
Morrow-Bradley 1986 [103]	Not reported	No evidence	Not reported	***Correlations to the utility index***<br><br>**Significant** ($p<0.05$)<br>(Kendel tau)<br>Agree that:<br>• Clinically meaningful questions are not studied (-0.15);<br>• Criteria are either too global or too specific (-0.13);<br>• Research procedures distort therapeutic process (-0.21);<br>• Studies ignore complexities of therapy (-0.14);

Study [citation in manuscript]	Content	Response Processes	Internal Structure	Relations with Other Variables
				• Therapeutic relationship is ignored (-0.13);<br>• Total # criticisms endorsed (-0.19)
Pelz<br>1981 [84]	Not Reported	No evidence	Not reported	**Significant** ($p<0.05$)<br>(Paired t-tests; test statistic value not reported)<br>• Experimental IT members (in intervention hospitals) increased significantly in direct measures of RU from year 1 to year 2 (i.e. post-intervention)<br>• Experimental ITs significantly higher than experimental non-IT counterparts on 5 of 6 direct RU measures (includes RU index score) in year 2 and on 4 of the 6 RU scores in year 3. In both cases, changes to mean RU index was significant.
Rardin<br>1986 [104]	Not reported	Pre-tested by 3 graduate students in counseling psychology to ensure clarity and to refine the layout	Not reported	None reported
Reynolds<br>1981 [24]	Not reported<br><br>*Reviewer note:* The following general rules for constructing the indices from the survey items to represent the concepts in the study were used:<br>1) Constituent items must be conceptually related<br>2) Constituent items must be statistically related<br>3) That statistical relationships must be stable within each of the nursing roles<br>4) The final result was to be a 4	Pre-tested on a group of nurses employed in a non-participating hospital. Changes made in format and wording.	Not reported	**Non-significant** (NS)<br>(Covariance analysis)<br>• Professionalism (total standardized coefficient=0.498 NS; individual component=0.456 NS; organizational component=0.042 NS)<br>• Decentralization (total standardized coefficient=0.333 NS; individual component=0.079 NS; organizational component=0.254 NS)<br>• Communication (total standardized coefficient= -0.090 NS; individual component=0.209 NS; organizational component= - 0.299 NS)<br>• Size<br>Note: Size does demonstrate significant effects with communication and decentralization (total standardized coefficient= -0.042 NS; individual component=none;

Study [citation in manuscript]	Content	Response Processes	Internal Structure	Relations with Other Variables
	to 6 category variable with each category typically containing 20 to 25 percent of the cases<br>5) For respondents with missing data on a constituent item, an index value was constructed using the available data if at least half of the data were available.			organizational component= - 0.042 NS)<br>• Interorganizational relationships (total standardized coefficient= - 0.045 NS; individual component=none; organizational component= - 0.0.045 NS)
Stiefel 1996 [36]	Pilot—by 4 NRU experts (2 members of the CURN project, 1 developer of the Iowa model, and 1 who works actively with nurses on NRU projects)<br><br>Current study—Clinical nurse researcher at the Midwest site<br><br>Process for the expert review unclear in both cases	No evidence	**Below are from the pilot**<br><br>Factor analysis (n=202 RNs)<br>Loading of 3 factors<br>1) Literature factor (7 items): 0.56-0.88<br>2) Intervention factor (8 items): 0.43-0.74<br>3) Outcomes factor (3 items): 0.57-0.65<br>2 items were deleted from the original 20 item survey<br><br>No discussion as to why did not use the findings from	**Significant** ($p<0.05$)<br>• Critical care (vs. other settings)<br>(Wilk's lambda=0.76, F=2.23, df=1246)

Study [citation in manuscript]	Content	Response Processes	Internal Structure	Relations with Other Variables
			the factor analysis in the derivation of the final score- FA showed 3 factors but they presented 1 derived score	
Varcoe 1995 [50]	Total instrument- peer review *(Unclear if practice only or if items were evaluated as well.)*	Pilot testing with revision (whole instrument; nothing specific to the RU items)	Not reported	**Non-significant**<br>• Education (diploma vs. degree)<br>• Value research
**Knott & Wildavsky Standards n=1 article (1 study)**				
Belkhodja 2007 [20]	Not reported<br><br>*Reviewer note*: Based on the utilization scales developed by Knott & Wildavsky (1980)	No evidence	Not reported	**Significant** ($p < 0.05$)<br>• Research relevance (t-ratio=3.668)<br>• Organizational culture (research as a preferred source [i.e., research culture] (t-ratio=3.621); intensity of research source use (t-ratio=3.506)<br>• Formal linkage mechanisms (t-ratio=7.142) and informal linkage mechanisms (t-ratio=3.251)
**Past/ Present/ Future Utilization n= 3 (3 studies)**				
Brown 1997 [130]	Not reported	No evidence	N/A	***Correlations to interest in future participation:** ***Applying research findings to practice*****<br><br>**Significant** ($p<0.05$)<br>• Higher education (without bachelor's vs. with bachelor's vs. graduate degree) (X^2=36.1, V=0.14)
Butler 1995 [65]	Not reported	No evidence	N/A	**Significant** ($p<0.05$)<br>**<u>Staff group:</u>**<br>• Perceived support within the system for research activity (X^2=4.88, OR=2.0)<br>• Nurses in the leadership group are five times more likely than staff nurses to use research (OR=5.01)

Study [citation in manuscript]	Content	Response Processes	Internal Structure	Relations with Other Variables
				**Non-significant**<br>**<u>Staff group</u>**<br>• Age<br>• Experience as RN<br>**<u>Leadership group:</u>**<br>• Age<br>• Experience as RN<br>• Education
Wells<br>1994 [90]	Not reported	No evidence	N/A	**Significant** ($p<0.05$)<br>• Higher research value (β=1.62)
**Parahoo n= 7 articles (3 studies)**				
Parahoo<br>1998 [53]<br><br>**Index**	Panel of three experts<br><br>Questionnaire developed after a review of the literature on research utilization and research activities<br><br>No details provided.	Reports a pilot with 20 nurses--changes were made mostly to wording. Not clear as to whether this was for research use items or not.	N/A	None reported
Parahoo<br>1999 [131]<br>'A comparison…'<br><br>*A report of Parahoo, 1998*	Panel of three experts<br><br>Questionnaire developed after a review of the literature on research utilization and research activities<br><br>No details provided.	No evidence	N/A	None reported
Parahoo, 1999	Panel of three experts	Reports a pilot with 20	N/A	None reported

Study [citation in manuscript]	Content	Response Processes	Internal Structure	Relations with Other Variables
[132] 'Research utilization...'<br><br>*A report of Parahoo, 1998*	Questionnaire developed after a review of the literature on research utilization and research activities<br><br>No details provided.	nurses--changes were made mostly to wording.		
Parahoo 2000 [134]<br><br>*A report of Parahoo, 1998*	Panel of three experts<br><br>Questionnaire developed after a review of the literature on research utilization and research activities<br><br>No details provided.	Reports a pilot with 20 nurses--changes were made mostly to wording.	N/A	None reported
Parahoo 2001[133]<br><br>*A report of Parahoo, 1998*	Panel of three experts<br><br>Questionnaire developed after a review of the literature on research utilization and research activities<br><br>No details provided.	Reports a pilot with 20 nurses--changes were made mostly to wording.	N/A	None reported
Valizadeh 2003 [135]	Survey was translated into Persian and back-translated into English for analysis but no report on whether content validity was re-established during this process<br><br>Parahoo 1999-expert panel	Not reported. Refer to Parahoo 1999-pilot study	N/A	None reported

Study [citation in manuscript]	Content	Response Processes	Internal Structure	Relations with Other Variables
Veeramah 2004 [136]	Questionnaire developed following a review of the literature<br><br>Reviewed by a panel of five nurse or midwifery teachers with expertise in research methods	Piloted with 12 graduates similar to the intended sample. Changes made mostly to wording	N/A	None reported
**Estabrooks' Kinds of Research Utilization n= 10 articles (8 studies)**				
Estabrooks 1999 [3] 'The conceptual…'	Reviewed by two researchers with expertise in the field.<br><br>Careful attention paid to theoretical conceptualizations of research utilization in the literature, questioning approaches of previous investigators, theoretical needs of the study, and the investigator's clinical experience.<br><br>Participants were deliberately coached with definitions and examples. Participants' responses to the question on general research use should have reflected the acquisition of new knowledge about the concept of 'research utilization' as they progressed through the questionnaire.	Pilot testing on a convenience sample (n=23) of post-basic baccalaureate nursing students and master's nursing students. The labeling convention was chosen as pilot testing suggested that concrete labels were required to make explicit that the numerical scale was a relative scale.	N/A	**Significant** (Variables retained in final model structural equation model fit using LISREL X^2=55.91, p=0.263, AGF=0.956)<br>(Coefficients are not reported)<br>• Attitude towards research<br>• Attending in-services

Study [citation in manuscript]	Content	Response Processes	Internal Structure	Relations with Other Variables
Estabrooks 1999 [68] 'Modeling…'<br><br>*A report of Estabrooks 1999* [3]	See report 1999a	See report 1999a	N/A	**Significant** (Variables retained in final model structural equation model fit using LISREL X^2=55.91, p=0.263, AGF=0.956)<br>(Coefficients are not reported)<br>• In-services attended in the past year<br>• Attitude towards research
Kenny 2005 [74]	Not reported	No evidence	N/A	**Regression models** ($p<0.10$)<br>(β Coefficients not reported)<br><br>*Direct Research Use*<br>• Attitude<br>• Access<br>• Organizational support<br><br>*Persuasive Research Use*<br>• Access<br><br>*Overall Research Use*<br>• Organizational innovativeness
Estabrooks 2007 [69]	Not reported	No evidence	N/A	**Significant** ($p<0.05$)<br>(Cumulative logit modeling)<br>**Canadian Civilian Sample** (OR; 95% CI)<br>*Overall Research Use:*<br>• Presence of a research champion (1.47; 1.03,2.10)<br>• Number of in-services (1.03; 1.01,1.06)<br>• Attitude (1.21; 1.13,1.30)<br><br>*Instrumental Research Use:*<br>• Library access (0.95; 0.90,1.00)<br>• Attitude (1.17; 1.09,1.25)<br><br>**US Army Sample** (OR; 95% CI)

Study [citation in manuscript]	Content	Response Processes	Internal Structure	Relations with Other Variables
				Overall Research Use:<br>• Attitude (1.16; 1.06-1.14)
Estabrooks 2008 [26]<br><br>*A report of Profetto-McGrath 2003*	Not reported	'Overall research utilization' is the only *Research Use* question reported here as it was the only one that maintained reliability when aggregated to the unit level. It was asked three times. The scores increased significantly between the first and second and the second and third repetition.<br><br>Adjusted overall RU scores were obtained by taking a weighted average of the score obtained from the three repetitions: time one (1/6), time 2 (2/6), time 3 (3/6). Higher weights assigned as participants learned more about RU over the course of questionnaire completion and authors reasoned that latter responses are more representative of the true 'overall RU' score.	N/A	None reported
Profetto-	Refers to Estabrooks 1997:	No evidence	N/A	Report construct validity by Estabrooks 1999: development

Study [citation in manuscript]	Content	Response Processes	Internal Structure	Relations with Other Variables
McGrath 2003 [70]	appraised by experts in the field			of a model explaining the conceptual structure of RU using these measures
Cobban 2008 [66]	Not reported	Pre-tested with a convenience sample of dental hygiene clinical instructors re: clarity and ease of completion<br><br>This was reported as content validity in the article.	N/A	None reported
Connor 2006 [67]	Not reported	Pilot study with 6 individuals from each of the three groups. Found no problem with design. Used to refine data collection procedures, 2 questions were clarified but not RU questions.	N/A	**Significant** ($p<0.05$)<br>**RN**<br>*Overall Research Use*<br>• Access (to sources within the organization) (β=0.054)<br>• Attitude (β=0.117)<br><br>**LPN**<br>*Indirect Research Use*<br>• # of in-services (only factor in the model) (R^2=0.237)<br><br>*Persuasive RU*<br>• Access (β=0.122)<br><br>*Overall RU*<br>• Support (only factor in the model) (R^2=0.194)<br><br>**PCW/CCA**<br>*Direct Research Use*<br>• Attitude (only factor in the model) (R^2=0.070)<br><br>*Indirect Research Use*<br>• Attitude (β=0.114)

Study [citation in manuscript]	Content	Response Processes	Internal Structure	Relations with Other Variables
				Persuasive Research Use<br>• Access (β=0.059)<br><br>*Overall Research Use*<br>• Attitude (only factor in the model) (R^2=0.224)
Milner 2005 [80]	Not reported	No evidence	N/A	**Significant difference between groups** ($p<0.05$)<br>(One-way ANOVA; test statistic value not reported)<br>• Clinical nurse educators > staff nurses for all measures of research utilization<br><br>**Significant coefficients** ($p<0.05$)<br>(Regression model; R^2=39%)<br>*Overall Research Utilization*<br>• Attitude (β=0.098)<br>• Awareness (β= 0.063)<br><br>*Conceptual Research Utilization*<br>• Localite (use of local sources of information in clinical practice (e.g., colleagues) (β= 0.031)<br><br>*Symbolic Research Utilization*<br>• Mass media (use of mass media sources of information in clinical practice) (β= 0.194)
Profetto-McGrath 2008 [86]	State that validity has been reported elsewhere (Estabrooks 1999)	No evidence	N/A	None reported
**Other Single-Item Measures n= 39 articles (39 studies)**				
Barwick 2008 [23]	Not reported<br><br>*Reviewer note*: Based on the Canadian Health Services Research Foundation's Four-A's	No evidence	N/A	None reported

Study [citation in manuscript]	Content	Response Processes	Internal Structure	Relations with Other Variables
	approach: access, assess, adapt, apply but no panel review			
Bjorkenheim 2007 [49]	Not reported	No evidence	N/A	None reported
Callen 2005 [137]	Not reported	Pilot with 7 medical practitioners and 5 Sydney university academics; modifications made.	N/A	None reported
Cameron 2005 [138]	Based on design used by Humphris et al. (2000) and evaluated by 3 expert reviewers. Process unclear.<br><br>They took items from the RUQ but have broken up the scale and have used single items.	No evidence	N/A	None reported
Dobbins 2001 [51]	Modified from previous diffusion of innovation and research utilization studies	Face-established during pre-test at 1 Public Health Unit	N/A	**Significant** ($p<0.05$)<br>• Perception that the systematic reviews (SRs) could overcome the barrier of limited critical appraisal skills ($r=0.23$)<br>• Perception of SRs as easy to use ($r=0.14$)
Dysart 2002 [38]	Not reported	No evidence	N/A	None reported
Elliott 2008 [139]	Not reported<br><br>*Reviewer note*: data available from previous studies and	No evidence	N/A	***Item=Utilize research findings as a result of having a research paper published****<br><br>**Significant** ($p<0.05$)

Study [citation in manuscript]	Content	Response Processes	Internal Structure	Relations with Other Variables
	focus-group interviews with sonographers informed the composition of the questionnaire.<br>Nothing specific to RU questions and no expert panel.			(X^2 test; test statistic value not reported)<br>• Master degree
Erler<br>2000 [140]	Not reported	No evidence	N/A	***Item:=Performing literature searches***<br><br>**Significant** (p<0.05)<br>• Chief flight nurse or research nurse (X^2=15.7)<br><br>***Item=Translating research findings into policies and procedures***<br><br>**Significant** (p<0.05)<br>• Role (X^2=25.0)
Ersser<br>2008 [39]	Not reported	No evidence	N/A	None reported
Heathfield<br>2000 [40]	Not reported	No evidence	N/A	None reported
Kelly<br>2008 [41]	Not reported	No evidence	N/A	None reported
Kirk<br>1976 [28]	Not reported	No evidence	N/A	**Significant** (p<0.05)<br>• Attitude (Index composed of 5 items) (r=0.25)
Logsdon<br>1998 [141]	Based on the literature and the investigator's experience with research use in the clinical setting	No evidence	N/A	None reported
Meehan<br>1988 [22]	Not reported	Self-report measure was supplemented by:<br>1) Asking all respondents	N/A	None reported

Study [citation in manuscript]	Content	Response Processes	Internal Structure	Relations with Other Variables
		to identify and describe how studies they believed had a major impact had been used<br>2) Asking selected respondents how specifically selected studies had been used<br><br>Interview guides (logic, flow, timing, economy, and rationale for each question) were pretested with simulated sessions with 4 individuals (2 of these for the client set of questions, 2 for researcher/manager set)<br><br>Similar questions included within the 'General HSR results use' section to check consistency of responses		
Miller 2007 [142]	Not reported	Draft version reviewed by 2 professors within the University clinic regarding ambiguity and organization. Revisions were made.	N/A	None reported
Molassiotis 1997 [25]	Not reported	Those who report that they incorporated	N/A	None reported

Study [citation in manuscript]	Content	Response Processes	Internal Structure	Relations with Other Variables
		research findings into practice also provided specific examples of the research subject matter that they were using, including using research about *clostridium difficile*, pain relief, oral assessment guides, treatments for oral mucositis, and findings about primary care nursing systems.		
Mukohara 2005 [42]	Not reported	No evidence	N/A	None reported
Nelson 2007 [143]	Not reported	No evidence	N/A	**Significant** ($p<0.05$)<br>• Openness of clinical setting ($\beta=0.21$)<br>• Positive attitudes ($\beta=0.28$)<br>• Negative attitudes ($\beta= -0.19$)
Niederhauser 2005 [43]	Not reported	No evidence	N/A	None reported
Ofi 2008 [144]	Content—experts in the field	No evidence	N/A	**Non-significant**<br>• Education (diploma vs. degree)
Olade 2004 [83]	Verified by two doctoral nurses, two RNs and a sociologist	Respondents added comments. These give the reader some indication as to what respondents interpret as 'research utilization':<br>• "What we do is the	N/A	None reported

Study [citation in manuscript]	Content	Response Processes	Internal Structure	Relations with Other Variables
		result of some kind of research"<br>• "Most of the findings that I have used, I found experimentally works"<br>• "Common sense tells me that the findings in medical research are used daily, but I have not directly taken part in any research"<br>• "Our nursing policies are research-based and updated yearly with current literature"		
Oliveri 2004 [145]	Not reported	No evidence	N/A	**Non-significant**<br>• Academic degree
Olympia 2005 [44]	Not reported	No evidence	N/A	None reported
Pain 2004 [37]<br><br>*Interview results*	Not reported	Utilize two methods to gain information about research utilization behaviours but the authors do NOT compare/contrast the findings from each of these methods as a way to assess the validity of the survey measure.	N/A	None reported

Study [citation in manuscript]	Content	Response Processes	Internal Structure	Relations with Other Variables
Pettengill 1994 [54]	Not reported	Nurse educators and 2 nurse administrators (authors report this as content validity)	N/A	None reported
Pepler 2005 [27]	Not reported	Data from multiple sources were used to illustrate the presence or absence of research use. For example, in addition to interviews, field notes (based on direct observation) were kept in relation to ongoing practices observed on each unit. Issues such as how nurses dealt with the situations when they needed information or how new ideas evolved were pursued. Data also collected on resources and their use.	N/A	***Principal factor linked to high research use***<br><br>• Unit culture (as consisting of harmony of research perspective, motivation to learn, goal orientation, creativity, critical inquiry, mutual respect, maximization of resources)
Scott 2000 [45]	Not reported	No evidence	N/A	None reported
Sekerak 1992 [87]	Not reported<br><br>*Reviewer note:* Although not discussed in the context of validity, questionnaire was developed with input from expert reviewers	Questionnaire was developed with input from pilot subjects (n=5)	N/A	None reported

Study [citation in manuscript]	Content	Response Processes	Internal Structure	Relations with Other Variables
Stetler 1991 [88]	Interview schedule developed based on literature review	Small pilot with 4 masters prepared nurses to critique the tools. Changes made based on consistent feedback.<br><br>Post-hoc analysis of reported sources of research-based information to determine whether they were actually research-based. Could document at least one source by 71% of the CNSs; of these, 79.5% could be categorized as research-related<br><br>To guard against the related problems of memory and social desirability the following precautions were taken:<br>---Subjects given the opportunity to process their research use through a specific case prior to completing scales on routine use<br>---Scale had an acceptable non-use option (i.e., considered but rejected)	N/A	None reported

Study [citation in manuscript]	Content	Response Processes	Internal Structure	Relations with Other Variables
		---Post-hoc validation of actual citations		
Suter 2007 [52]	Informed by in-depth interviews with 8 complementary and alternative medicine (CAM) practitioners<br><br>Face-assessed by an expert in CAM research literacy	Pilot tested by 5 CAM and conventional practitioners	N/A	*** Item =*I apply research findings in my practice****<br>**Significant** ($p<0.05$)<br>(Logistic regression)<br>• Research adds credibility to my discipline (OR 1.6)
Sweetland 2001 [146]	Based on the literature and two exploratory interviews with expert occupational therapists in the field of stroke rehabilitation	No evidence	N/A	None reported
Tsai 2000 [57]	Expert panel of 8 nurses prepared at masters and doctoral levels	Pilot test to ensure the tool content was associated with other data reported in the literature and was sensitive to the symbolic meanings relevant in Taiwan's nursing community	N/A	None reported
Tsai 2003 [58]	Refer to Tsai 2000	Tool checked and confirmed by five clinical nurses	N/A	None reported
Upton 1999 [46]	Not reported	No evidence	N/A	None reported
Veeramah 1995 [147]	Not reported	Pilot tested with Research Interest Group Nurses –Do not	N/A	None reported

Study [citation in manuscript]	Content	Response Processes	Internal Structure	Relations with Other Variables
		report number or whether they evaluated the content		
Veeramah 2007 [47]	Not reported	No evidence	N/A	None reported
Walczak 1994 [48]	Research activities scale-documented by Stetler (1983, 1985), other research related literature (American Nurses Association Commission on Nursing Research (1981), and the investigators' experiences<br><br>Authors report that items measuring 'using research' may not have been specific enough. They suggest revising items in the future to ask more specifically about how research is used (e.g., As a foundation for assessing a problem, to develop assessment tool, as a basis for nursing standard and protocols)	No evidence	N/A	None reported
Wood 1996 [148]	Not reported	2 internists reviewed and critiqued the questionnaire throughout its development<br><br>Pre-tested by 5 members	N/A	***Item=*Over the past month, to about how many patients have you applied EBM to answer a clinical question?**** <br>None reported

Study [citation in manuscript]	Content	Response Processes	Internal Structure	Relations with Other Variables
		of the ACP.<br><br>Provided few substantive comments on content and items were found to be clear.		
Wright 1996 [149]	Not reported	Consultation with 3 clinical nurse consultants	N/A	None reported
